# Topologically Precise and Discrete Bottlebrush Polymers: Synthesis, Characterization, and Structure–Property Relationships

**DOI:** 10.1021/jacsau.2c00010

**Published:** 2022-03-18

**Authors:** Nduka D. Ogbonna, Michael Dearman, Cheng-Ta Cho, Bhuvnesh Bharti, Andrew J. Peters, Jimmy Lawrence

**Affiliations:** † Department of Chemical Engineering, Louisiana State University, Baton Rouge, Louisiana 70803, United States; ‡ Department of Chemical Engineering, Louisiana Tech University, Ruston, Louisiana 71272, United States

**Keywords:** polymerization kinetics, precision bottlebrush polymers, glass transition temperature, discrete macromonomer, discrete bottlebrushes

## Abstract

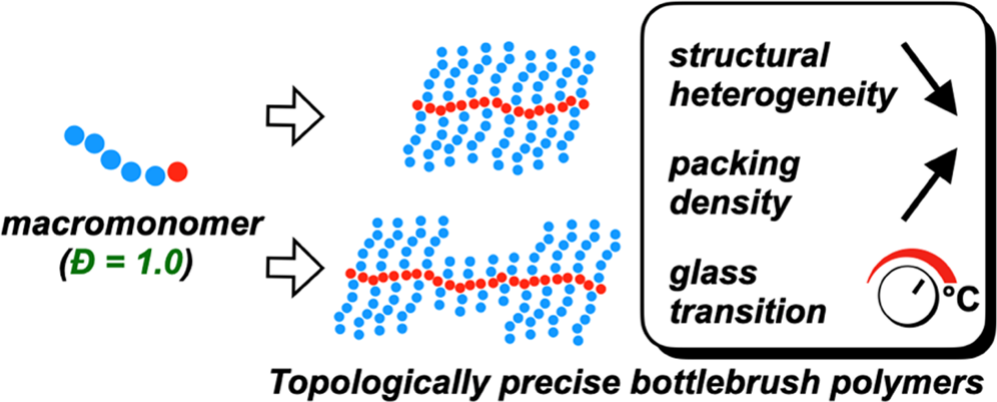

As the complexity of polymer structure grows, so do the challenges for developing an accurate understanding of their structure–property relationships. Here, the synthesis of bottlebrush polymers with topologically precise and fully discrete structures is reported. A key feature of the strategy is the synthesis of discrete macromonomer libraries for their polymerization into topologically precise bottlebrushes that can be separated into discrete bottlebrushes (*Đ* = 1.0). As the system becomes more discrete, packing efficiency increases, distinct three-phase Langmuir−Blodgett isotherms are observed, and its glass transition temperature becomes responsive to side-chain sequence. Overall, this work presents a versatile strategy to access a range of precision bottlebrush polymers and unravels the impact of side-chain topology on their macroscopic properties. Precise control over side chains opens a pathway for tailoring polymer properties without changing their chemical makeup.

## Introduction

**Scheme 1:**
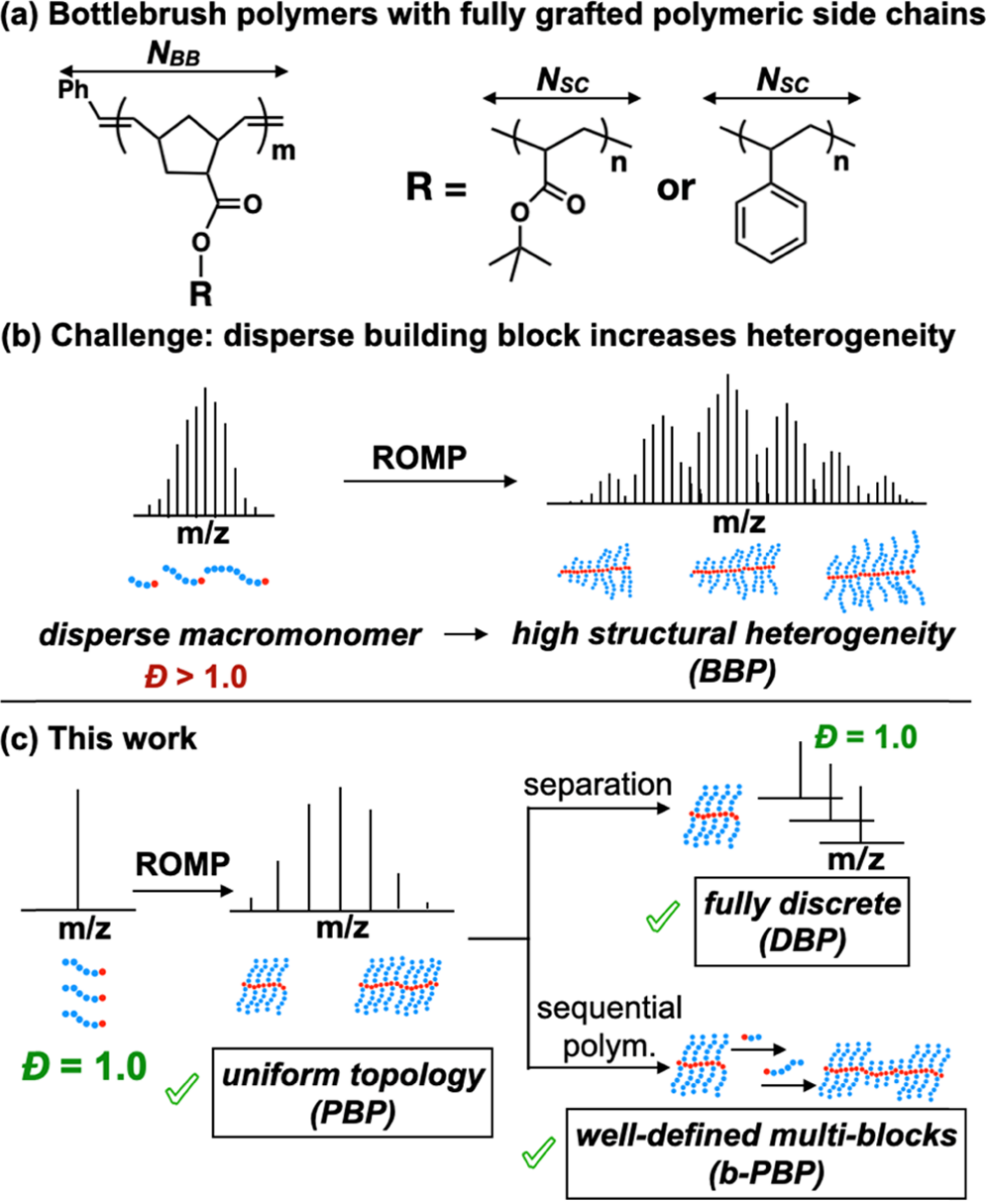
Synthesis of Topologically Uniform, Discrete, and Block Bottlebrush Polymers *Đ*: dispersity index, *N*_BB_: backbone degree of polymerization, *N*_SC_: side chain degree of polymerization.

Branched polymers such as bottlebrush polymers (BBPs, Scheme 1a) are promising material platforms for ultrasoft interfaces,^1−3^ sensing,^4−6^ and delivery applications^7−9^ because they possess unique entanglement-free rheology and high functional group density.^10−13^ In the past decade, the synthesis of complex BBPs with myriad polymeric side-chain architectures has been enabled by the development of grafting-to, -from, and -through strategies.^14−17^ However, such multifunctional BBPs have disperse backbone and side chains, and thus, their properties reflect a broad distribution of species, not the individual brushes. Each layer of dispersity introduced in the BBP synthesis amplifies the number of species in the mixture in an exponential manner (Scheme 1b). This challenge is further compounded by limitations for estimating BBP structure. For BBPs prepared by grafting-through, the backbone degree of polymerization (*N*_BB_) is calculated by dividing its total number averaged molecular weight (*M*_n_) from light scattering analysis by the *M*_n_ of the disperse macromonomer. Given the side chain length is not identical across the backbone length of BBP,^18−20^ understanding the impact of structural precision on BBP heterogeneity and properties remains a grand challenge.

Accurate insights into the structure–property relationship of bottlebrush polymers are crucial for their rational design. Inspired by the well-defined structure and functional precision of aggrecan,^21,22^ a bottlebrush-like biopolymer, we synthesized a library of topologically uniform (‘truly cylindrical’^23^) precision bottlebrush polymers (**PBPs**, *Đ*_SC_ = 1.0) using discrete macromonomers (Scheme 1c). Previous studies have shown that discrete oligomers and polymers exhibit strong length-dependent properties distinct from their disperse counterparts.^24−33^ Without controlling side-chain dispersity, the permutation of side-chain arrangements in conventional BBPs imposes a significant challenge for understanding their structure–property relationships,^34^ especially, at the underexplored regime of low *N*_BB_ and *N*_SC_.^35,36^

Here, we report that both the properties and topology of BBPs are significantly impacted by the dispersity of their side chains. To date, only grafting-through techniques such as ring-opening metathesis polymerization (ROMP) can yield BBPs with fully grafted polymeric/bulky side chains.^37−39^ However, the rapid kinetics of ROMP can lead to the assumption that all macromonomer species polymerize at a similar rate. Rather, we posited the polymeric macromonomer size affects their propagation rate constant (*k*_p_), with the resulting bottlebrush topology being significantly impacted by macromonomer dispersity, especially for *N*_SC_ < 10. Corroborating our hypothesis, *k*_p_ of disperse polymeric macromonomers was found to approach a constant value only when *N*_SC_ approaches 100.^40^ A significant polymerization rate difference between macromonomer species in disperse mixtures raises the concern of topological accuracy and structural heterogeneity in disperse BBPs. The polymerization of discrete macromonomers described in this study addresses this challenge because it yields Tetris-like bottlebrushes with uniform and tailorable side-chain topology. Importantly, the well-defined structure of PBPs opens access to truly discrete bottlebrush polymers (**DBPs**, *Đ*_BB_ & *Đ*_SC_ = 1.0). As we show later, a versatile and scalable strategy to prepare a range of PBPs in linear and block topology enables us to examine the impact of side-chain dispersity on the physical and thermomechanical properties of brush polymers. The broad scope of this strategy was also illustrated through the synthesis of PBPs and DBPs from other monomer families.

## Results and Discussion

### Synthesis of Macromonomers and Bottlebrush Polymers

Standard controlled polymerization and chain end-functionalization strategies were employed to prepare disperse macromonomers. The disperse parent materials were then separated into a library of discrete macromonomers (see the Supporting Information).^26,31,35,41−45^ This multistep approach is critical for removing traces of nonfunctional byproducts and impurities.^46^ Vinyl monomers were chosen for this study because of their wide monomer scope, synthetic versatility, and potential relevance toward other bulky macromonomers (e.g., branched alkyl, conjugated).^47^ A polymeric side chain of *N*_SC_ ≈ 10 is comparable to C20 alkyl side chain, lengthwise.

All macromonomers and bottlebrush polymers synthesized in this study were characterized using NMR, size-exclusion chromatography (SEC), and MALDI-ToF (Table S1). As an example, ^1^H NMR analysis confirmed the quantitative conversion of tetrameric bromine terminated oligo(*tert*-butyl acrylate) (oTBA4-Br) into norbornenyl-oTBA4 (**NB-oTBA4**), as seen in the downfield shift of the chain-end methine protons from 4.1 to 4.8 ppm and the appearance of the norbornenyl cyclic alkene proton at 6.1 ppm. Ideal integration ratios were seen for all characteristic peaks of discrete macromonomers (Figures S3–S5). The structural purity of discrete macromonomers was confirmed using SEC and MALDI-ToF (single molecular peak, calculated for **T4** = 664.42, observed = 664.48, Figures S22 and S23).

**Figure 1:**
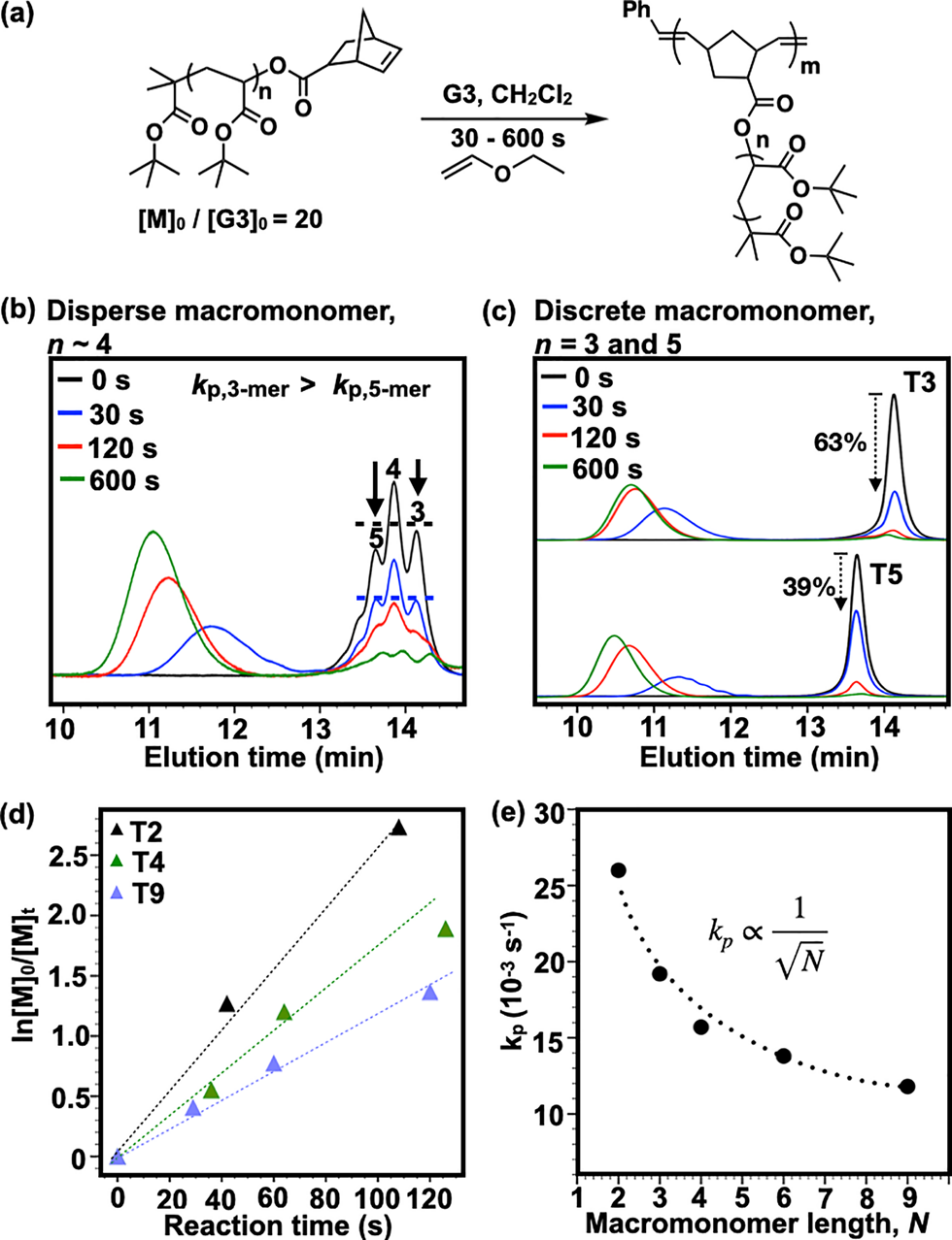
(a) Bottlebrush polymer synthesis via grafting-through ROMP. (b) SEC RI traces of ROMP of disperse tetramer, **NB-otBA4**, at various reaction times. (c) SEC traces of ROMP of discrete trimer, **T3** and pentamer, **T5**. (d) In [M]_0_/[M]_t_ vs polymerization time for discrete macromonomers. (e) *k*_p_ vs length of the discrete macromonomers, T2–T9. The total peak areas of all SEC traces were normalized to unity.

With disperse and discrete macromonomers in hand, bottlebrush polymers with tailored *N*_BB_ and *N*_SC_ were synthesized using pyridine-ligated Grubbs third generation catalyst (G3) (Figure 1a). G3 catalyst is well-suited for kinetic studies as initial screening confirmed its slower polymerization rate relative to other G3 variants (∼20% of 3-bromopyridine-ligated G3), in agreement with the literature.^48^ The averaged total molecular weight of BBPs and PBPs were estimated using SEC equipped with a multiangle light scattering detector, and the molecular mass of unimolecular DBPs was determined using MALDI-ToF. A stochastic model was constructed to simulate the molecular weight distributions of the BBP and the distribution of side chains along the backbone (see the Supporting Information).

### Impact of Macromonomer Dispersity on Polymerization Kinetics

Through examining the grafting-through ROMP kinetics of disperse and discrete macromonomers, we found the propagation rate constant (*k*_p_) of short macromonomers (*N*_SC_ < 10) to be inversely proportional to the square root of their chain length. While length-dependent propagation rate behavior was recently observed for disperse macromonomers with *N*_SC_ up to 50,^40^ we hypothesized that the pronounced impact of dispersity, especially on short polymer chains, will necessitate the use of discrete systems to obtain accurate and reproducible *k*_p_ values. Following standard procedures, macromonomer conversion was calculated by analyzing the NMR spectra and SEC traces^43,49^ of aliquots taken during the polymerization of macromonomers (Figures S28 and S29). Deconvoluting the SEC trace of disperse system using a Gaussian fitting algorithm showed that within 30 s, the trimer macromonomer is consumed ∼25% faster than the pentameric species (Figure 1b). This finding attracted our attention because the propagation kinetics of norbornenyl macromonomers under ROMP condition is fast and less sterically hindered, and therefore the controlled chain growth process with increasing macromonomer conversion is often attributed to similar macromonomer reactivity.^50^

To obtain accurate propagation rate constants, we synthesized a library of discrete macromonomers (dimer to nonamer, **T2–T9**) and examined their homopolymerization kinetics. The trimeric T3 polymerizes 50% faster than the pentameric T5 (Figure 1c), and the propagation rate of T2 is ∼220% the rate of T9 (*k*_p,T2_ = 26 × 10^–3^ s^–1^, *k*_p,T9_ = 11.8 × 10^–3^ s^–1^, Figure 1d). The polymerization of each discrete macromonomer maintains its living characteristics, as seen in their pseudo-first-order kinetics and the linear relationship between ln[M]_0_/[M]_t_ and polymerization time (Figure 1d). Our kinetic analysis suggests that the polymerization rate of discrete TBA macromonomer (*N* < 10) follows a size-dependent exponential decay with *k*_p_ ∼ 1/√*N* (Figure 1e), which is attributed to the self-diffusion coefficient of each macromonomer species in the solution and the steric hindrance of the propagating brush ends.

**Figure 2:**
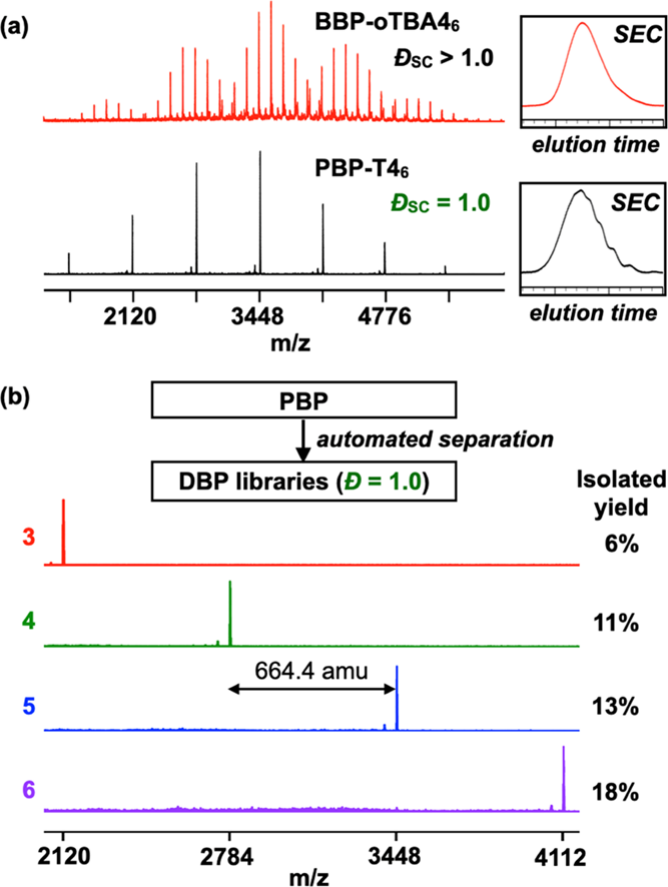
(a) MALDI-ToF spectra and SEC traces of bottlebrush polymers prepared from grafting-through ROMP of disperse tetramer (red) and discrete tetramer (black). (b) MALDI-ToF spectra of discrete bottlebrush polymers (**DBP-T4***_**n**_*, *n* = 3 to 6) with isolated yields.

Importantly, further analysis shows that macromonomer dispersity and size-dependent kinetics significantly amplify the heterogeneity of conventional BBPs because the propagating brush ends encounter different ratios of shorter and longer macromonomers over time. MALDI-ToF analysis of **BBP-oTBA4**_**6**_ reveals a multimodal distribution of >70 bottlebrush species, despite the low dispersity as measured by SEC analysis (*Đ* = 1.2) (Figure 2a). In contrast, the MALDI-ToF spectra of **PBP-T4**_**6**_ show a monomodal distribution of only seven species, a decrease of one magnitude in sample heterogeneity. To understand the impact of macromonomer dispersity and size-dependent polymerization rate on heterogeneity, we constructed a stochastic polymerization model using the *k*_p_ values for each discrete macromonomer. The use of accurate concentration of each species and their *k*_p_ values from the experimental data (Figure 1b,e) enables the model to reproduce the multimodal heterogeneity observed for BBP sample (Figure S47). The model also shows that the propagating brush ends encounter an increasing hexamer-to-trimer ratio throughout the polymerization (Figure S48). Taken together, our simulation confirmed our hypothesis and yielded two important insights: (a) conventional BBPs have tapered or asymmetric side-chain arrangements and (b) a small structural variation in narrow disperse macromonomers can have a profound effect in amplifying the final bottlebrush distribution. As we show later, indeed, the amplification of structural heterogeneity in bottlebrush polymers impacts their properties.

### Discrete Bottlebrush Polymers

To fully understand the impact of heterogeneity on the properties of brush polymers and advance future simulation models, access to truly discrete bottlebrushes (*Đ* = 1.0) is desirable. The well-defined composition of PBPs enabled the isolation of fully discrete bottlebrush polymers (**DBP**), with single molecular ions observed by MALDI-ToF analysis correlating to each expected structure. We were initially encouraged by the unique SEC profile of **PBP-T4**_**6**_, which shows multiple peaks corresponding to bottlebrushes separated by precisely one tetramer side chain despite having a similar overall dispersity with **BBP-oTBA4**, according to SEC analysis (Figure 2a, see also Figure S31 for other PBP samples).

**Figure 3:**
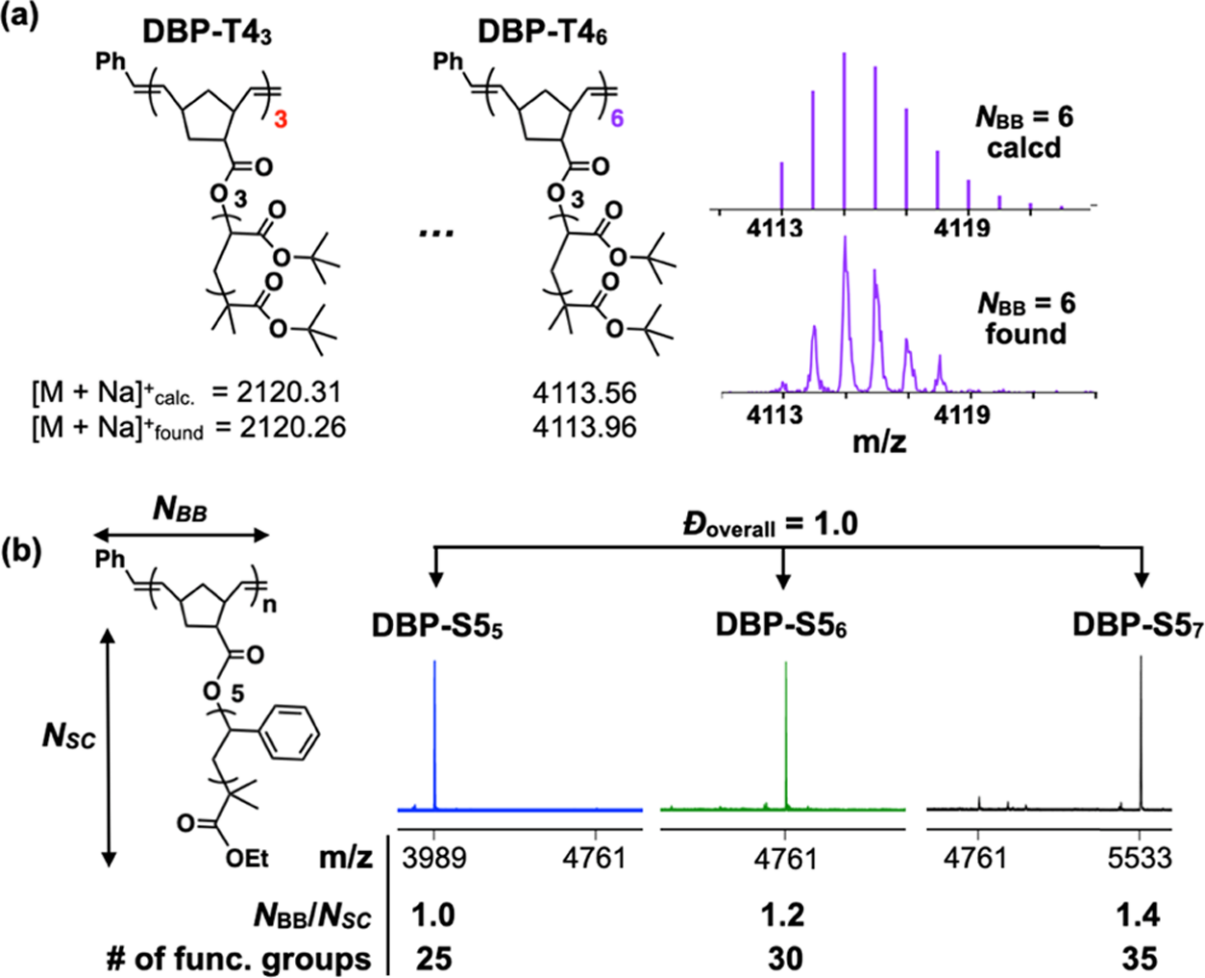
(a) Calculated and observed mass of DBP-T4 species and the isotopic distribution pattern of **DBP-T4**_**6**_. (b) Styrenic **DBP-S5***_**n**_* libraries. Please note that shorter and longer brushes were also obtained.

The clear identification of each PBP species proves useful for optimizing the isolation of a library of **DBPs** in a scalable and consistent manner using recycling preparative SEC or automated flash chromatography (Figures 2b and S30). NMR, SEC, and MALDI-ToF analysis confirmed the isolation of unimolecular brush polymer libraries in near-quantitative yield. The single molecular ion of **DBP-T4**_**6**_ corresponds to the sodium adduct of a bottlebrush polymer with precisely 24 *t*-butyl acrylate functional groups (calculated = 4113.56, observed = 4113.96, Figure 3a). The versatility of this two-step polymerization-separation strategy was demonstrated through the isolation of styrenic-DBPs with a range of molecular weights and aspect ratios such as **DBP-S5**_**5**_ (3,991 Da, *N*_BB_/*N*_SC_ = 1.0), **DBP-S5**_**6**_ (4,763 Da, *N*_BB_/*N*_SC_ = 1.2), and **DBP-S5**_**7**_ (5,536 Da, *N*_BB_/*N*_SC_ = 1.4) (Figures 3b and S30). Note that larger discrete bottlebrushes can be isolated through judicious choice of macromonomer size and separation conditions (e.g., column size, number of cycles). Having homogeneously distributed functional groups and tailorable three-dimensional structures (*N*_BB_/*N*_SC_ ratio), DBP is a new promising addition to the family of precision macromolecules for targeted and precision technologies.

### Impact of Dispersity on Inter-Brush Interactions and Properties

**Figure 4:**
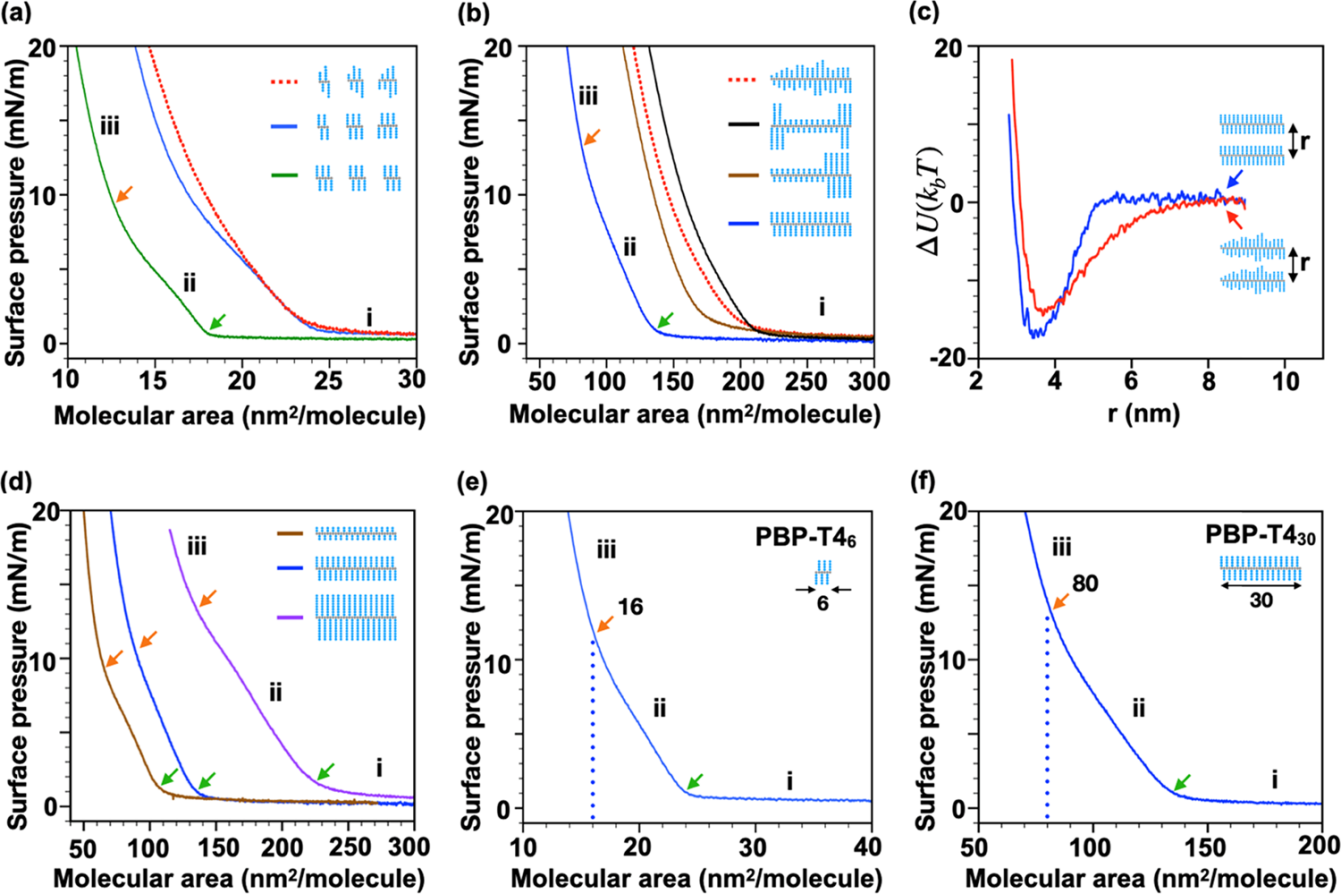
(a) Langmuir–Blodgett (L–B) surface pressure-area isotherms of bottlebrush polymer, **BBP-otBA4**_**6**_ (red), precision bottlebrush polymer, **PBP-T4**_**6**_ (blue), and discrete bottlebrush polymer, **DBP-T4**_**6**_ (green). (b) L–B isotherms of structurally different isomers with *N*_SC_ ≈ 4 and *N*_BB_ ≈ 30; **BBP-otBA4**_**30**_ (dashed red)**, PBP-T4**_**30**_ (blue)**, b-PBP-T2**_**20**_**T8**_**10**_ (brown), and **b-PBP-T8**_**5**_**T2**_**20**_**T8**_**5**_ (black). (c) Interaction potential energy as a function of backbone distance between parallel bottlebrushes, calculated for samples having disperse **BBP-otBA4**_**30**_ (red) and discrete **PBP-T4**_**30**_ (blue) side chains. (d) L–B isotherms of PBPs with *N*_BB_ ≈ 30 with different discrete side chain lengths, **PBP-T2**_**30**_ (brown)**, PBP-T4**_**30**_ (blue), and **PBP-T8**_**30**_ (purple). L–B isotherms of topologically uniform (e) **PBP-T4**_**6**_ and (f) **PBP-T4**_**30**_ with phase transition to close-packed structures labeled. Phase transitions from regime i to ii (gas-to-liquid phase) and ii to iii (liquid-to-solid phase) were determined by taking the first derivative of the isotherms and are indicated with green and orange arrows, respectively. Schematics of bottlebrushes are indicated in the plot legends for clarity.

A question remains regarding the impact of side chain and backbone dispersity and their effect on the properties of bottlebrush polymers. Using a previously established method,^35,51^ we constricted inter-brush interactions to a two-dimensional Langmuir–Blodgett (L–B) monolayer and examined three samples, **BBP-oTBA4**_**6**_ (*Đ*_SC_ = 1.1), **PBP-T4**_**6**_ (*Đ*_SC_ = 1.0,) and **DBP-T4**_**6**_ (*Đ*_overall_ = 1.0). Even for a short *N*_BB_ of 6, only the samples with discrete side chains (PBP and DBP) exhibit previously unseen^51−54^ sharp liquid-to-solid phase transition. Notably, the packing density of brush polymers at the air–water interface increases significantly as the structure becomes fully discrete (Figure 4a). Indeed, the impact of dispersity is important for brush polymers, especially for low *N*_SC_ and *N*_BB_.

Interestingly, the distinct first-order phase transitions from gas-to-liquid and especially liquid-to-solid regimes are also observed for larger PBPs. All PBP samples exhibit this unique behavior regardless of their side chain (*N*_SC_ = 2–8) and average backbone length (*N*_BB_ ≈ 6–30) (Figure 4b,d). In contrast, all disperse side chain samples do not display sharp phase transition from liquid-to-solid regime, presumably due to side-chain dispersity-induced topological defects. We speculate that the uniform topology of PBPs imparts homogeneous backbone stiffness and inter-brush interactions. To understand the impact of side-chain interactions on packing behavior, brush–brush pair potentials were calculated for coarse-grained models of bottlebrushes with disperse and discrete side chains (BBP and PBP, *N*_BB_ ≈ 30, *N*_SC_ ≈ 4). As expected, the disperse side chains of BBP pair interact first at a brush–brush distance of 8 nm gradually, while the side chain–side chain interactions of PBP pair starts at a much shorter distance of 5 nm, followed by a steep potential change to a minimum potential at ∼3.8 nm (Figures 4c and S49). The uniform rigid rod-like behavior of PBP samples and their higher packing density was also confirmed through coarse-grained molecular dynamics studies (Langmuir–Blodgett simulations, Figure S50).

Notably, PBP samples display packing behavior that scales proportionately with their side chain length (Figure S41), which confirms their uniform backbone stiffness and rod-like behavior upon compression at the air–water interface (Figure 4d). Owing to their discrete side chains, the phase transition point from liquid-to-solid for **PBP-T4**_**30**_ is precisely 5 times that of **PBP-T4**_**6**_ (80 vs 16 nm^2^/molecule, Figures 4e–f and S42). Collectively, our results highlight the first observation of both early interactions between bottlebrushes (gas-to-liquid) and the eventual side chain–side chain interaction upon compression (liquid-to-solid), with the overall packing efficiency being strongly impacted by topological uniformity.

### Designing the Side-Chain Topology to Tailor Functions

**Figure 5:**
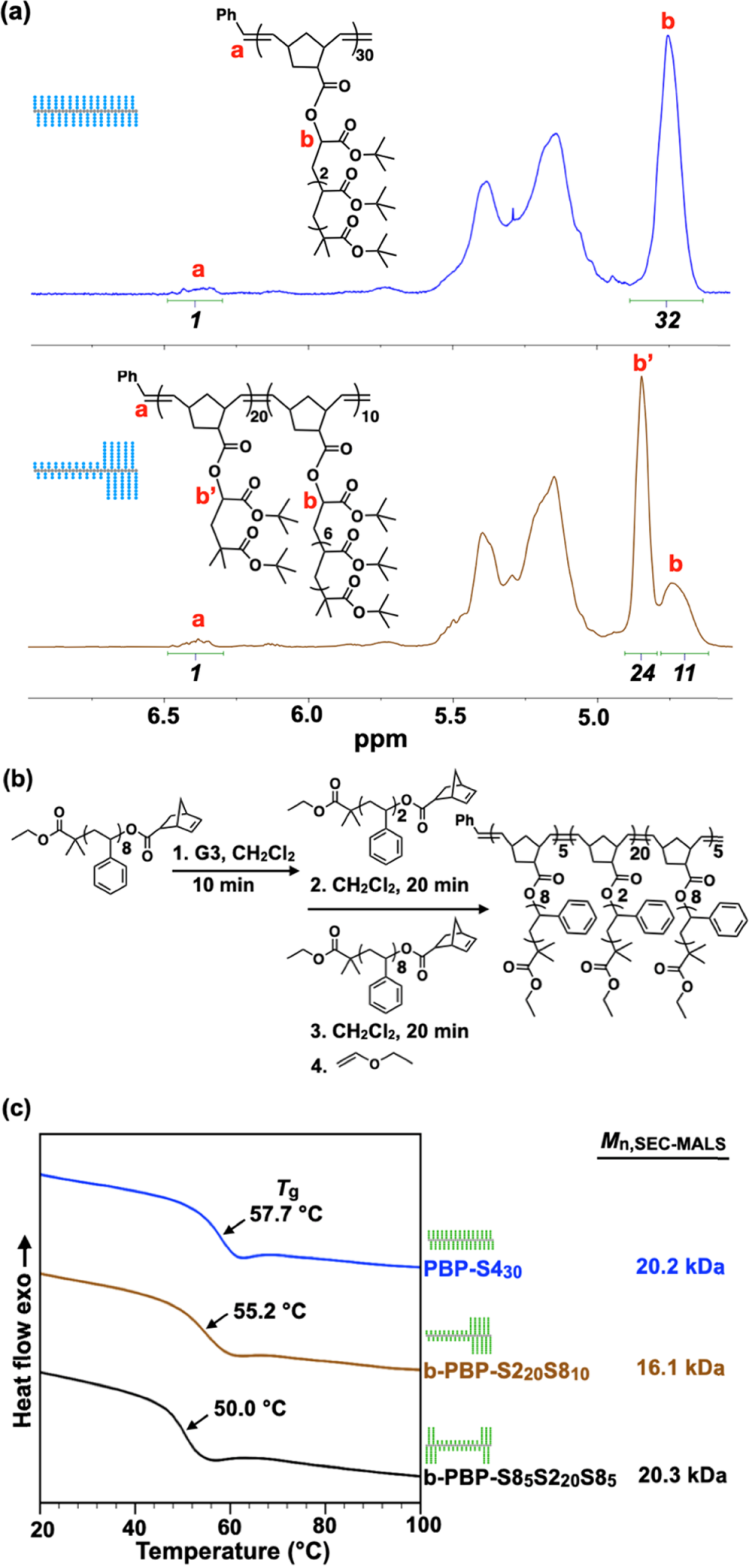
(a) ^1^H NMR spectra of homobrush **PBP-T4**_**30**_ (blue) and diblock **PBP-T2**_**20**_**T8**_**10**_ (brown). All spectra are normalized to the resonance for the chain end protons (a, 6.39 ppm). Integrations of the peaks are shown in italics. (b) Synthesis of styrenic b-PBP (c) DSC traces of homobrush **PBP-S4**_**30**_ (blue), diblock **b-PBP-S2**_**20**_**S8**_**10**_ (brown), and triblock **b-PBP-S8**_**5**_**S2**_**20**_**S8**_**5**_ (black).

The spatial feature of polymers determines their conformational and thermomechanical properties. To illustrate the importance of macromonomer dispersity and designer topology, three architectural variants/isomers were prepared via sequential block polymerization: **PBP-T4**_**30**_ (homo-T4), **b-PBP-T2**_**20**_**T8**_**10**_ (diblock-T2/8), and b-PBP-T8_5_T2_20_T8_5_ (triblock-T8/2/8) (Figures 5a, S12–S15, S32, and S33). Encouragingly, the block size in these b-PBP samples could be estimated using NMR analysis (Figure 5a). L–B studies of these variants clearly demonstrate that a slight change in the side-chain topology dramatically affects the inter-brush interactions of bottlebrush polymers (Figures 4b and S40). Among bottlebrush samples with average *N*_SC_ ≈ 4, the surface pressure (Π) increase is observed first for the triblock-T8/2/8 at 210 molecule/nm^2^, followed by the diblock-T2/8 (170 molecule/nm^2^) and homo-T4 (135 molecule/nm^2^). The significantly lower packing efficiency of the triblock brushes is attributed to the presence of octamer blocks at both brush ends, which increase voids between the dimer sections, as also confirmed via coarse-grained MD simulations (Figure S51). Furthermore, the onset of Π increase for the triblock-T8/2/8 sample at 210 molecule/nm^2^ (Figure 4b) is comparable to the onset of Π increase for PBP with octamer side chains (**PBP-T8**_**30**_, 225 molecule/nm^2^, Figures 4d and S40), which suggests that homo- and multiblock PBPs can be designed to assemble into monolayers with tailorable void space (Figures S50 and S51).

Importantly, the dispersity and block sequence of side chains impact the glass transition temperature (*T*_g_) of bottlebrush polymers. To study the impact of tailoring side-chain dispersity in a precise manner, we prepared poly(styrene)-based bottlebrush variants/isomers, all of which are glassy solid at ambient temperature: **PBP-S4**_**30**_ (homo-S4), **b-PBP-S2**_**20**_**S8**_**10**_ (diblock-S2/8), and **b-PBP- S8**_**5**_**S2**_**20**_**S8**_**5**_ (triblock-S8/2/8, Figure 5b). Interestingly, differential scanning calorimetry (DSC) analysis showed that the homo-S4 and diblock- S2/8 exhibited similar *T*_g_ (58 and 55 °C, respectively, Figure 5c), despite their markedly different side-chain arrangements, as the diblock sample only contains dimer and octamer blocks. As the octamer block was “split” in half and “placed” at each brush ends, the resulting material (triblock-S8/2/8) was found to have a lower *T*_g_ of 50 °C. We attributed this *T*_g_ difference to two factors inherent to the triblock sample: (a) the diminished contribution to the backbone rigidity, and thus *T*_g_, from the shorter octamer block^55^ and (b) the less efficient packing between triblock brushes. Overall, our results underlined (1) the importance of precision design for bottlebrush polymers and (2) its versatility for tuning their thermomechanical properties, particularly those with short side chains, without introducing a different comonomer chemistry.

## Conclusions

In summary, we describe the synthesis of topologically precise and discrete bottlebrush polymer libraries and highlight the importance of side-chain dispersity. Experimental results support our hypothesis that macromonomer size and dispersity have a substantial impact on polymerization kinetics, ultimately affecting the topology of bottlebrush polymers. Our scalable and efficient strategy to prepare bottlebrush polymers with uniform and tailored topology represents a powerful approach for tuning their physical and thermomechanical properties. The significant potential of side-chain dispersity and topology design is illustrated in striking differences in monolayer phase transitions, packing efficiency, and glass transition temperatures between bottlebrushes having disperse and discrete side chains. New fundamental insights and precision models are indeed crucial for enabling a closer integration between experimental and theoretical studies, especially for designing complex “Tetris-like” soft materials (Tetripols) with small three-dimensional features and precise functions, as we have demonstrated through the first report of topologically precise and fully discrete bottlebrush polymers.

## Experimental Section

### Synthesis of Disperse and Discrete Macromonomers

Disperse oligo(*tert*-butyl acrylate) and oligo(styrene) macromonomers were synthesized following previously reported procedures (see the Supporting Information).^26,35^ Discrete macromonomer libraries were prepared through chromatographic separation of disperse parent materials (see the Supporting Information)

### Synthesis of Bottlebrush Polymers

Bottlebrush homopolymers were synthesized via grafting-through ring-opening metathesis polymerization (ROMP) of macromonomer catalyzed by Grubbs third-generation catalyst. Multiblock samples were synthesized via sequential addition of macromonomers (see the Supporting Information).

### Separation of Disperse Macromonomers and Polymers into Discrete Libraries

Automated flash chromatography of macromonomer and bottlebrush polymer samples were performed using a Biotage Isolera One unit equipped with an evaporative light scattering detector (ELSD, Teledyne ISCO). A normal-phase cartridge (25/50/340 g) and a hexane/ethyl acetate gradient were used to separate acrylate macromonomers and bottlebrush polymers. A reversed-phase C18 cartridge and an acetonitrile/hexane gradient were used to separate styrenic macromonomers and bottlebrush polymers.

High-resolution separation of macromonomer and bottlebrush polymer samples was also performed using a preparative-scale recycling size-exclusion chromatography (rSEC, LaboACE LC-5060, JAIGEL-2HR, and 2.5HR columns). Polymer samples (200–500 mg) were dissolved in ethanol-stabilized chloroform (eluent) and filtered before injection. The separation process was monitored in real time to isolate the desired fractions.

### Mass Spectrometry Analysis

MALDI-ToF-MS spectra for all polymer samples were performed in the positive-reflectron mode (Bruker ultrafleXtreme, flexAnalysis software). Unless otherwise stated, MALDI spectra were acquired using a *trans*-2-[3-(4-*tert*-butylphenyl)-2-methyl-2-propenylidene]malononitrile (DCTB) or 2,5-dihydroxybenzoic acid (DHB) /sodium trifluoroacetate (Na-TFA) as matrix.

### Langmuir–Blodgett Isotherm Measurements

Langmuir–Blodgett monolayer experiment was performed on a trough equipped with two movable PTFE barriers and a platinum Wilhelmy plate probe (Biolin Scientific KSV NIMA). For each experiment, the trough and barriers were thoroughly cleaned with three cycles of DI water–acetone–anhydrous ethanol washing and air-drying. The temperature of the trough was maintained at 30 °C with a circulating bath. The bottlebrush polymer sample was spread on the air–water interface by depositing 70 μL of a 0.03 mg/mL chloroform solution. After the solvent evaporated, the barriers were compressed at a rate of 5 mm/min. For each sample, the isotherm measurement was repeated at least twice, and similar procedures were followed for all bottlebrush samples.

### Glass Transition Temperature Measurements

The glass transition temperature (*T*_g_) of polymer samples was analyzed using differential scanning calorimetry (DSC, TA Instruments Discovery DSC 250 + RCS 120). The samples were loaded in a standard aluminum pan, cooled, and heated at a rate of 10 °C/min for several heating and cooling cycles in the range of 0–120 °C. *T*_g_ values for all samples were determined using TA Universal Analysis software (second heating scan).

## Supporting Information Available

Experimental procedures and characterization data (NMR, MALDI-ToF, SEC, FTIR, L–B) for all samples (PDF); simulated Langmuir–Blodgett style isotherms for disperse, discrete side chain, diblock, and triblock bottlebrush polymers (PDF)
Disperse, discrete, diblock, and triblock (MP4)
