# Supplementary material for: Topologically Precise and Discrete Bottlebrush Polymers: Synthesis, Characterization, and Structure–Property Relationships
Source: JACS Au. 2022 Mar 18;2(4):898–905. doi: 10.1021/jacsau.2c00010 (PMC9088296; doi:10.1021/jacsau.2c00010)
Supplement: Supplementary file 1 — au2c00010_si_001.pdf [file au2c00010_si_001.pdf]

Supporting information

Topologically Precise and Discrete Bottlebrush Polymers: Synthesis, Characterization, and Structure-Property Relationships

Nduka D. Ogbonna,[†] Michael Dearman,[†] Cheng-Ta Cho,[†] Bhuvnesh Bharti,[†] Andrew J. Peters,[‡] Jimmy Lawrence.[†,*]

[†]Department of Chemical Engineering, Louisiana State University, Baton Rouge, Louisiana 70803.

[‡]Department of Chemical Engineering, Louisiana Tech University, Ruston, Louisiana 71272.

*Corresponding author: jimmylawrence@lsu.edu

TABLE OF CONTENTS

EXPERIMENTAL PROCEDURES **4**
General information 4
Synthesis of oligo(*tert*-butyl acrylate) (oTBA) using Cu(0)-RDRP 5
Synthesis of ω-norbornenyl oligo(*tert*-butyl acrylate) (NB-oTBA) 6
Synthesis of oligo(styrene) (oSty) using ATRP 8
Synthesis of ω-norbornenyl oligo(styrene) (NB-oSty) 9
Synthesis of bottlebrush homopolymers (BBP and PBP) 10
Synthesis of bottlebrush block copolymers (b-PBP) 11

CHARACTERIZATION DATA **12**
Figure S1. ^{1}H NMR spectra of disperse oTBA4 12
Figure S2. ^{1}H NMR and ^{13}C NMR spectra of disperse NB-oTBA4 13
Figure S3. ^{1}H NMR and ^{13}C NMR spectra of discrete NB-oTBA2 (T2) 14
Figure S4. ^{1}H NMR and ^{13}C NMR spectra of discrete NB-oTBA4 (T4) 15
Figure S5. ^{1}H NMR and ^{13}C NMR spectra of discrete NB-oTBA8 (T8) 16
Figure S6. ^{1}H NMR spectra of disperse oSty4 and disperse oSty4-OH 17
Figure S7. ^{1}H NMR and ^{13}C NMR spectra of disperse NB-oSty4 18
Figure S8. ^{1}H NMR and ^{13}C NMR spectra of discrete NB-oSty2 (S2) 19
Figure S9. ^{1}H NMR and ^{13}C NMR spectra of discrete NB-oSty4 (S4) 20
Figure S10. ^{1}H NMR and ^{13}C NMR spectra of discrete NB-oSty8 (S8) 21
Table S1. Disperse and discrete macromonomers used in this study 22
Figure S11. ^{1}H NMR spectra of BBP-otBA4$_{30}$ 23
Figure S12. ^{1}H NMR and ^{13}C NMR spectra of PBP-T4$_{30}$ 24
Figure S13. ^{1}H NMR spectra of PBP-T2$_{30}$ and PBP-T8$_{30}$ 25
Figure S14. ^{1}H NMR and ^{13}C NMR spectra of b-PBP-T2$_{20}$T8$_{10}$ 26
Figure S15. ^{1}H NMR spectra of b-PBP-T8$_{5}$T2$_{20}$T8$_{5}$ 27
Figure S16. ^{1}H NMR spectra of BBP-oSty4$_{30}$ 27
Figure S17. ^{1}H NMR and ^{13}C NMR spectra of PBP-S4$_{30}$ 28
Figure S18. ^{1}H NMR spectra of b-PBP-S2$_{20}$S8$_{10}$ and b-PBP-S8$_{5}$S2$_{20}$S8$_{5}$ 29
Figure S19. SEC profile of disperse oTBA4 and NB-oTBA4 30
Figure S20. MALDI-ToF spectra of disperse oTBA4 and NB-oTBA4 30
Figure S21. rSEC trace of disperse NB-oTBA4 separation 31
Figure S22. SEC trace of disperse and discrete NB-oTBA 31
Figure S23. MALDI-ToF spectra of disperse and discrete NB-oTBA 32
Figure S24. SEC trace of disperse and discrete NB-oSty 33
Figure S25. MALDI-ToF spectra of disperse and discrete NB-oSty 34
Figure S26. FTIR spectra of NB-otBA4 and T2-T8 35
Figure S27. FTIR spectra of NB-oSty4 and S2-S8 36
Figure S28. SEC traces monitoring conversion of macromonomers with time 38
Figure S29. ^{1}H NMR spectra of kinetics aliquots 39
Table S2. Propagation rate of discrete oTBA macromonomers 40
Figure S30. MALDI-ToF spectra of discrete bottlebrushes from rSEC and flash chromatography 41
Figure S31. SEC traces of BBP-oTBA4$_{6}$, PBP-T4$_{6}$, and DBP-T4$_{6}$ 44
Figure S32. SEC traces of BBP-oTBA4$_{30}$, PBP-T2$_{30}$, PBP-T4$_{30}$, and PBP-T8$_{30}$ 45
Figure S33. SEC traces of diblock b-PBP-T2$_{20}$T8$_{10}$ and triblock b-PBP-T8$_{5}$T2$_{20}$T8$_{5}$ 46
Figure S34. SEC traces of BBP-oSty430, PBP-S4$_{30}$, b-PBP-S2$_{20}$S8$_{10}$, and b-PBP- S8$_{5}$S2$_{20}$S8$_{5}$ 47
Figure S35. FTIR spectra of BBP-oTBA4$_{6}$, PBP-T4$_{6}$, and DBP-T4$_{6}$ 48
Figure S36. FTIR spectra of BBP-oTBA4$_{30}$, PBP-T2$_{30}$, PBP-T4$_{30}$, and PBP-T8$_{30}$ 49
Figure S37. FTIR spectra of diblock b-PBP-T2$_{20}$T8$_{10}$ and triblock b-PBP-T8$_{5}$T2$_{20}$T8$_{5}$ 50
Figure S38. FTIR spectra of BBP-oSty4$_{30}$, PBP-S4$_{30}$, and b-PBP-S2$_{20}$S8$_{10}$ 51
Table S3. Bottlebrush polymers synthesized and used in this study 52
Figure S39. Langmuir-Blodgett (L-B) isotherms of PBP-T4$_{30}$ and PBP-S4$_{30}$ 54
Figure S40. L-B isotherms of BBP-oTBA4$_{30}$, PBP-T4$_{30}$, b-PBP-T2$_{20}$T8$_{10}$, and b-PBP-T8$_{5}$T2$_{20}$T8$_{5}$ 55

Figure S41. L-B surface pressure isotherms of PBP-$T2_{30}$, PBP-$T4_{30}$, and PBP-$T8_{30}$ 55
Figure S42. L-B isotherms of PBP-$T4_{6}$ and PBP-$T4_{30}$ with first derivative inset 56
Figure S43. Glass transition temperature (T_g) of macromonomers NB-oSty4 and S2-S8 56
Figure S44. DSC traces of NB-oSty4 and S2-S8 57
Figure S45. DSC traces of PBP-$S4_{30}$, b-PBP-$S2_{20}S8_{10}$, and b-PBP-$S8_{5}S2_{20}S8_{5}$ 58
Figure S46. Differential DSC traces of PBP-$S4_{30}$, b-PBP-$S2_{20}S8_{10}$, and b-PBP-$S8_{5}S2_{20}S8_{5}$ 59

MODELING **60**
Figure S47. Simulated MALDI-ToF spectra 60
Figure S48. Concentration ratio of side chains along NBB for simulated bottlebrushes 61
Figure S49. Coarse-grain model for the interbrush pair potential calculation 62
Figure S50. Simulation snapshots of L-B monolayer packing for BBP-4_{30} and PBP-4_{30} 63
Figure S51. Simulation snapshots of L-B monolayer packing for PBP-$8_{5}2_{20}8_{5}$ 64
Figure S52. Simulated L-Blodgett pressure isotherm for BBP-4_{30}, PBP-4_{30}, and PBP-$8_{5}2_{20}8_{5}$ 64
Figure S53. Simulated persistence length for BBP-4_{30}, PBP-4_{30}, and PBP-$8_{5}2_{20}8_{5}$ 65

Scheme S1. Comparison of the synthesis of conventional and discrete bottlebrush polymers 66

EXPERIMENTAL PROCEDURES

General information

All reagents were purchased from Sigma-Aldrich/Millipore and used without further purification unless stated otherwise. Copper (II) bromide was purchased from Acros Organics. *tert*-Butyl acrylate and styrene (Alfa Aesar, 99%) were passed through a plug of basic alumina to remove inhibitors prior to use. Solvents for chromatographic processes were purchased from VWR chemicals and used without further purification. Deuterated solvents were purchased from Cambridge Isotope Laboratories.

All reactions were carried out in oven-dried glassware under an inert atmosphere. Reactions were monitored with analytical thin-layer chromatography (TLC) using silica gel 60G F_{254} TLC plates from EMD Millipore and visualized using 254 nm UV, bromocresol green, or potassium permanganate. Automated flash chromatography of macromonomer and bottlebrush polymer samples were performed using a Biotage Isolera One unit equipped with an evaporative light scattering detector (ELSD, Teledyne ISCO). A normal-phase Biotage KP-SIL SNAP/SNAP Ultra cartridge series (25 g/50 g/340 g), and a hexane/ethyl acetate gradient were used to separate acylate macromonomers and bottlebrush polymers. A reversed-phase C18 cartridge and an acetonitrile/hexane gradient were used to separate styrenic macromonomers and bottlebrush polymers. High-resolution separation of macromonomer and bottlebrush polymer samples was also performed using a preparative-scale recycling size exclusion chromatography (rSEC, LaboACE LC-5060, JAIGEL-2HR, and 2.5HR columns). Polymer samples (200 – 500 mg) were dissolved in ethanol-stabilized chloroform (eluent) and filtered before injection. The separation process was monitored in real-time to isolate the desired fractions.

Gel permeation chromatography (GPC) was performed on a TOSOH HLC-8320GPC equipped with a TSKgel superH5000 column (3 µm particle and 20 nm pore size) eluting with tetrahydrofuran (THF). Absolute molecular weight analysis was performed using a Wyatt Dawn EOS multi-angle light scattering (MALS) detector (λ = 658 nm, Astra 6 software). Weight-averaged molecular weights (M_w) and number-averaged molecular weights (M_n) were calculated relative to linear polystyrene standards unless otherwise stated. ^{1}H and ^{13}C Nuclear magnetic resonance (NMR) spectra were recorded on Bruker Avance III 400 and 500 MHz spectrometers at 298 K. Chemical shifts (δ) are given in parts per million (ppm) and referenced using a deuterated solvent signal. MALDI-ToF-MS spectra for all polymer samples were performed in the positive-reflectron mode (Bruker UltrafleXtreme, FlexAnalysis

software). Unless otherwise stated, MALDI spectra were acquired using a *trans*-2-[3-(4-*tert*-butylphenyl)-2-methyl-2-propenylidene]malononitrile (DCTB) or 2,5-dihydroxybenzoic acid (DHB) /sodium trifluoroacetate (Na-TFA) as matrix. FT-IR spectra were collected on a Bruker Tensor 27 system equipped with a room temperature DTSG detector, a mid-IR source (4000 - 400 cm^{-1}), and a KBr beam splitter. The sample cell was a Pike Miracle single-bounce attenuated total reflectance (ATR) cell equipped with a ZnSe single crystal. The spectral resolution was set to 4 cm^{-1} and 32 scans were taken for each sample. Data were processed using Bruker OPUS software suites.

Langmuir-Blodgett monolayer experiment was performed on a trough equipped with two movable PTFE barriers and a platinum Wilhelmy plate probe (Biolin Scientific KSV NIMA). For each experiment, the trough and barriers were thoroughly cleaned three cycles of DI water-acetone-anhydrous ethanol washing and air drying. The temperature of the trough was maintained at 30 °C with a circulating bath. The bottlebrush polymer sample was spread on the air–water interface by depositing 70 µL of a 0.03 mg/mL chloroform solution. After the solvent evaporated, the barriers were compressed at a rate of 5 mm/min. For each sample, the isotherm measurement was repeated at least twice, and similar procedures were followed for all bottlebrush samples.

The glass transition temperature (T_g) of polymer samples was analyzed using differential scanning calorimetry (DSC, TA Instruments Discovery DSC 250 + RCS 120). The samples were loaded in a standard aluminum pan, cooled, and heated at a rate of 10 °C/min for several heating and cooling cycles in the range of 0 – 120 °C. T_g values for all samples were determined using TA Universal Analysis software (2nd heating scan).

Synthesis of oligo(*tert*-butyl acrylate) using Cu(0)-RDRP

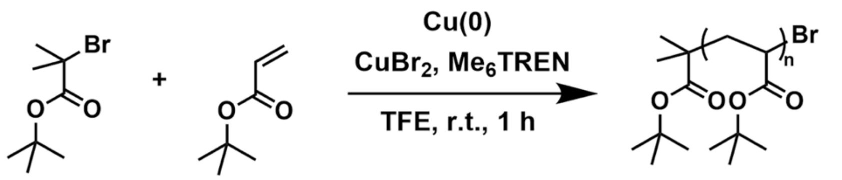

Copper wire (8 cm) was immersed in HCl for 20 min, rinsed with DI water and acetone, and dried. To an oven-dried 40 mL vial, copper(II)bromide ($CuBr_2$) (0.05 equiv.), tris[2-(dimethylamino)ethyl]amine (Me_6TREN) (0.2 equiv.), and trifluoroethanol (TFE) were added, and the contents sonicated for 10 min. *tert*-butyl acrylate (TBA) monomer (n equiv.), *tert*-butyl bromoisobutyrate initiator (TBiB) (1 equiv.), and a stir bar wrapped with the prepared copper wire were added to the vial and degassed with Ar for 20 min. Removing the argon stream, the reaction

proceeded at room temperature while stirring until completion. Reaction progress was monitored via NMR analysis of samples taken at predetermined intervals. The polymerization was terminated by quenching in liquid nitrogen, opening to the atmosphere, and diluting with chloroform. The catalyst was removed from the reaction mixture by passing through a plug of neutral alumina, and excess solvent was reduced in vacuo to obtain *tert*-butyl acrylate oligomers (92% yield).

^{1}H NMR (400 MHz, $CDCl_3$) δ ppm 4.20 – 4.03 (m, 1H), 2.62 – 1.55 (m, 2H 6H), 1.50 – 1.41 (m, 36H), 1.15 – 1.08 (m, 6H).

Synthesis of ω-norbornenyl oligo(*tert*-butyl acrylate) macromonomer

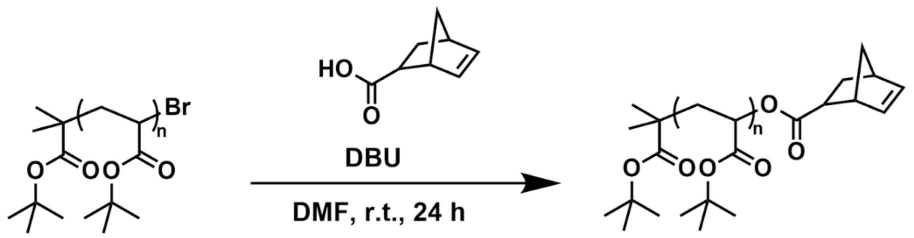

Disperse NB-oTBA4

To an oven-dried 40 mL vial containing oTBA4 (1000 mg, 1.65 mmol), dimethylformamide (DMF, 10 mL) was added to dissolve the sample. *Exo*-5-Norbornene-2-carboxylic acid (455 mg, 3.3 mmol, 2 equiv) was added to the vial followed by dropwise addition of 1,8-diazabicyclo[5.4.0]undec-7-ene (DBU, 492 μL, 3.3 mmol, 2 equiv). The reaction proceeded at room temperature while stirring for 24 h and reaction progress was monitored via thin-layer chromatography. Upon completion of the reaction, the mixture was washed with DI water (40 mL) and ethyl acetate (EtOAc, 40 mL). The organic layer was collected, and the aqueous layer was extracted (3×) with DI water. The combined organic extract was washed with brine (3×) and dried over sodium sulfate (Na_2SO_4). Excess solvent was reduced in vacuo to obtain disperse ω-norbornenyl oligo(*tert*-butyl acrylate) macromonomer. The crude sample was purified using column chromatography, eluting with a hexane/ethyl acetate gradient to give a clear viscous product (990 mg, 90% yield).

^{1}H NMR (400 MHz, $CDCl_3$): δ ppm 6.16 – 6.05 (m, 1H 1H), 4.86 – 4.76 (m, 1H), 3.13 (m, 1H), 2.91 (s, 1H), 2.50 – 2.33 (m, 1H), 2.33 – 2.19 (m, 2H), 2.14 – 1.60 (m, 2H 4H), 1.48 – 1.40 (m, 36H), 1.15 – 1.06 (m, 6H). ^{13}C NMR (125 MHz, $CDCl_3$): δ ppm 176.6, 169.1, 138.1, 135.5, 82.1, 80.1, 70.4, 53.4, 46.5, 41.6, 30.1, 27.9, 23.4. $M_{n,SEC-MALS}$ = 0.7 kDa, $Đ$ = 1.07, $M_{n,NMR}$ = 0.7 kDa. FT-IR (ATR, cm^{-1}): ν = 3100 – 2800 (m; ν_s (sp^3, sp^2 C–H), 1720 (s; ν_s(C=O)), 1150 (s; ν_s (C–O)).

Discrete macromonomers were obtained using either automated flash chromatography or preparative-scale recycling size exclusion chromatography (rSEC).

Discrete NB-oTBA2 (**T2**)

^{1}H NMR (400 MHz, $CDCl_3$) δ ppm 6.17 – 6.04 (m, 2H), 4.95 – 4.87 (dt, J = 10.6 Hz, 1H), 3.13 (dm, J = 28 Hz, 1H), 2.92 (s, 1H), 2.30 – 2.21 (m, 1H), 2.18 – 2.08 (m, 1H), 2.05 – 1.94 (m, 2H), 1.48 (s, 18H), 1.21 – 1.13 (m, 6H). ^{13}C NMR (125 MHz, $CDCl_3$) δ ppm 176.1, 175.4, 169.7, 138.1, 135.6, 81.9, 80.2, 77.3, 70.9, 46.8, 41.6, 40.3, 30.4, 30.0, 25.9.

Discrete NB-oTBA4 (**T4**)

^{1}H NMR (500 MHz, $CDCl_3$) δ ppm 6.16 – 6.03 (m, 2H), 4.87 – 4.78 (m, 1H), 3.13 (m, 1H), 2.92 (s, 1H), 2.50 – 2.34 (m, 1H), 2.36 – 2.20 (m, 2H), 2.16 – 1.60 (m, 2H 4H), 1.50 – 1.39 (m, 36H), 1.12 – 1.05 (m, 6H). ^{13}C NMR (125 MHz, $CDCl_3$) δ ppm 176.6, 173.6, 169.1, 138.2, 135.7, 82.1, 80.0, 76.8, 70.4, 46.5, 41.6, 41.0, 30.1, 27.9, 23.6. MS (MALDI-ToF): $[M+Na]^+$ Calcd for $C_{37}H_{60}O_{10}Na$: 687.41, found: 687.45.

Discrete NB-oTBA8 (**T8**)

^{1}H NMR (400 MHz, $CDCl_3$) δ ppm 6.16 – 6.04 (m, 2H), 4.87 – 4.76 (m, 1H), 3.20 – 3.04 (m, 1H), 2.91 (s, 1H), 2.53 – 2.36 (m, 1H), 2.32 – 1.64 (m, 14H), 1.48 – 1.40 (m, 72H), 1.11 – 1.04 (m, 6H). ^{13}C NMR (125 MHz, $CDCl_3$) δ ppm 176.7, 174.2, 169.0, 138.2, 135.7, 82.1, 80.0, 76.8, 70.4, 46.4, 42.7, 41.7, 30.0, 28.1, 23.8. MS (MALDI-ToF): $[M+Na]^+$ Calcd for $C_{65}H_{108}O_{18}Na$: 1199.74, found: 1199.57.

Synthesis of oligo(styrene) using ATRP

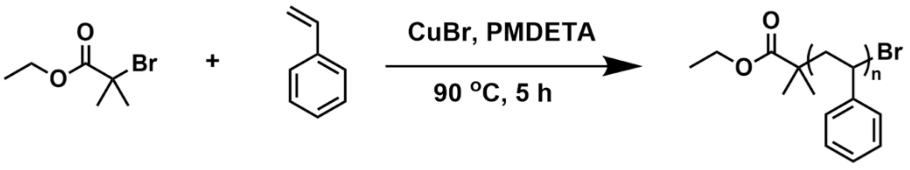

To an oven dried 40 mL vial, copper(I)bromide (CuBr) (0.5 equiv.), N,N,N′,N″,N″-pentamethyldiethylenetriamine (PMDETA) (0.5 equiv.), and styrene (n equiv.) were added and sonicated for 10 min. Ethyl 2-bromoisobutyrate (EBiB) (1 equiv.) and a stir bar were added to the vial and degassed with Ar for 20 min. Removing the argon stream, the reaction proceeded at 90 °C while stirring until completion. Reaction progress was monitored via NMR analysis of samples taken at predetermined intervals. The polymerization was terminated by quenching with liquid nitrogen, exposing to the atmosphere, and diluting it with chloroform. The catalyst was removed from the reaction mixture by passing through a plug of basic alumina, and excess solvent was reduced in vacuo to obtain styrenic oligomers (82% yield).

^{1}H NMR (400 MHz, $CDCl_3$) δ ppm 7.29 – 6.45 (m, 18H), 4.60 – 4.35 (m, 1H), 3.78 – 3.41 (m, 2H), 2.78 – 2.20 (m, 3H), 2.18 – 1.46 (m, 8H), 1.18 – 0.78 (m, 6H 3H).

Synthesis of ω-hydroxyl-terminated oligo(styrene)

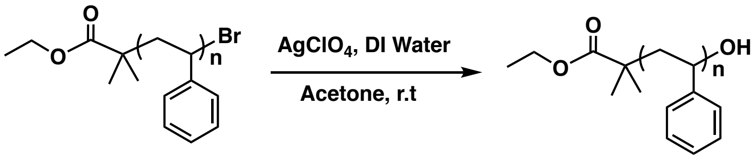

To an oven-dried 40 mL vial, silver perchlorate (815mg, 3.94 mmol) was added and degassed for 20 min. Oligo(styrene) (2000mg, 3.28 mmol) was dissolved in acetone (20 mL) and degassed with Ar for 20 min. Degassed oligo(styrene) solution and excess DI water were added simultaneously to the vial containing silver perchlorate, and the reaction was stirred overnight at room temperature.[1] After completion, the reaction was diluted with THF and passed through a plug of neutral alumina to remove the silver salt. Excess solvent was reduced in vacuo to obtain ω-hydroxyl-terminated oligo(styrene), and the crude sample was purified using column chromatography, eluting with a hexane/ethyl acetate gradient (1410 mg, 78% yield).

^{1}H NMR (400 MHz, $CDCl_3$) δ ppm 7.26 – 6.38 (m, 18H), 4.48 – 4.09 (m, 1H), 3.81 – 3.34 (m, 2H), 2.38 – 1.59 (m, 3H 8H), 1.19 – 0.75 (m, 6H 3H).

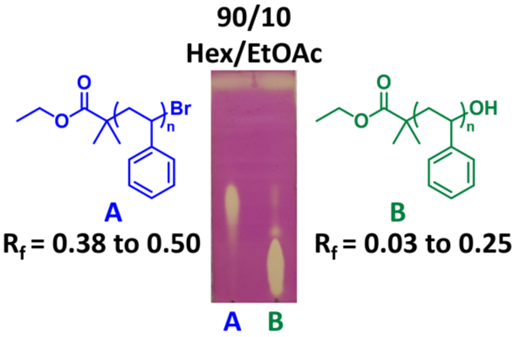

Synthesis of ω-norbornenyl oligo(styrene) macromonomer

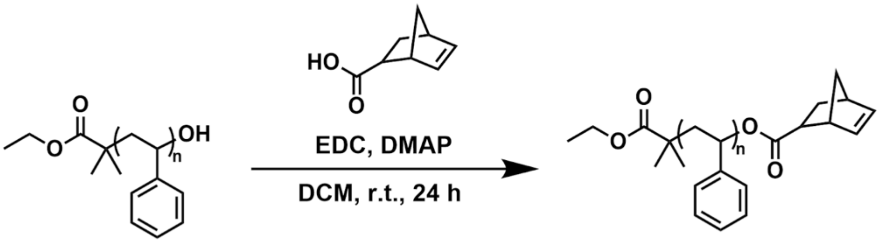

Disperse NB-oSty4

In an oven-dried 40 mL vial, *exo*-5-Norbornene-2-carboxylic acid (378 mg, 2.74 mmol) was dissolved in dichloromethane (DCM, 8 mL). 1-ethyl-3-(3-dimethylamino propyl)carbodiimide (EDC, 525 mg, 2.74 mmol) was added and the content stirred at 0 °C for 15 min. While stirring, ω-hydroxyl-terminated oligo(styrene) (1000 mg, 1.82 mmol) and dimethylaminopyridine (DMAP, 220 mg, 1.82 mmol) were added, and the reaction was brought to room temperature, stirring for ~24 h. Thin-layer chromatography (TLC) was used to monitor the reaction progress. The reaction was terminated by diluting with DCM. Excess solvent was reduced in vacuo to obtain ω-norbornenyl oligo(styrene) macromonomer. The crude sample was purified using column chromatography, eluting with a hexane/ethyl acetate gradient (929 mg, 76% yield).

^{1}H NMR (400 MHz, $CDCl_3$) δ ppm 7.26 – 6.42 (m, 20H), 6.23 – 5.98 (m, 1H 1H), 5.67 – 5.19 (m, 1H), 3.82 – 3.38 (m, 2H), 3.04 – 2.77 (m, 1H 1H), 2.38 – 1.59 (m, 3H 8H), 1.19 – 0.75 (m, 6H 3H). ^{13}C NMR (125 MHz, $CDCl_3$): δ ppm 177.6, 175.0, 145.0, 138.0, 135.8, 128.3, 127.6, 126.0, 77.3, 73.9, 60.0, 46.5, 41.7, 30.0, 26.5, 14.0. $M_{n,MALDI-}$

ToF = 0.6 kDa, $Đ$ = 1.16 , $M_{n,NMR}$ = 0.6 kDa. FT-IR (ATR, cm^{-1}): ν = 3100 – 2800 (m; ν_s (sp^3, sp^2 C–H), 1720 (s;ν_s(C=O)), 1180 (m; ν_s (C-O)), 700 (s; ν_b (sp^2 =C–H)).

Discrete macromonomers were obtained using either automated flash chromatography or preparative-scale recycling size exclusion chromatography (rSEC).

Discrete NB-oSty2 (**S2**)

^{1}H NMR (400 MHz, $CDCl_3$) δ ppm 7.26 - 7.00 (m, 10H), 6.18 – 6.04 (m, 1H 1H), 5.51 – 5.20 (m, 1H), 3.85 – 3.52 (m, 2H), 3.07 (m, 1H), 2.92 (s, 1H), 2.29 – 1.80 (m, 2H 1H 2H), 1.16 – 0.94 (m, 6H 3H). ^{13}C NMR (125 MHz, $CDCl_3$): δ ppm 177.3, 177.8, 143.9, 141.4, 138.1, 135.8, 128.4, 125.9, 73.9, 60.1, 46.7, 41.9, 39.4, 30.4, 26.5, 25.4, 13.9. MS (MALDI-ToF): $[M+Na]^+$ Calcd for $C_{30}H_{36}O_4Na$: 460.26, found: 460.27.

Discrete NB-oSty4 (**S4**)

^{1}H NMR (400 MHz, $CDCl_3$) δ ppm 7.26 – 6.72 (m, 20H), 6.20 – 5.99 (m, 1H 1H), 5.66 – 5.21 (m, 1H), 3.76 – 3.39 (m, 2H), 3.02 – 2.66 (m, 1H 1H), 2.36 – 1.59 (m, 3H 8H), 1.09 – 0.79 (m, 6H 3H). ^{13}C NMR (125 MHz, $CDCl_3$): δ ppm 177.6, 175.0, 145.0, 137.9, 135.8, 128.1, 126.0, 73.8, 46.5, 43.4, 41.9, 40.2, 30.2, 26.5, 13.9. MS (MALDI-ToF): $[M+Na]^+$ Calcd for $C_{46}H_{52}O_4Na$: 668.39, found: 668.46.

Discrete NB-oSty8 (**S8**)

^{1}H NMR (400 MHz, $CDCl_3$) δ ppm 7.25 – 6.36 (m, 40H), 6.19 – 5.98 (m, 1H 1H), 5.61 – 5.17 (m, 1H), 3.72 – 3.32 (m, 2H), 2.99 – 2.63 (m, 1H 1H), 2.54 – 1.53 (m, 7H 16H), 1.06 – 0.79 (m, 6H 3H). ^{13}C NMR (125 MHz, $CDCl_3$): δ ppm 177.5, 174.9, 145.1, 138.1, 135.8, 128.0, 125.7, 73.8, 59.9, 46.3, 43.2, 41.6, 40.5, 30.4, 25.9, 24.9, 13.9. MS (MALDI-ToF): $[M+Na]^+$ Calcd for $C_{78}H_{84}O_4Na$: 1084.64, found: 1084.41.

Synthesis of bottlebrush homopolymers using discrete and disperse macromonomers

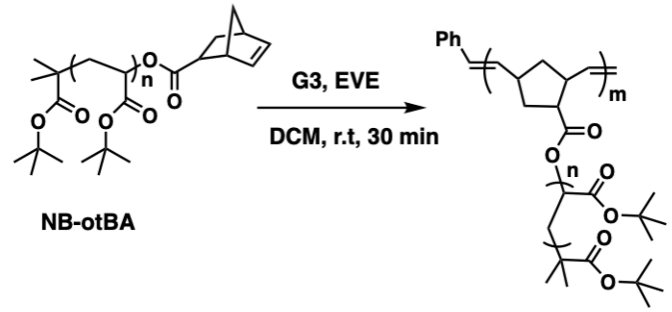

Grubbs 3rd generation catalyst (G3) was prepared following reported procedures,[2] and DCM was degassed with Ar for 30 min prior to use.

To an oven-dried 4 mL vial equipped with a stir bar, NB-otBA macromonomer (m equiv.) was dissolved in DCM and degassed with Ar for 20 min. A degassed solution of Grubbs 3rd generation catalyst $(IMesH_2)(Cl)_2(C_5H_5N)_2Ru{=}CHPh$ (G3, 1 equiv, 100 mM) was injected into the mixture to initiate the polymerization. The mixture was stirred at room temperature for 30 min, and the polymerization was stopped by quenching with excess ethyl vinyl ether (EVE). The crude product was passed through a plug of basic alumina to remove the catalyst, and excess solvent was reduced in vacuo to obtain bottlebrush homopolymers (90% yield).

Synthesis of multiblock bottlebrush polymers.

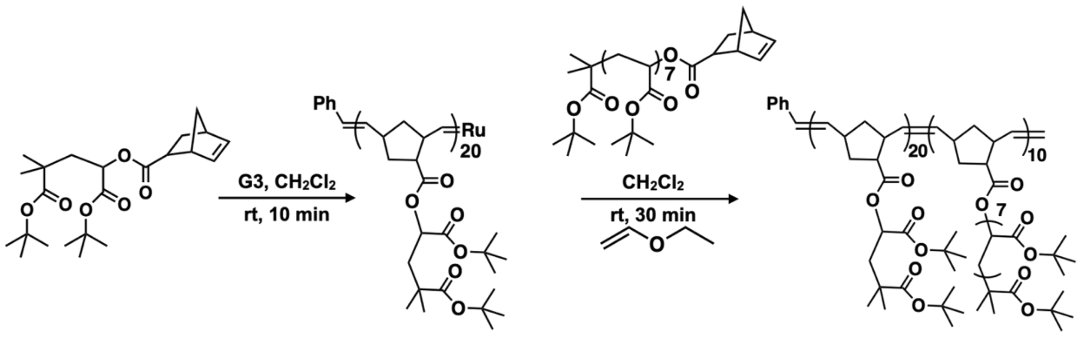

PBP-T2$_{20}$T8$_{10}$

Grubbs 3rd generation catalyst (G3) was prepared following reported procedures,[2] and DCM was degassed with Ar for 30 min prior to use.

To an oven-dried 4 mL vial equipped with a stir bar, **T2** (1st block, 20 equiv.) was dissolved in DCM and degassed with Ar for 20 min. A degassed solution of **G3** (1 equiv., 100 mM) was injected into the mixture to initiate the polymerization. The mixture was stirred at room temperature for 10 min, and an aliquot was collected. Degassed **T8** solution (2nd block, 10 equiv.) was injected into the reaction mixture, and the mixture was stirred for an additional 30 min. The polymerization was stopped by quenching with excess ethyl vinyl ether (EVE). The crude product was passed through a plug of basic alumina to remove the catalyst, and excess solvent was reduced in vacuo to obtain a diblock bottlebrush polymer (90% yield).

CHARACTERIZATION DATA

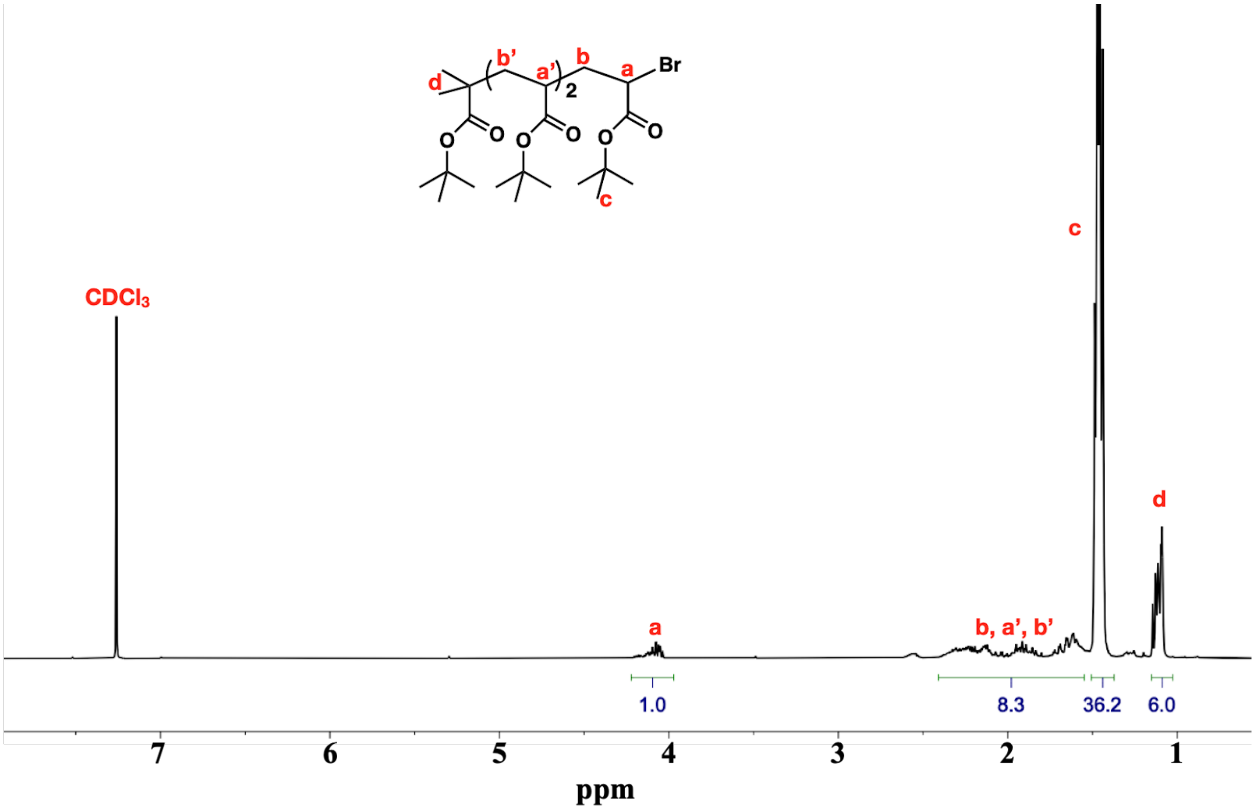

Figure S1. ^{1}H NMR (400 MHz, $CDCl_3$) of disperse **oTBA4** (oligo(*tert*-butyl acrylate), average *DP* = 4)

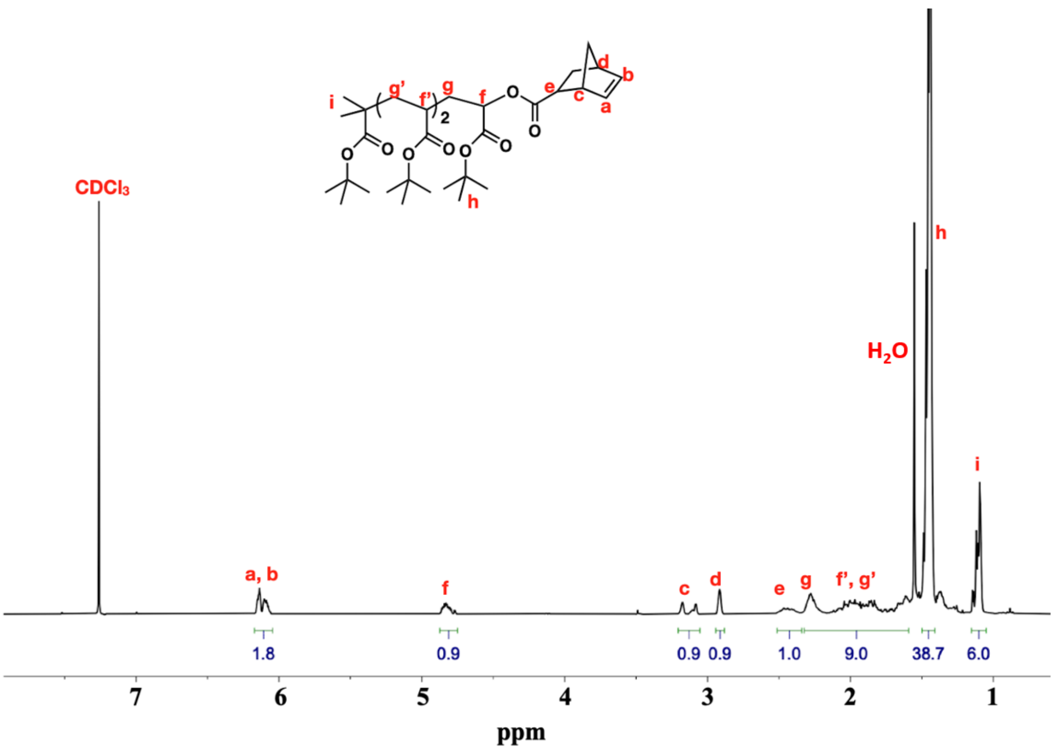

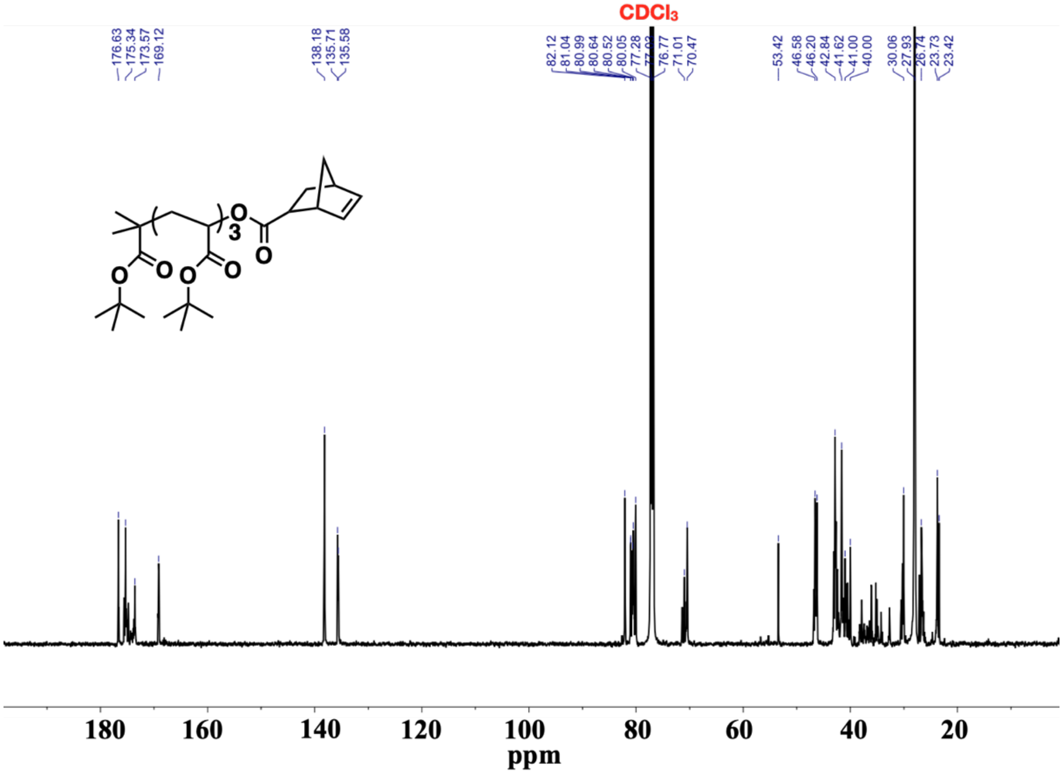

Figure S2. ¹H NMR (400 MHz, $CDCl_3$) and ¹³C NMR (125 MHz, $CDCl_3$) of disperse **NB-oTBA4** (ω-norbornenyl oligo(*tert*-butyl acrylate), average *DP* = 4).

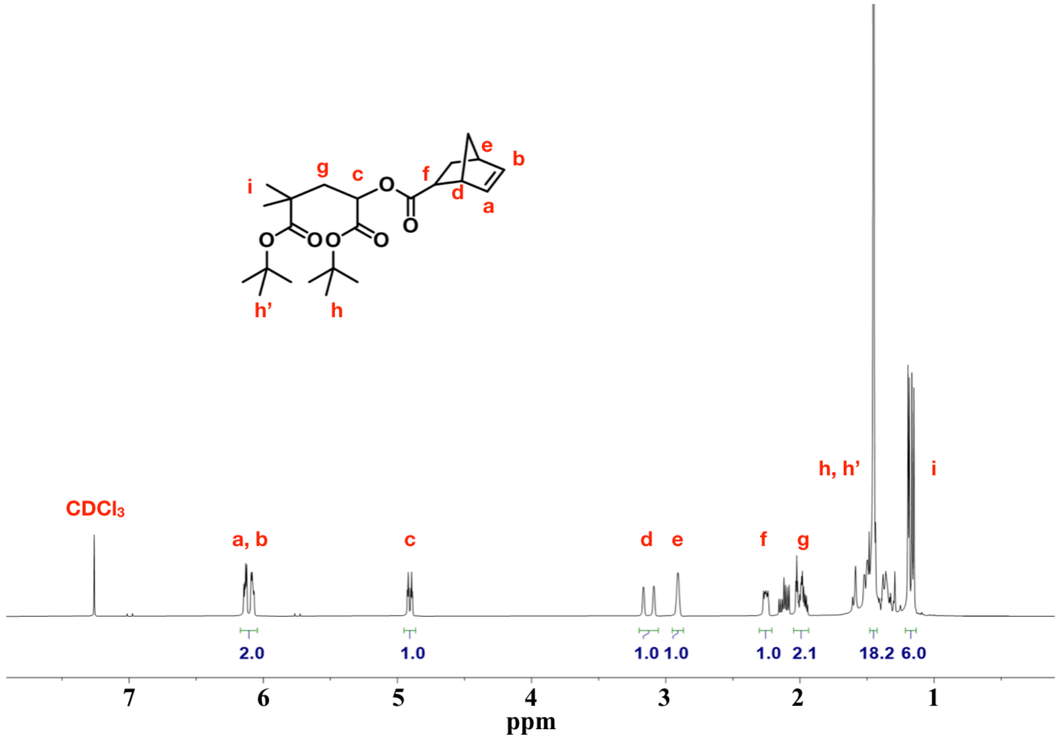

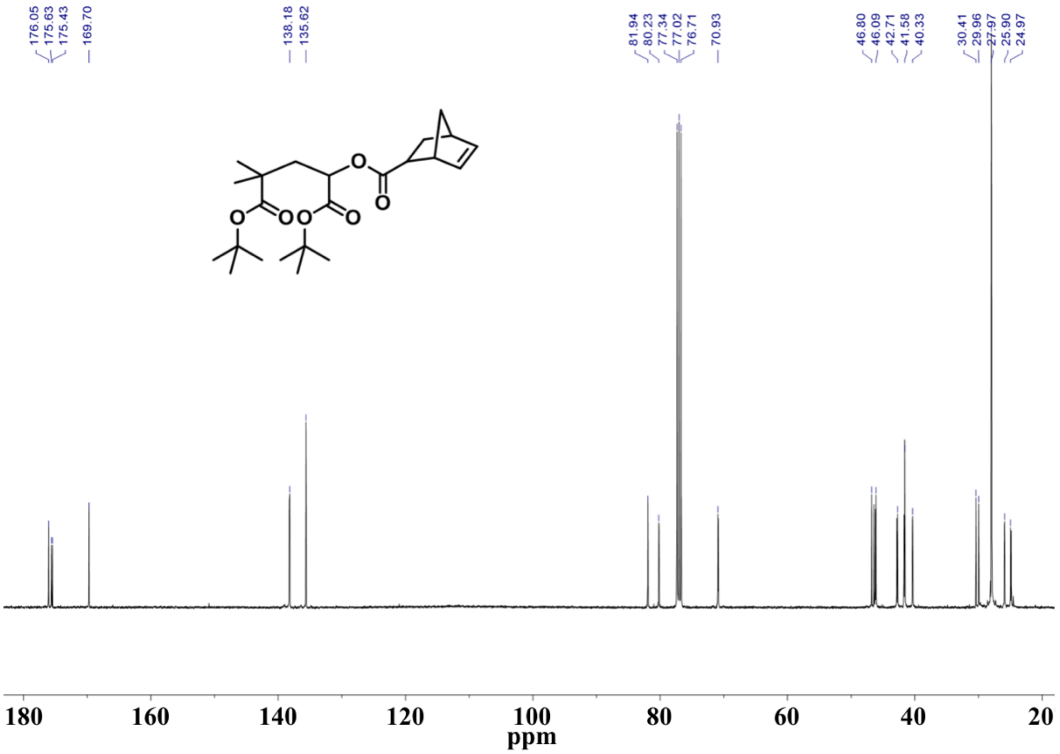

Figure S3. ^{1}H NMR (400 MHz, $CDCl_3$) and ^{13}C NMR (125 MHz, $CDCl_3$) of discrete NB-oTBA2 **(T2)**.

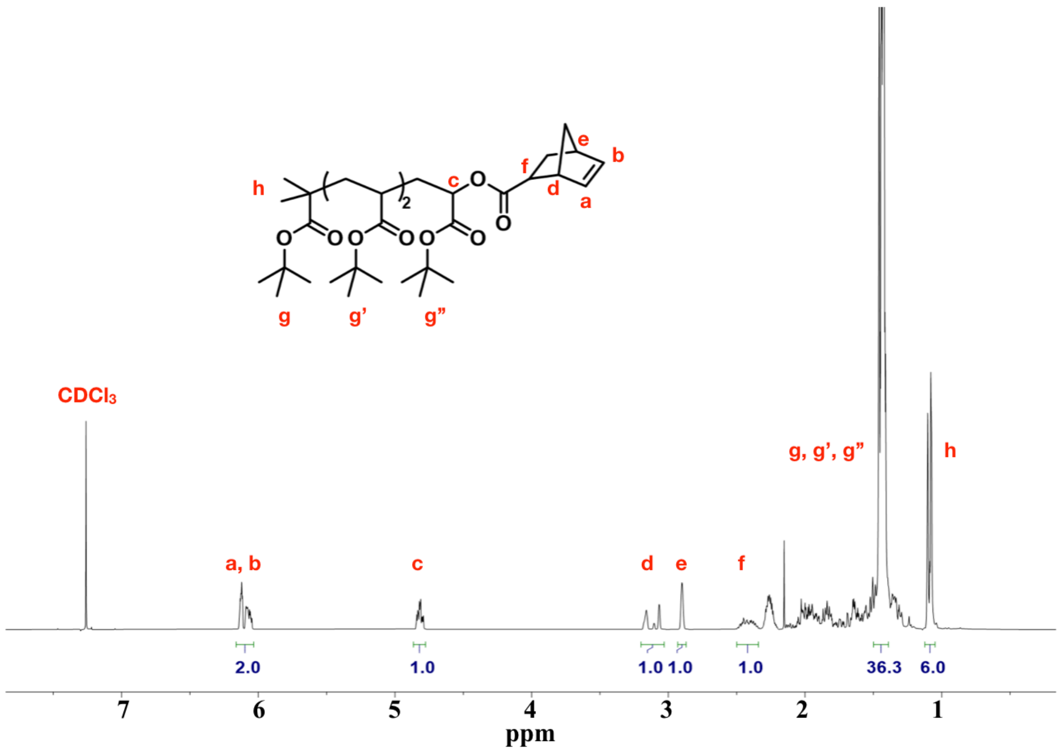

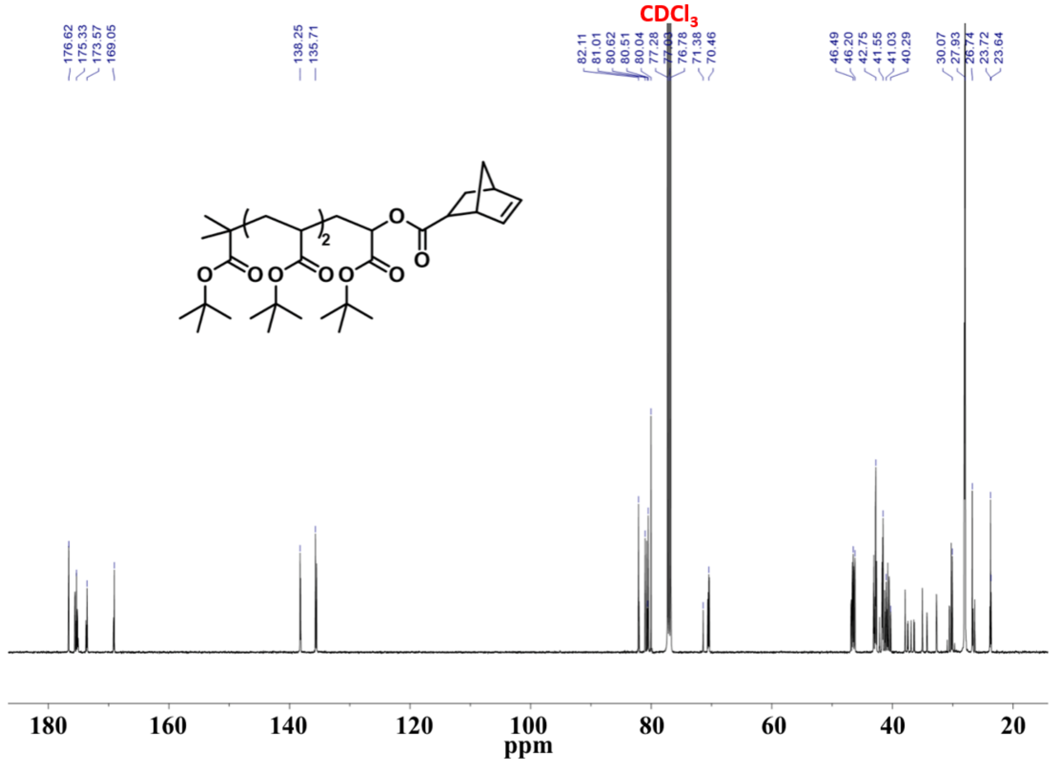

Figure S4. ^{1}H NMR (500 MHz, $CDCl_3$) and ^{13}C NMR (125 MHz, $CDCl_3$) of **T4**.

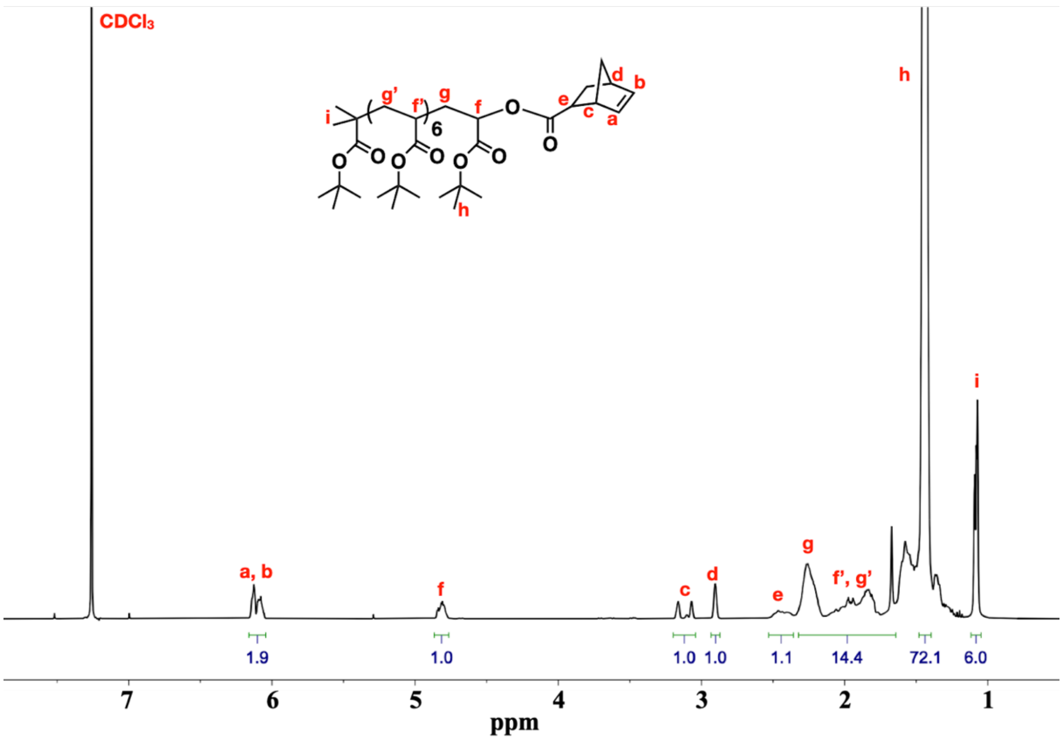

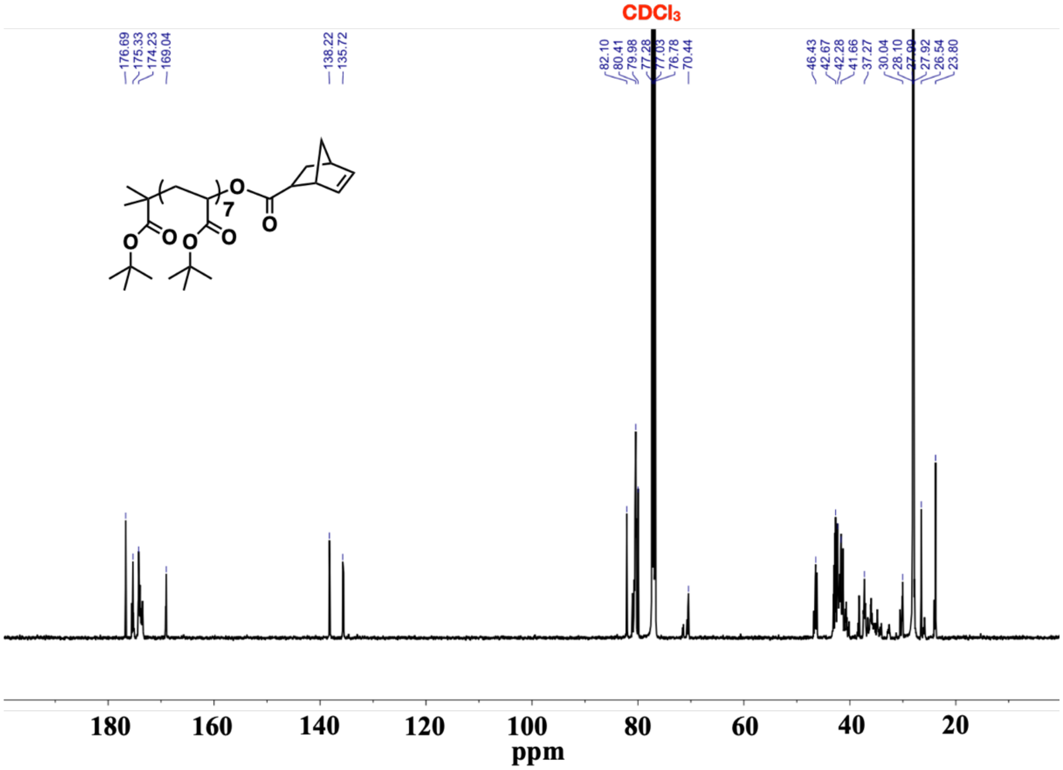

Figure S5. ^{1}H NMR (400 MHz, $CDCl_3$) and ^{13}C NMR (125 MHz, $CDCl_3$) of **T8.**

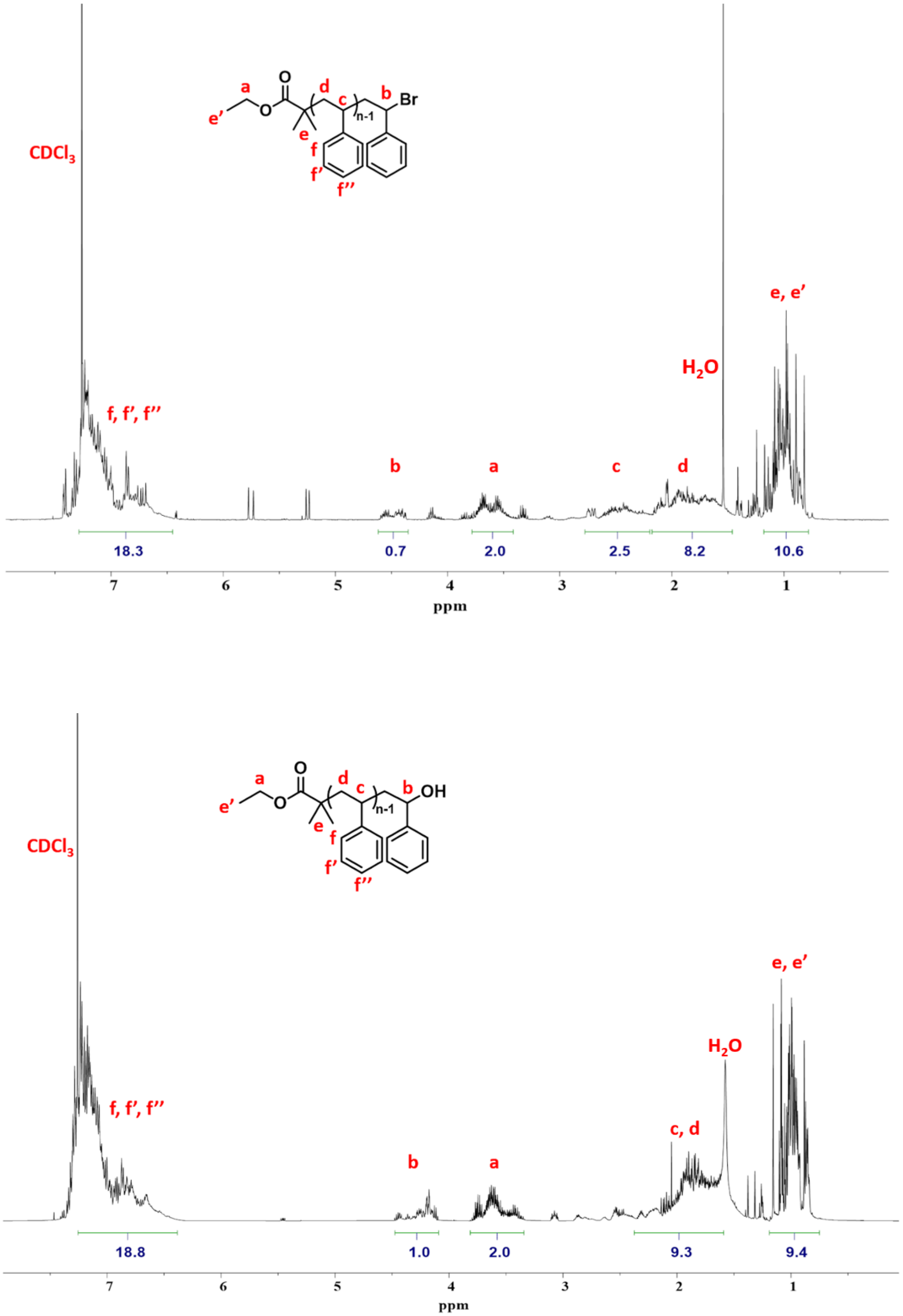

Figure S6. ^{1}H NMR (400 MHz, $CDCl_3$) of disperse **oSty4-Br** and **oSty4-OH** (ω-hydroxyl-terminated oligo(styrene), average *DP* = 4).

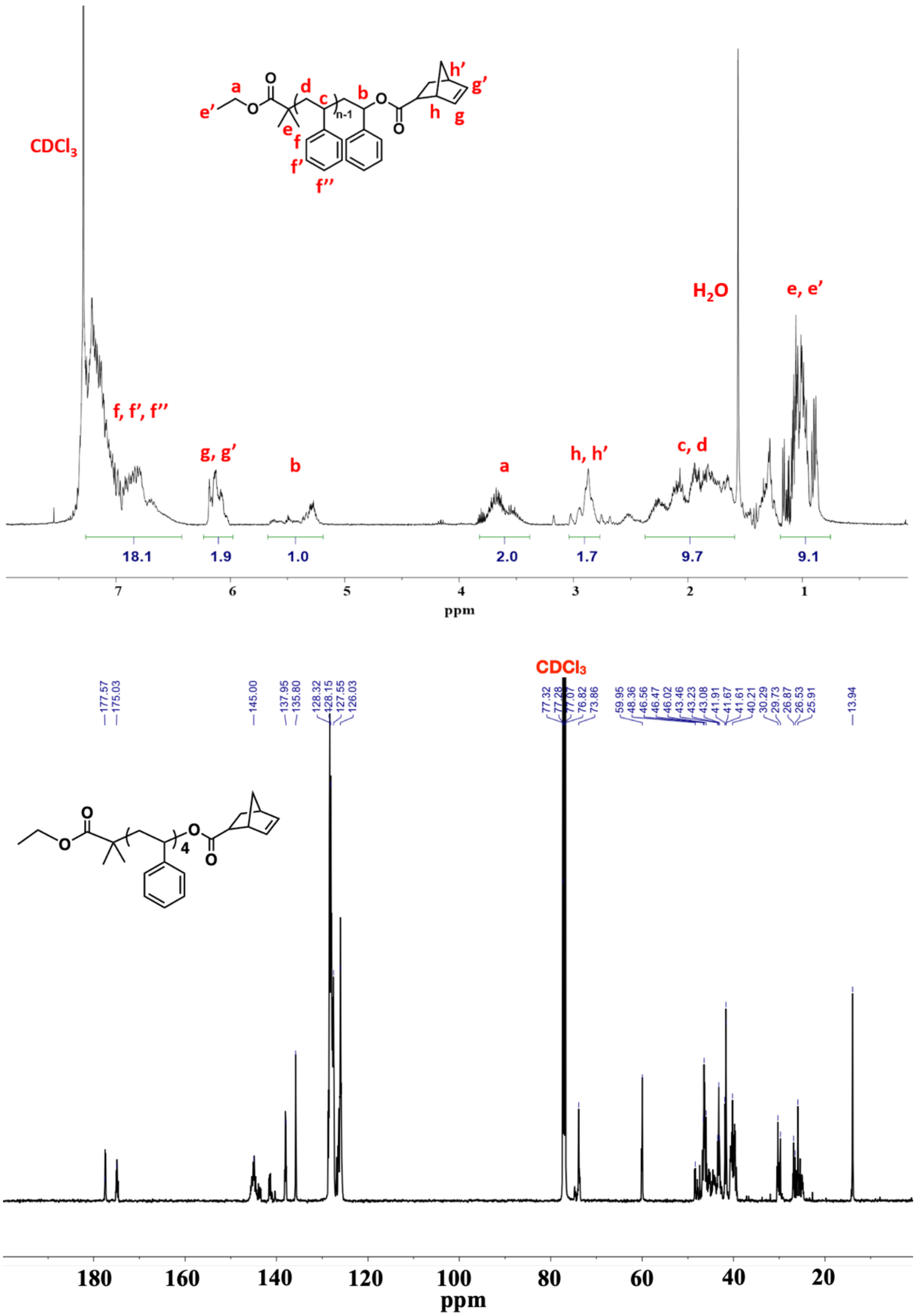

Figure S7. ^{1}H NMR (400 MHz, $CDCl_3$) and ^{13}C NMR (125 MHz, $CDCl_3$) of disperse **NB-oSty4** (ω-norbornenyl oligo(styrene), average *DP* = 4).

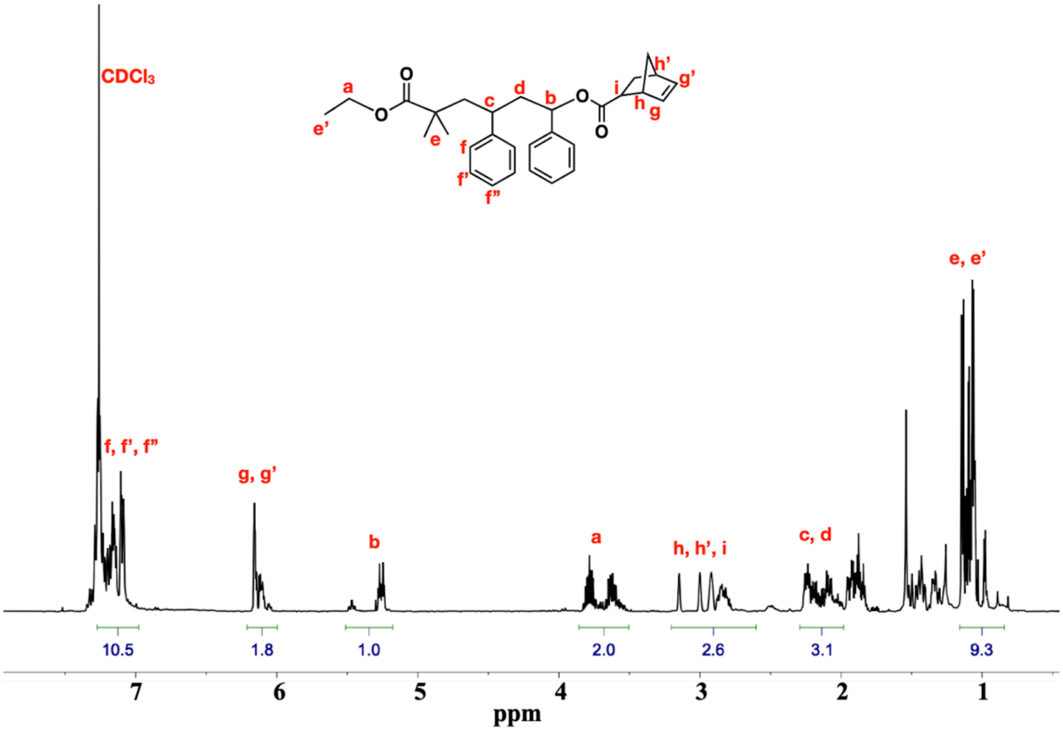

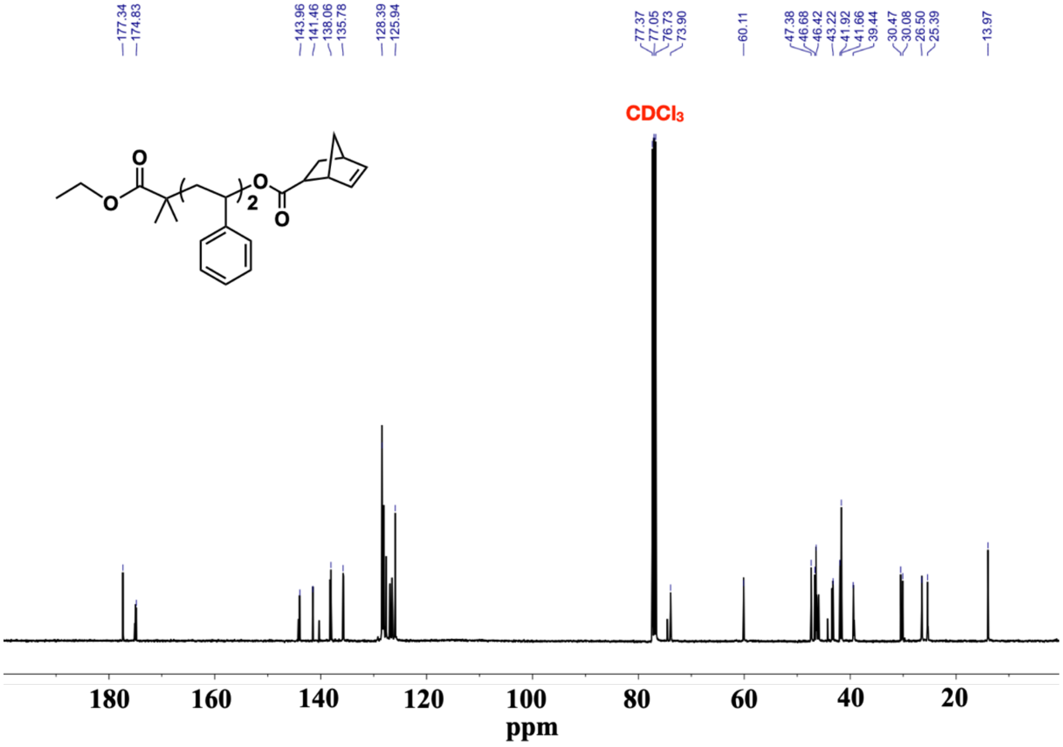

Figure S8. ^{1}H NMR (400 MHz, $CDCl_3$) and ^{13}C NMR (125 MHz, $CDCl_3$) of discrete NB-oSty2 **(S2).**

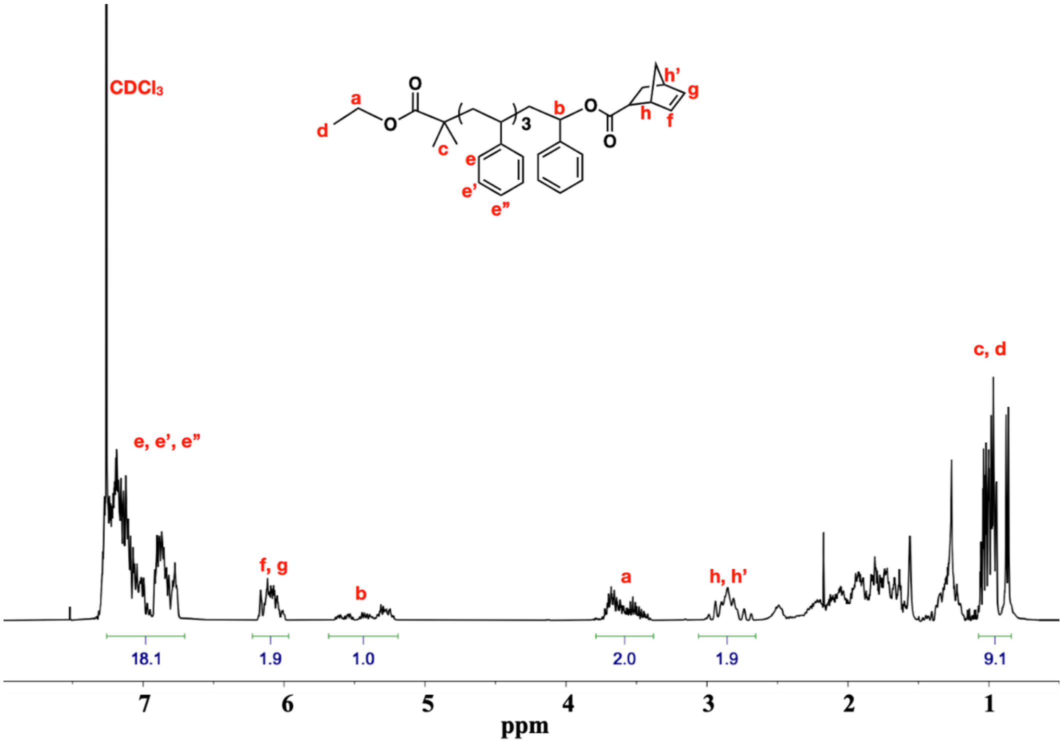

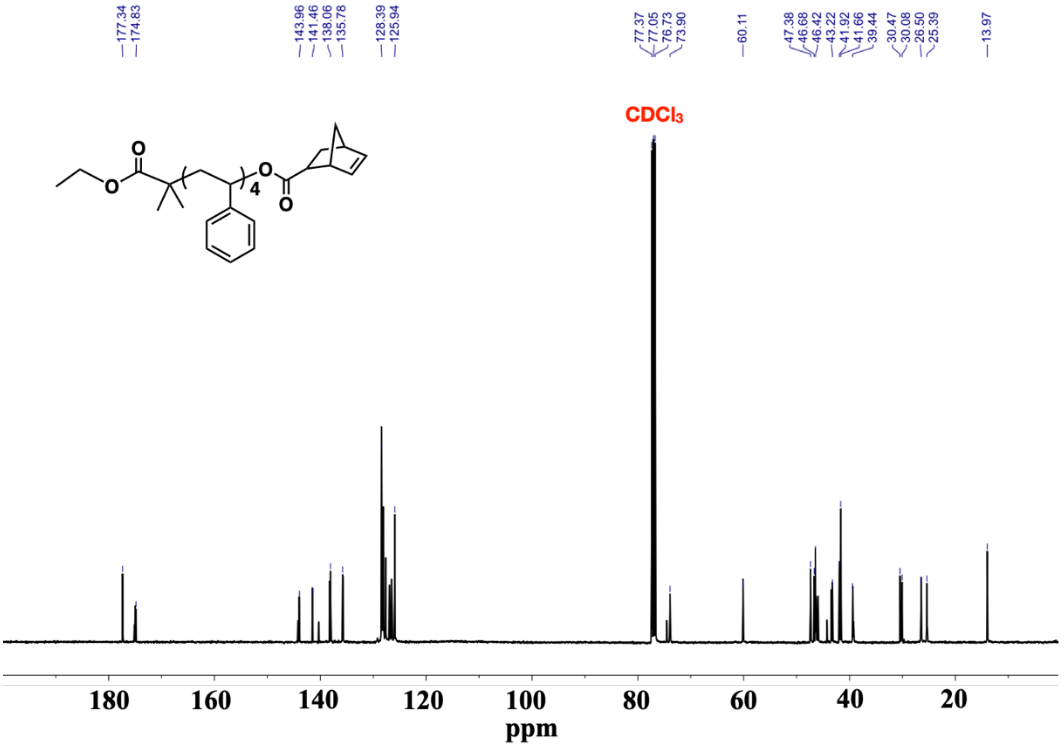

Figure S9. ^{1}H NMR (400 MHz, $CDCl_3$ and ^{13}C NMR (125 MHz, $CDCl_3$) of **S4.**

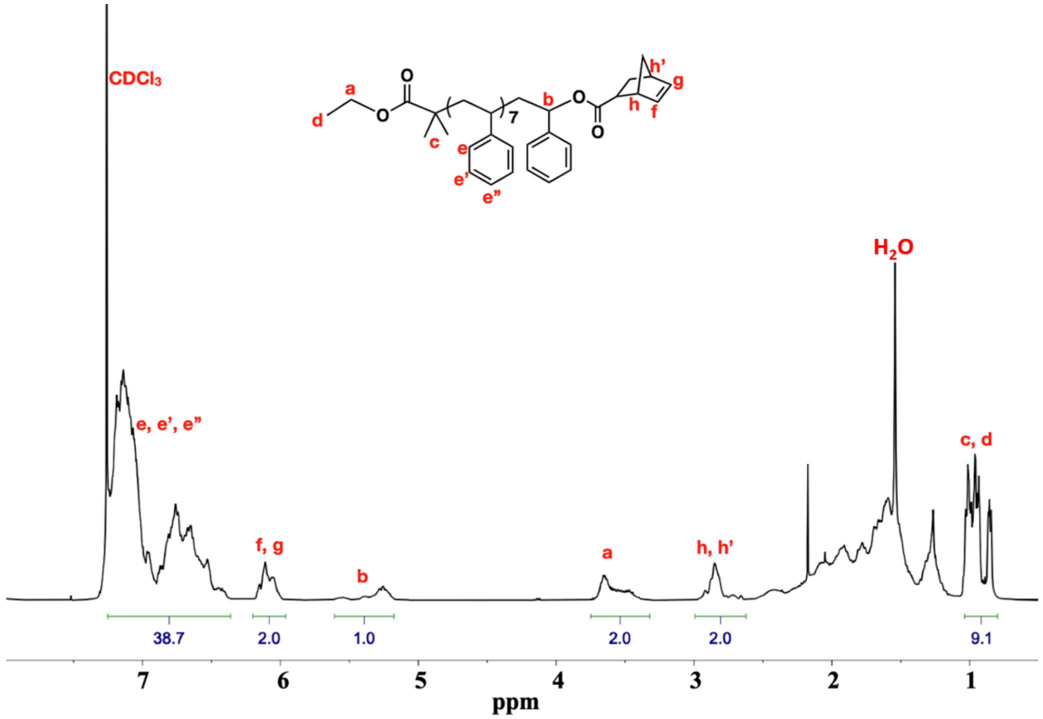

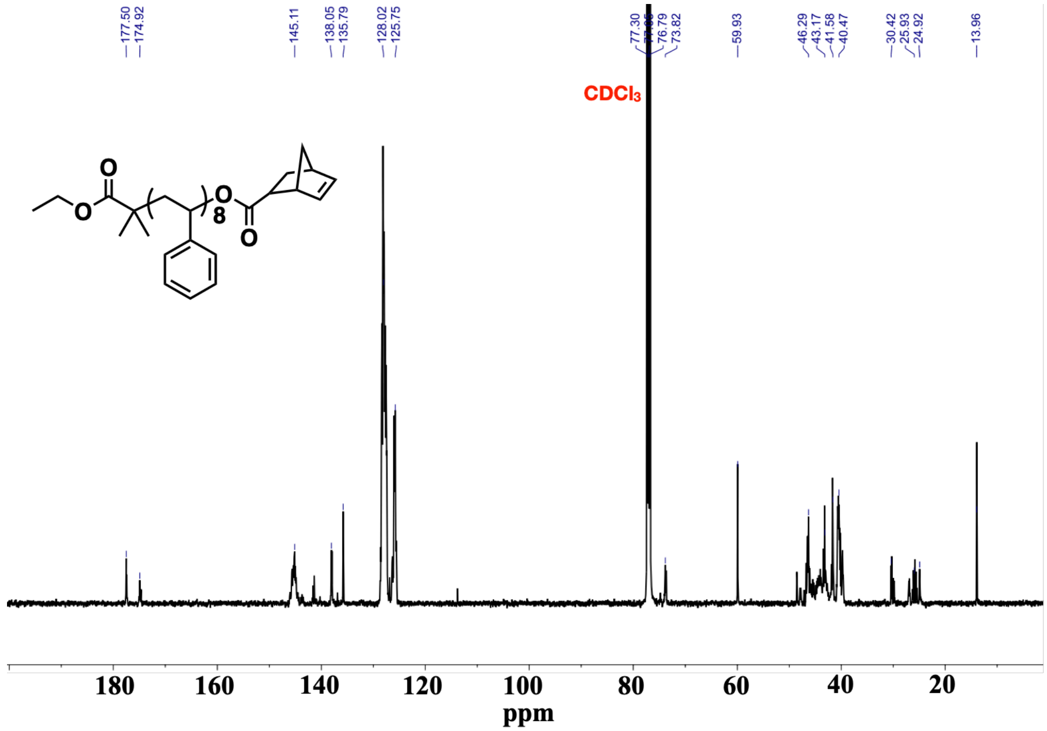

Figure S10. ^{1}H NMR and ^{13}C NMR (400 MHz, $CDCl_3$) of **S8.**

Table S1. Disperse and discrete macromonomers used in this study

A. Oligo (*tert*-butyl acrylate) macromonomer

label	macromonomer[a]	M_n [b] (Da)	$Đ$[b]
NB-otBA4	Disperse NB-oTBA4	~700[c]	1.07[c]
T2	Discrete NB-oTBA2	408.25	1.00
T4	Discrete NB-oTBA4	664.45	1.00
T8	Discrete NB-oTBA8	1176.58	1.00

B. Oligostyrene macromonomer

label	macromonomer[a]	M_n [b] (Da)	$Đ$[b]
NB-oSty4	Disperse NB-oSty4	~630[c]	1.16[c]
S2	Discrete NB-oSty2	460.26	1.00
S4	Discrete NB-oSty4	668.46	1.00
S5	Discrete NB-oSty5	772.45	1.00
S8	Discrete NB-oSty8	1084.41	1.00

[a]The numbers represent the number of *t*-butyl acrylate or styrene repeat units. [b]Determined using MALDI-ToF. [c]Determined using SEC.

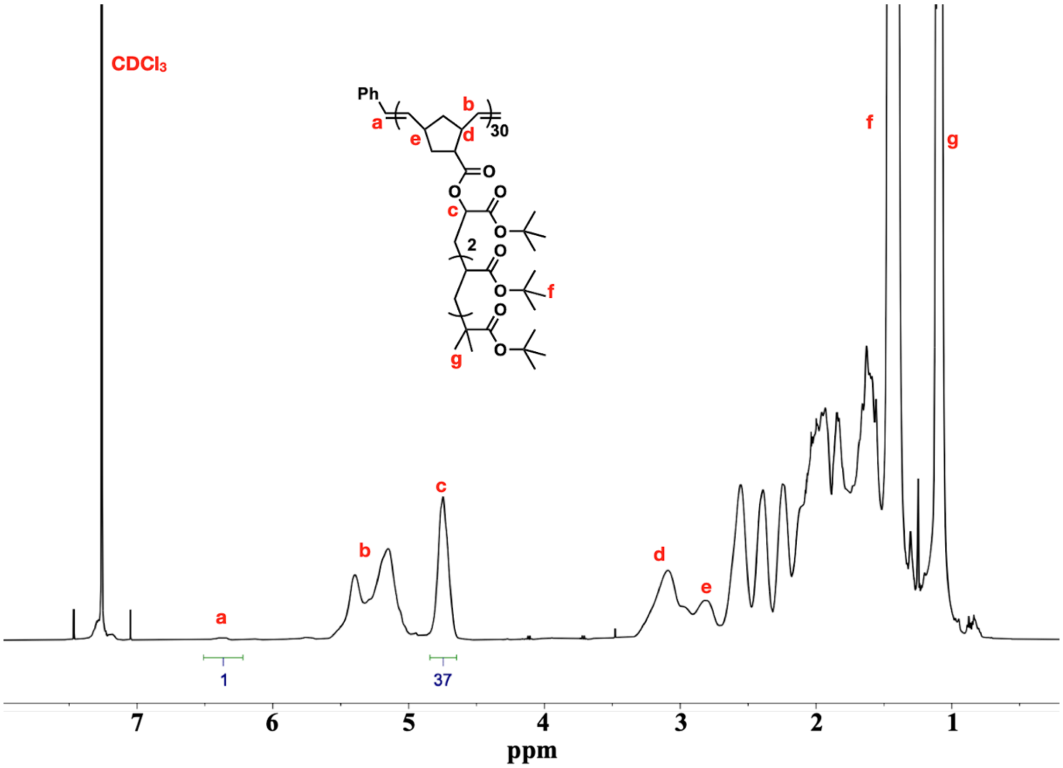

Figure S11. ^{1}H NMR (500 MHz, $CDCl_3$) of **BBP-oTBA4$_{30}$.**

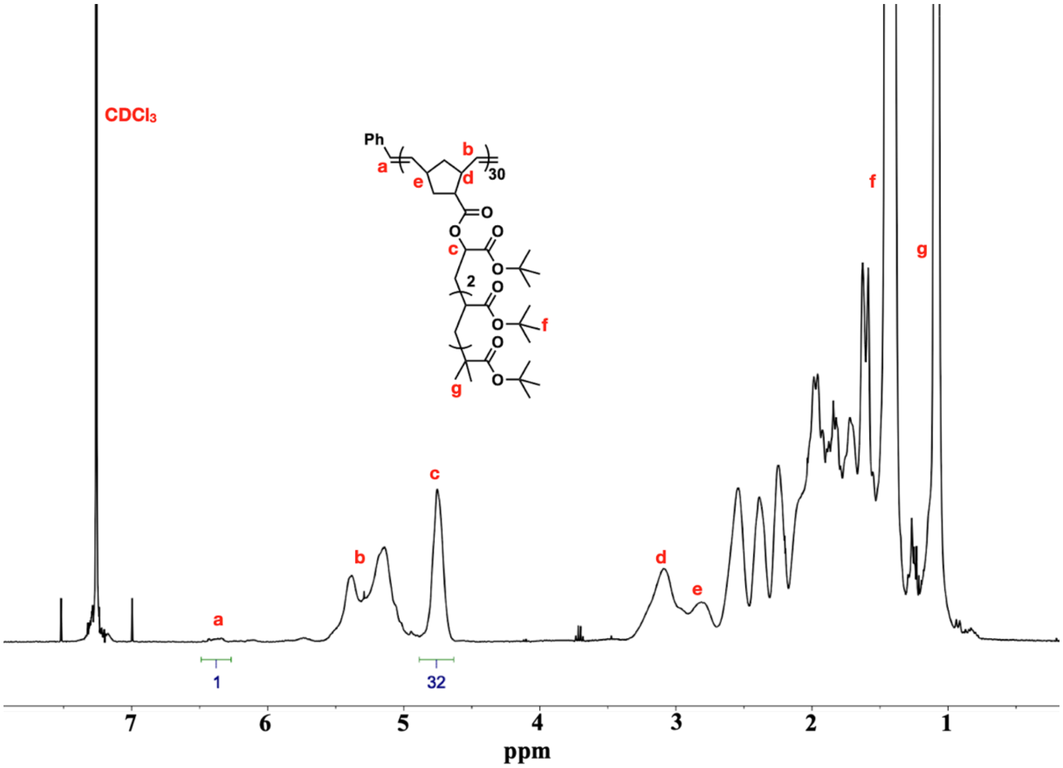

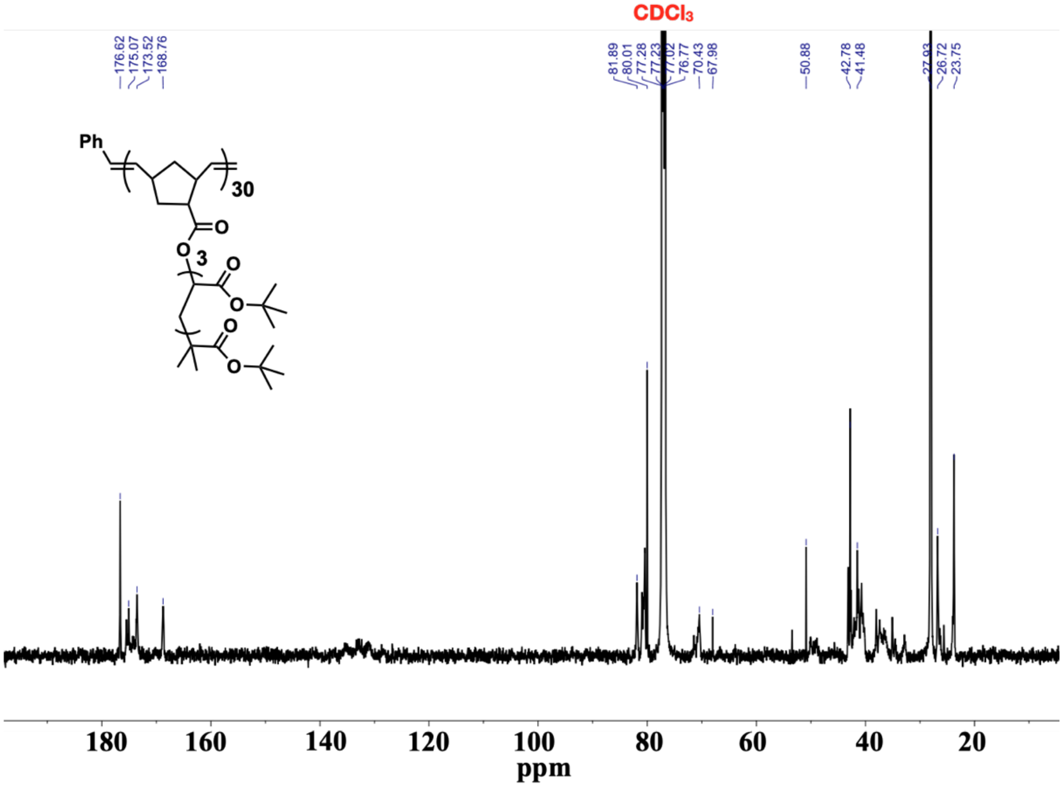

Figure S12. ^{1}H NMR (500 MHz, $CDCl_3$) and ^{13}C NMR (125 MHz, $CDCl_3$) of **PBP-T4$_{30}$.**

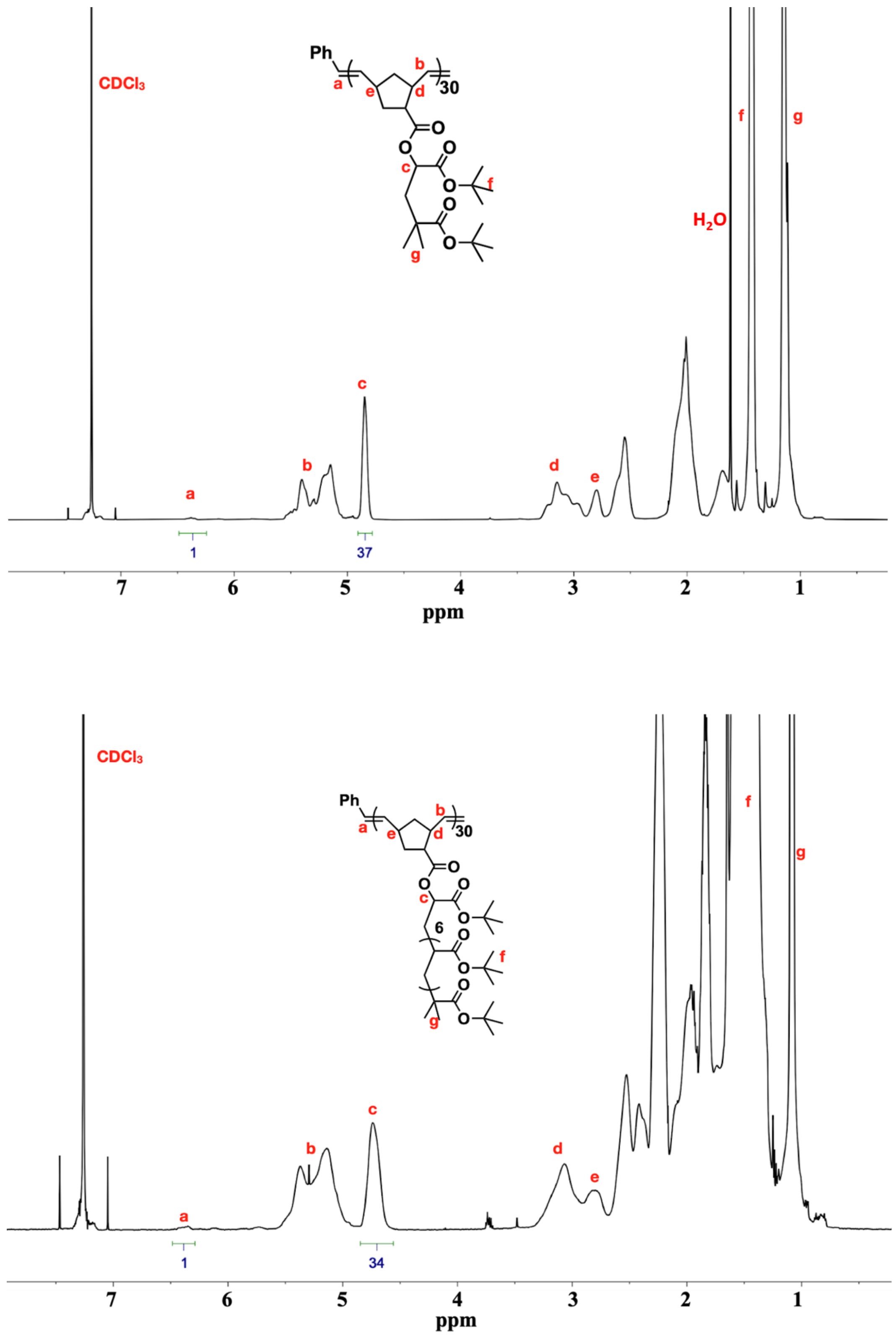

Figure S13. ^{1}H NMR (500 MHz, $CDCl_3$) of **PBP-T2$_{30}$** and **PBP-T8$_{30}$.**

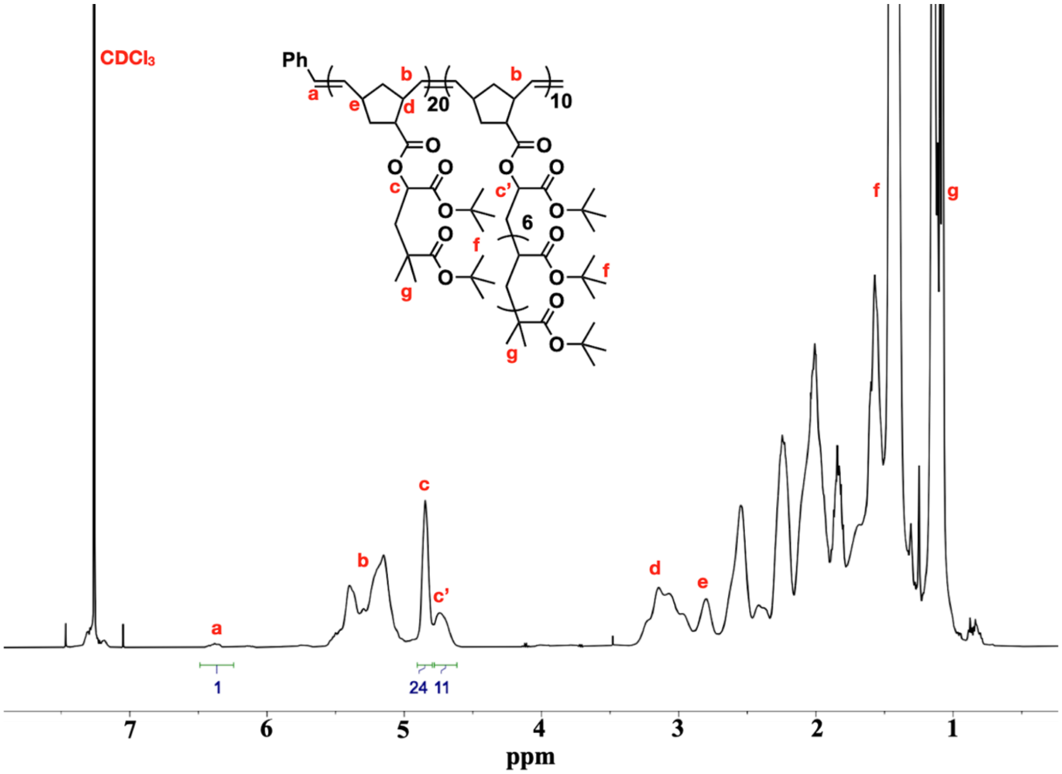

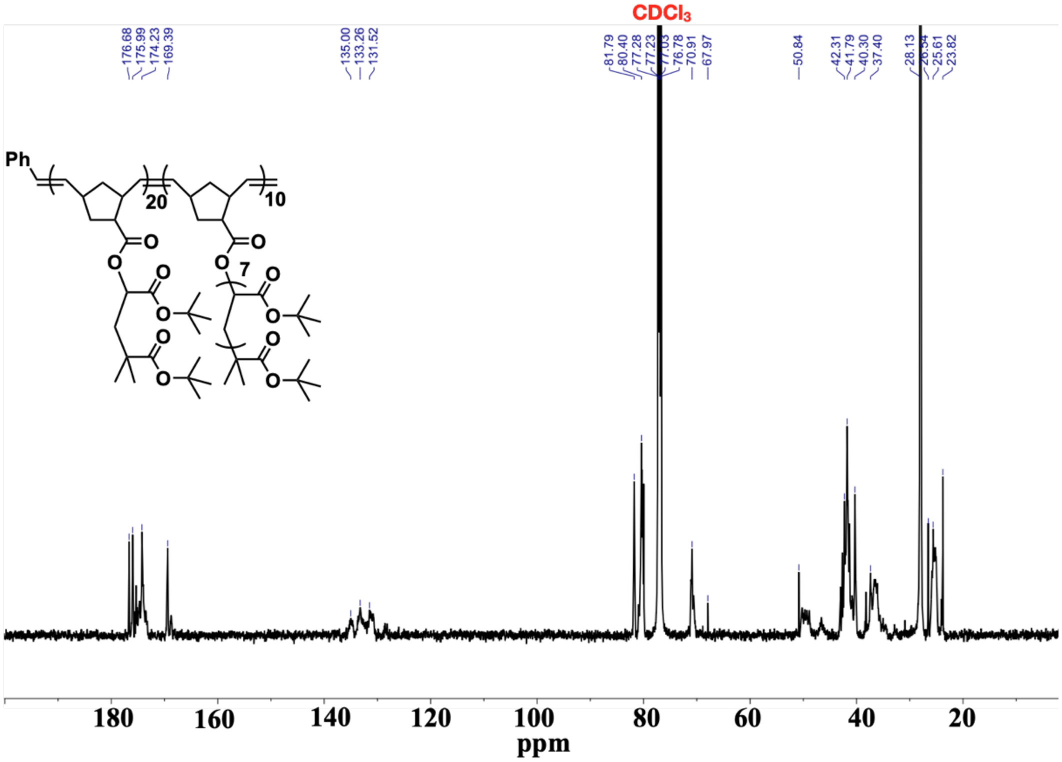

Figure S14. ^{1}H NMR (500 MHz, $CDCl_3$) and ^{13}C NMR (125 MHz, $CDCl_3$) of **b-PBP-T2$_{20}$T8$_{10}$.**

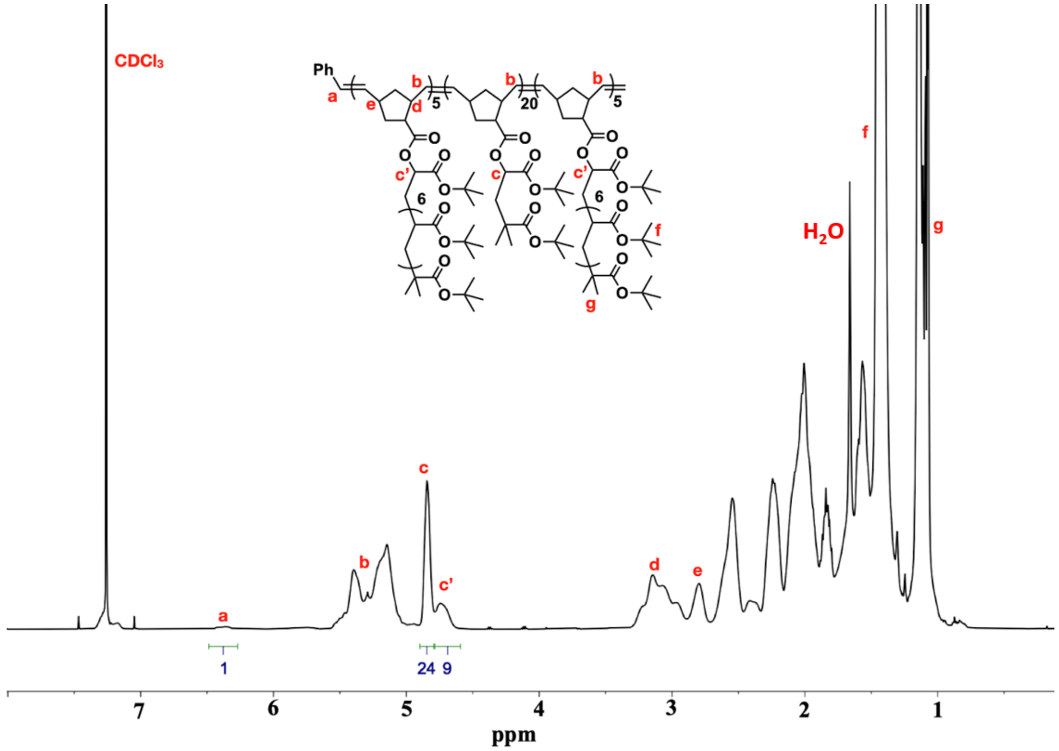

Figure S15. ^{1}H NMR (500 MHz, $CDCl_3$) of **b-PBP-T8$_5$T2$_{20}$T8$_5$.**

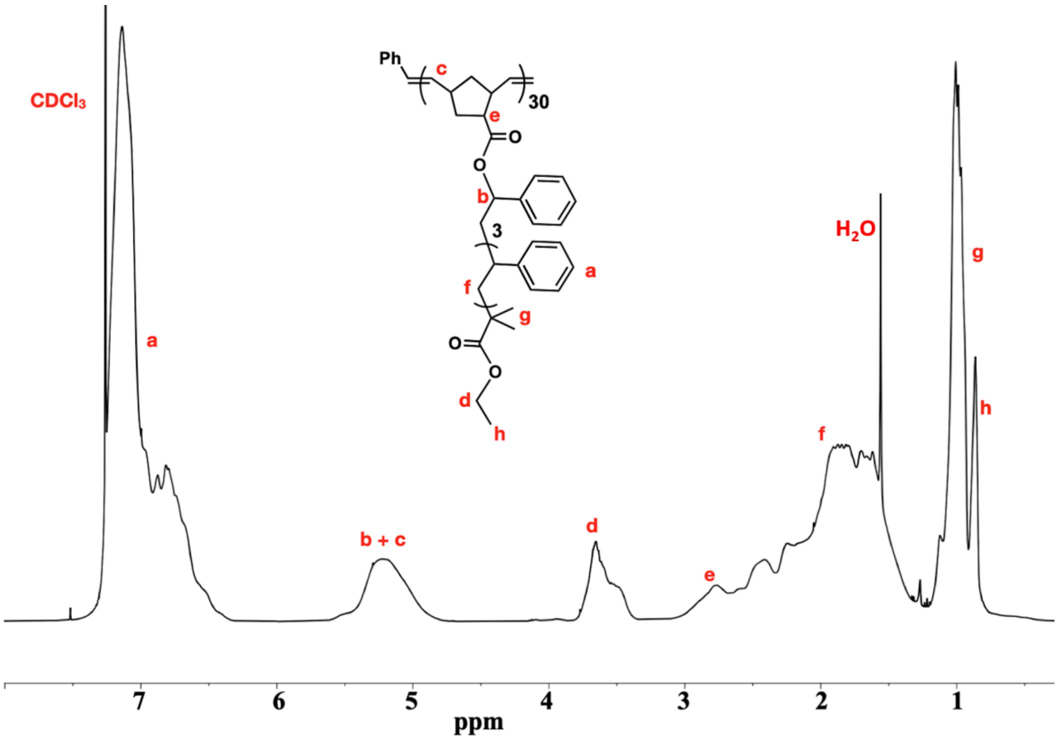

Figure S16. ^{1}H NMR (500 MHz, $CDCl_3$) of **BBP-oSty4$_{30}$.**

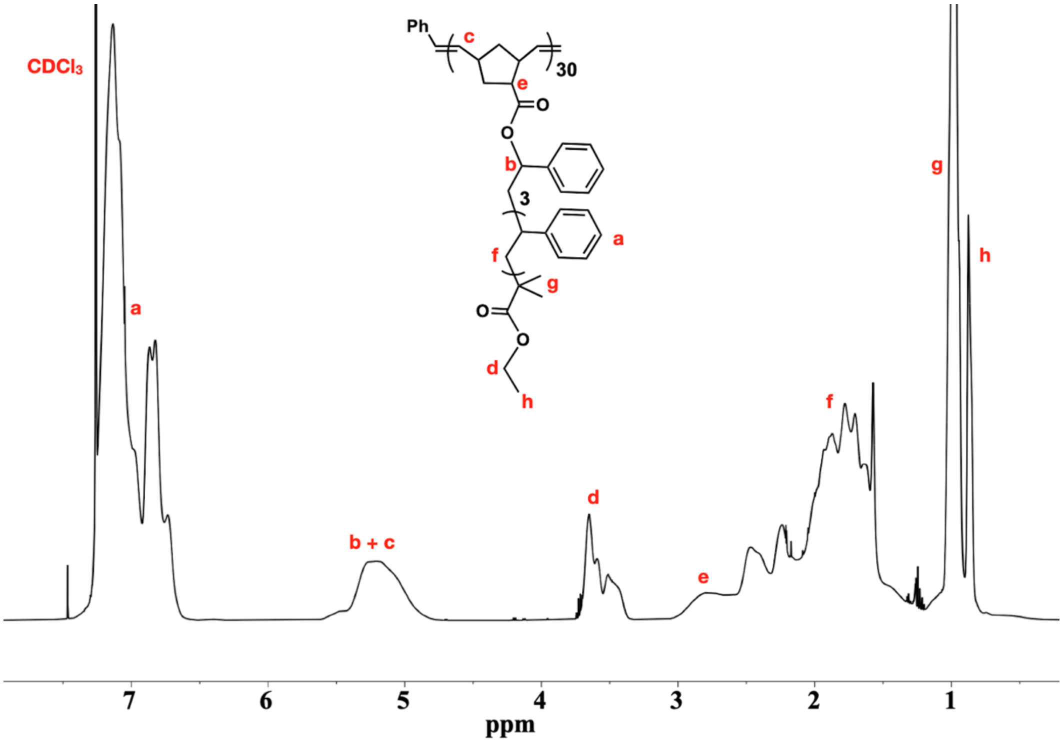

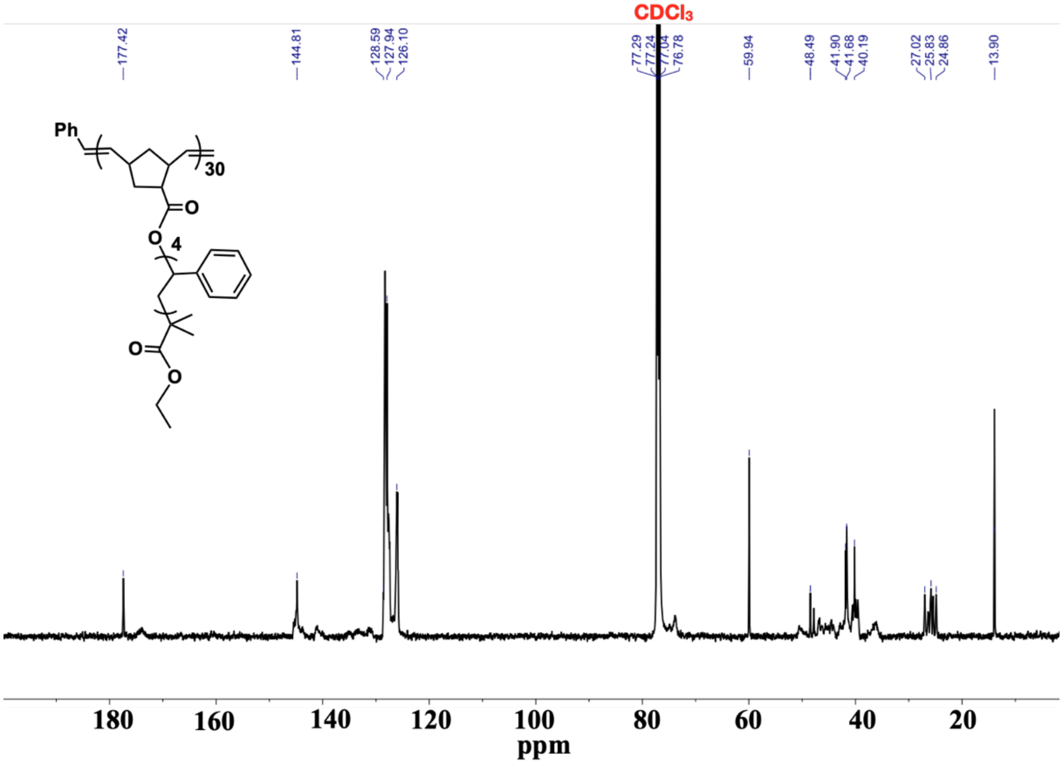

Figure S17. ^{1}H NMR (500 MHz, $CDCl_3$) and ^{13}C NMR (125 MHz, $CDCl_3$) of **PBP-S4$_{30}$.**

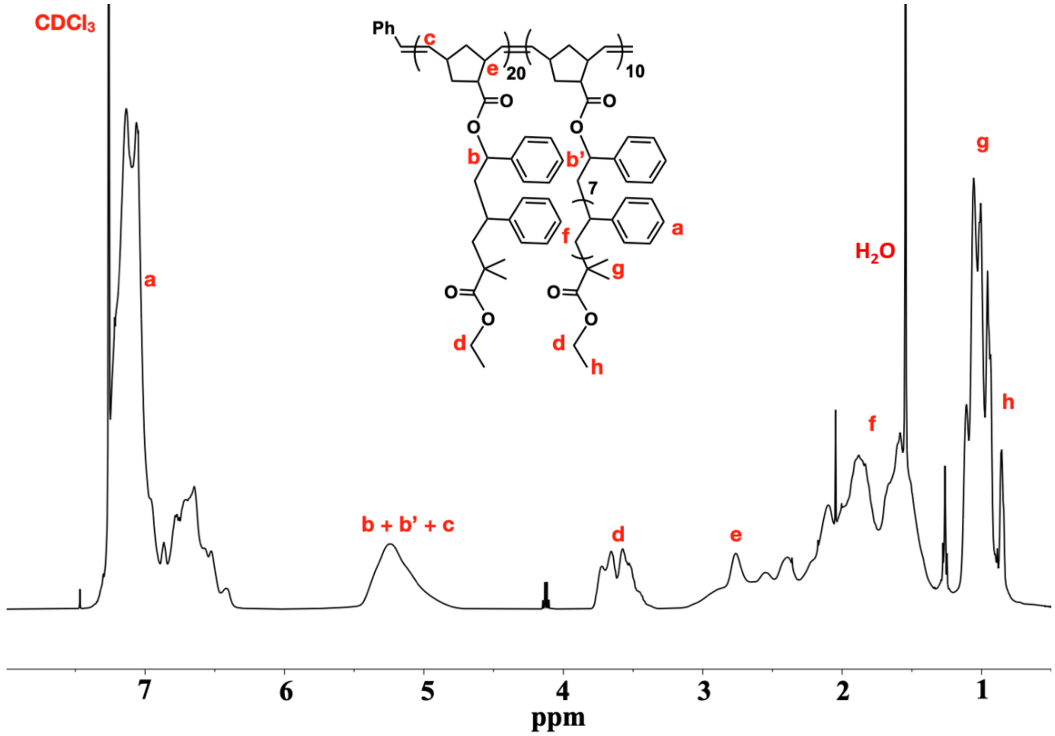

Figure S18a. ^{1}H NMR (500 MHz, $CDCl_3$) of **b-PBP-S2$_{20}$S8$_{10}$.**

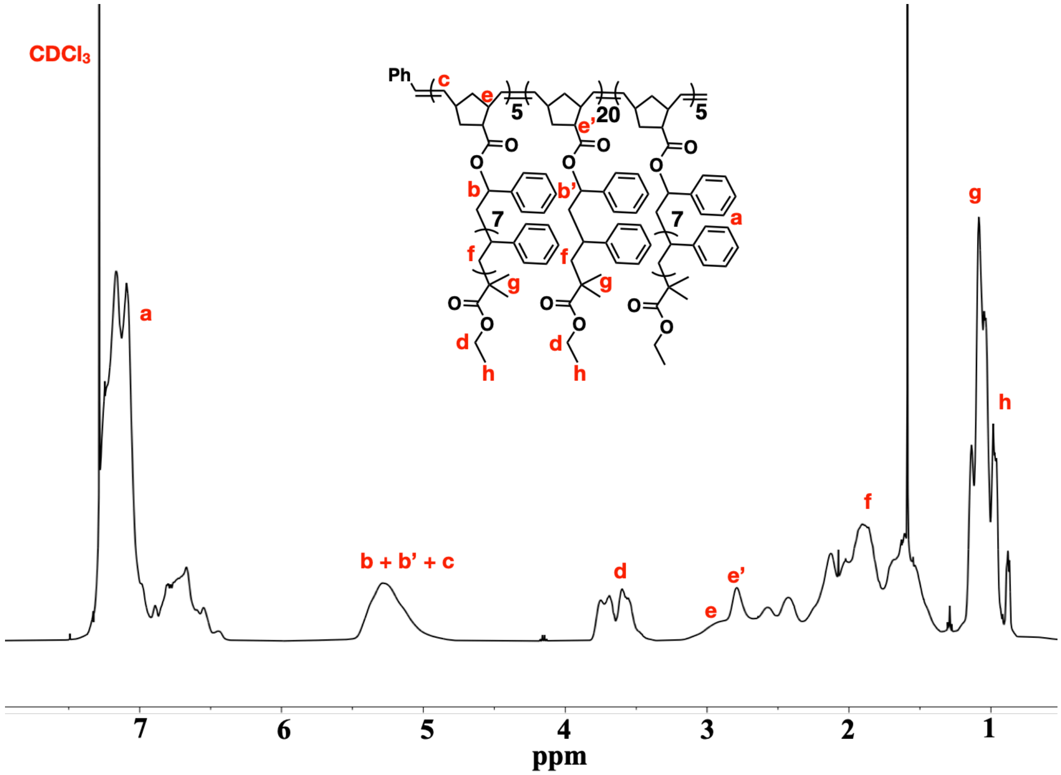

Figure S18b. ^{1}H NMR (500 MHz, $CDCl_3$) of **b-PBP- S8$_{5}$S2$_{20}$S8$_{5}$.**

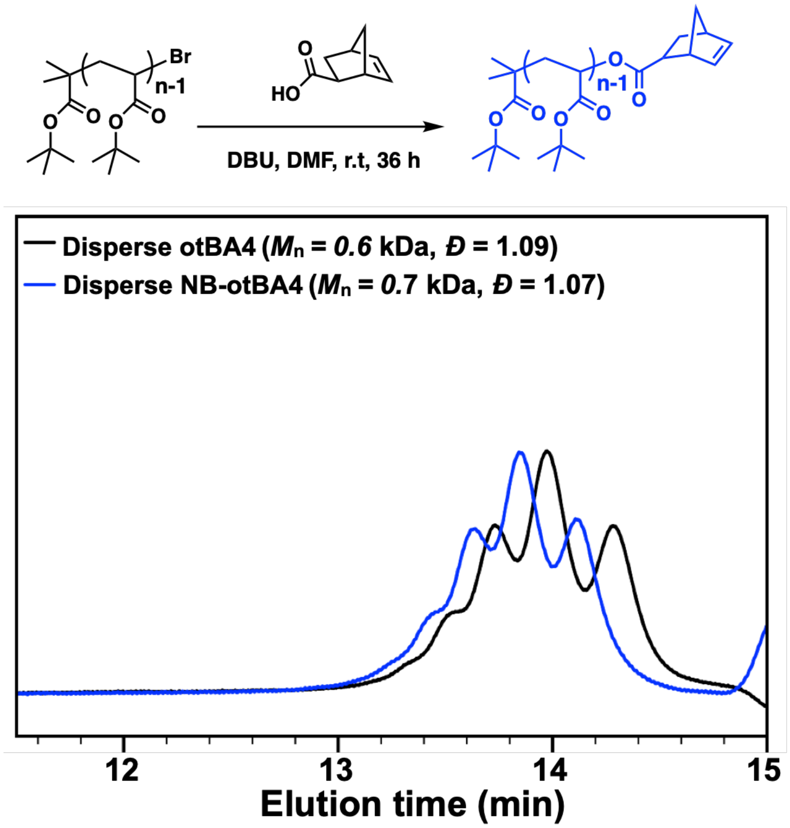

Figure S19. SEC profile of **NB-oTBA4** (blue) after esterification of **oTBA4** (black).

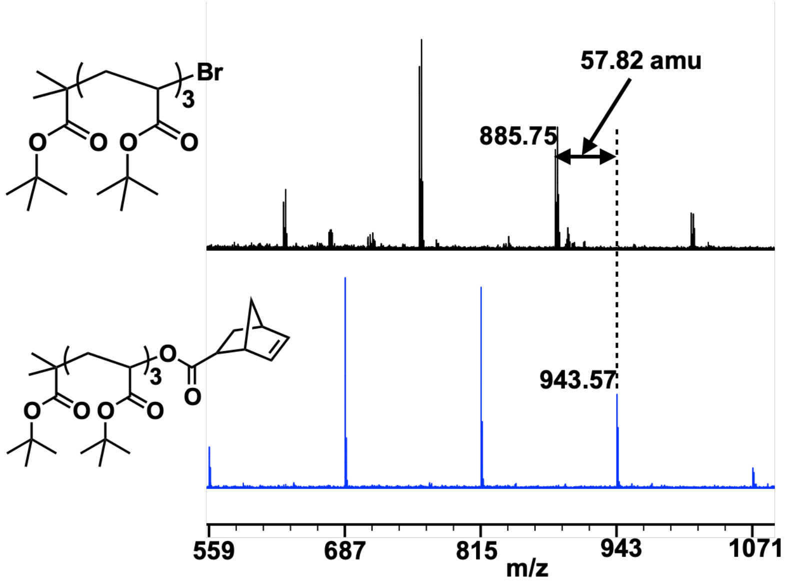

Figure S20. MALDI-ToF spectra of **NB-oTBA4** (blue) after esterification of **oTBA4** (black) showing a difference in m/z of 57.82 amu.

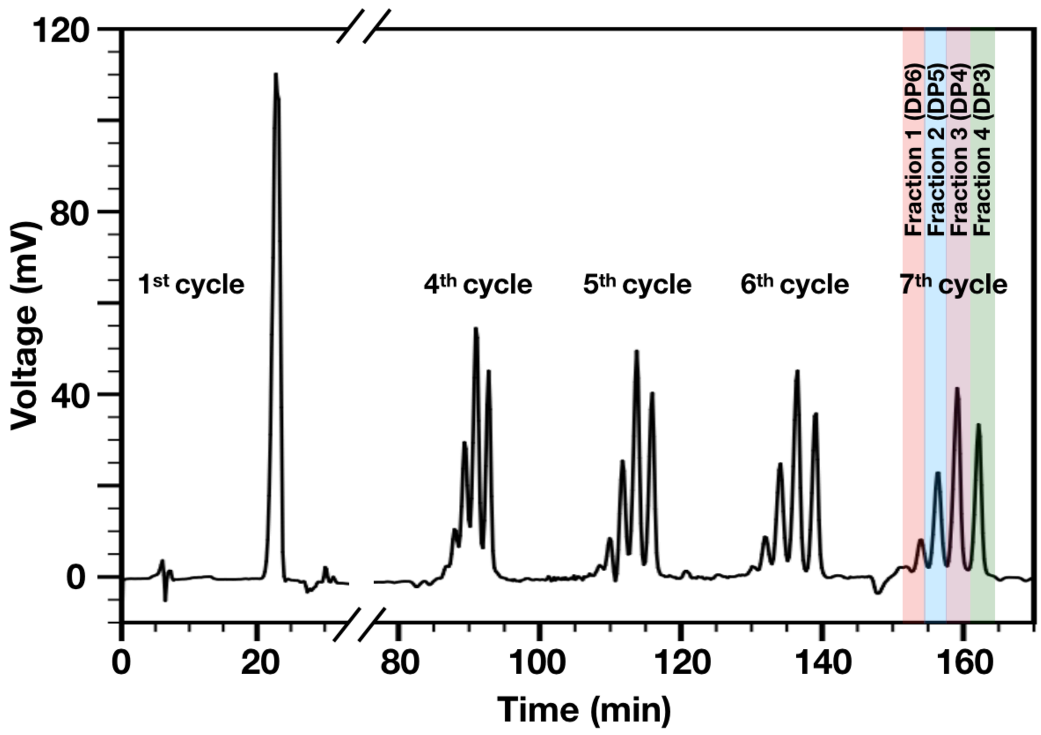

Figure S21. rSEC trace of disperse **NB-oTBA4** separation into discrete libraries.

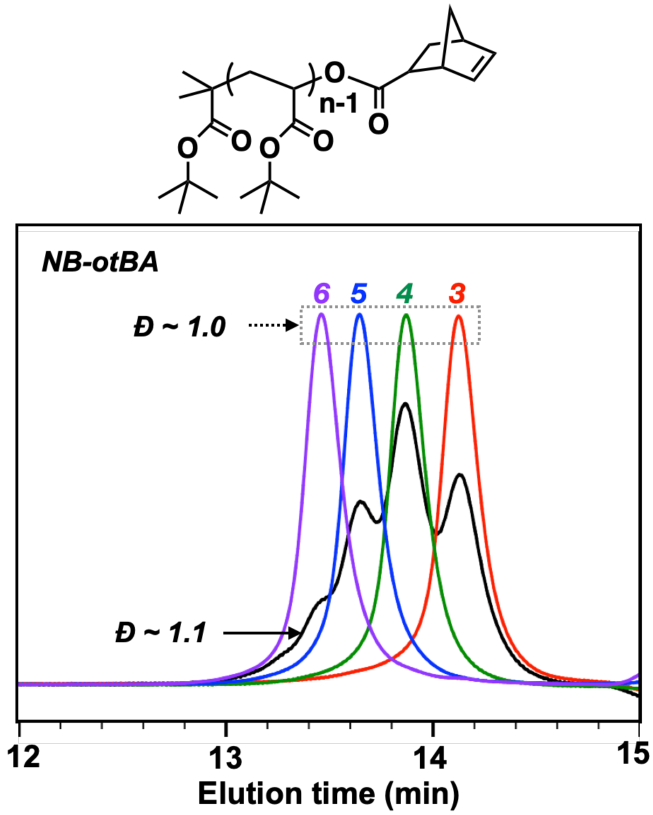

Figure S22. SEC trace of disperse **NB-oTBA4** and the isolated macromonomer libraries (**T3**–**T6**).

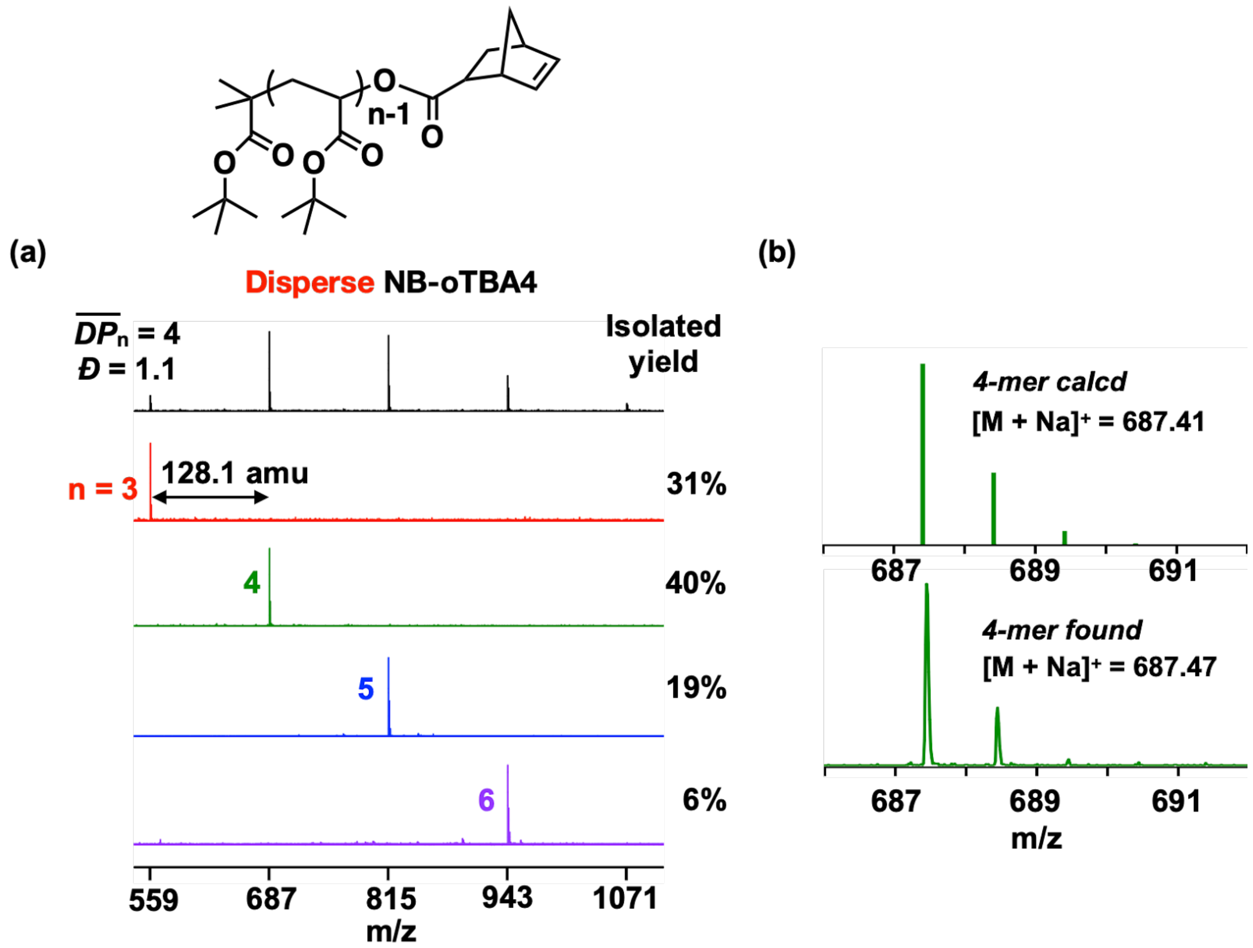

Figure S23. (a) MALDI-ToF spectra of discrete macromonomers **T3** (red), **T4** (green), **T5** (blue), and **T6** (purple) with isolated yields after chromatographic separation of disperse **NB-oTBA4** (black). (b) Calculated and observed isotopic mass distribution of discrete 4-mer (**T4**).

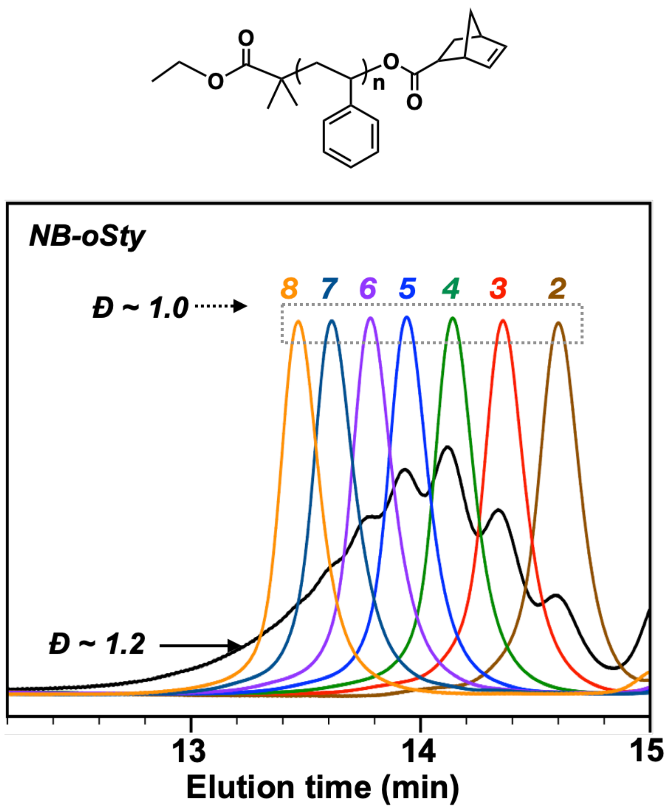

Figure S24. SEC trace of disperse **NB-oSty4** (black) and the isolated macromonomer libraries (**S2**–**S8**).

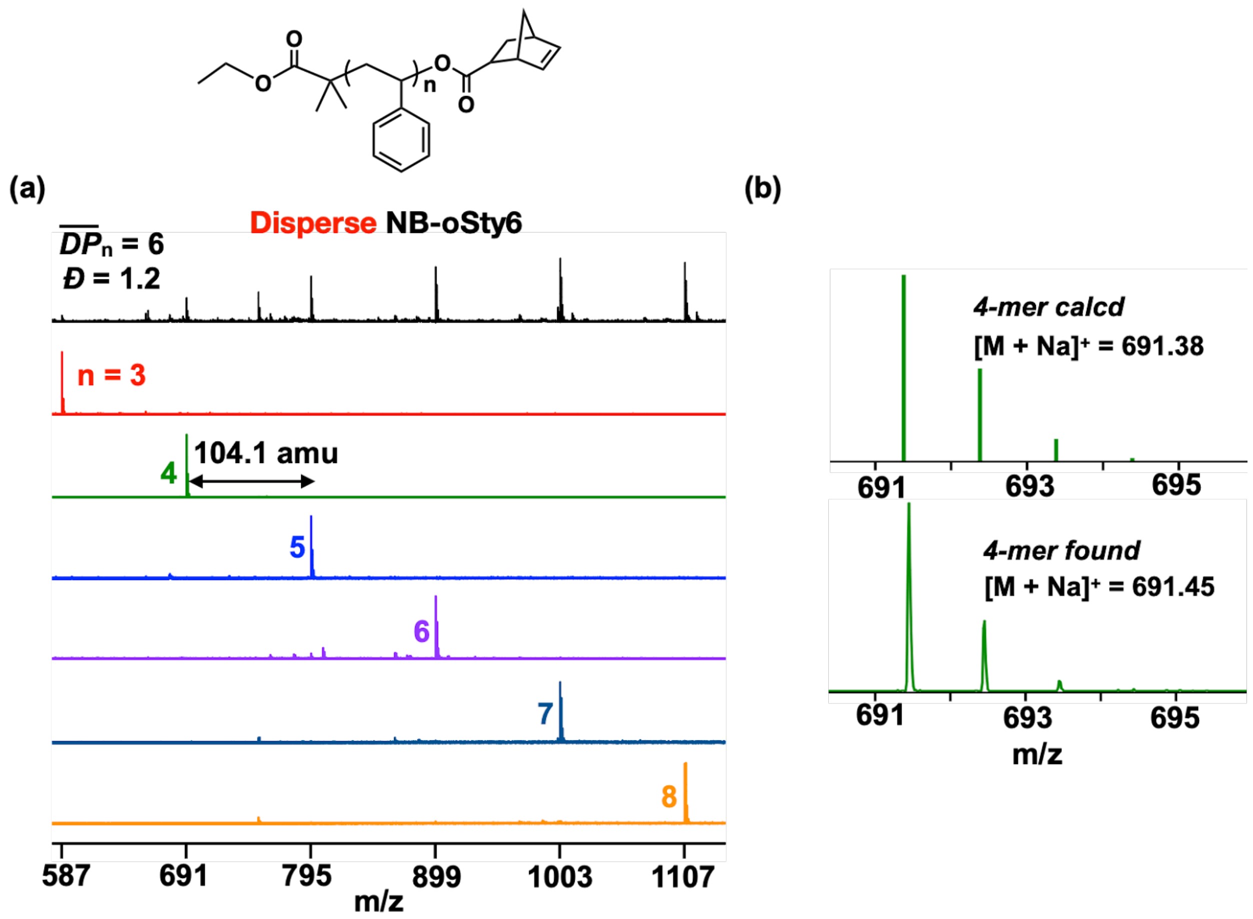

Figure S25. (a) MALDI-ToF spectra of discrete macromonomers **S3** (red), **S4** (green), **S5** (blue), **S6** (purple), **S7** (navy), and **S8** (orange) after chromatographic separation of disperse **NB-oSty6**. (b) Calculated and observed isotopic mass distribution of discrete 4-mer (**S4**).

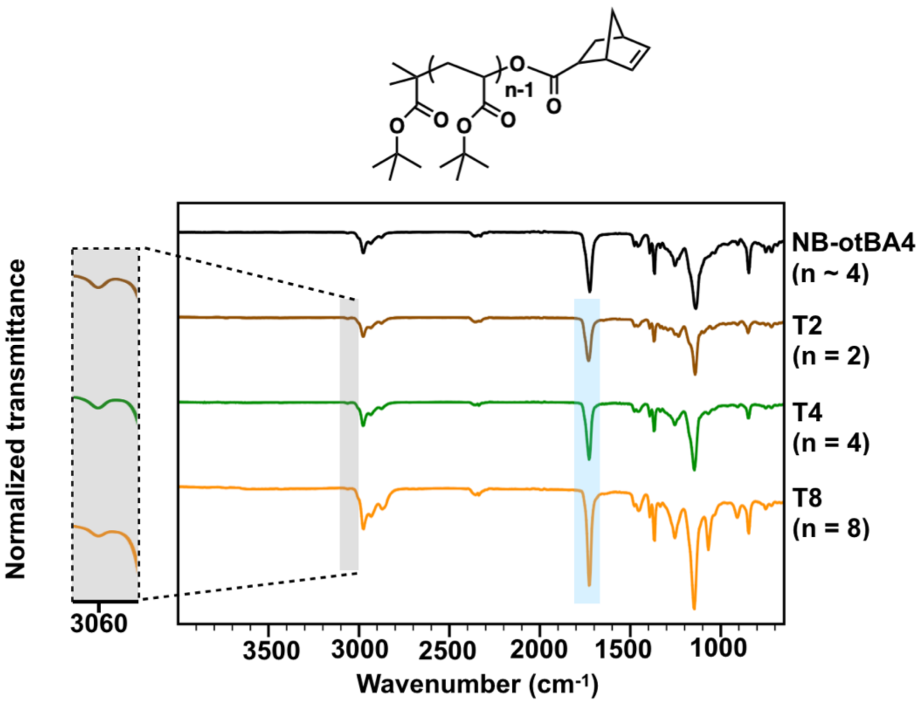

Figure S26. FTIR spectra of **NB-oTBA4** (black), **T2** (brown), **T4** (green), and **T8** (orange). The intensity of the C=O stretch (1720 cm^{-1}) increases with the length of discrete macromonomers (T2, T4, and T8). The spectra were normalized using the vibrational frequency of the olefin (=C–H stretch, 3000 - 3100 cm^{-1}, inset).

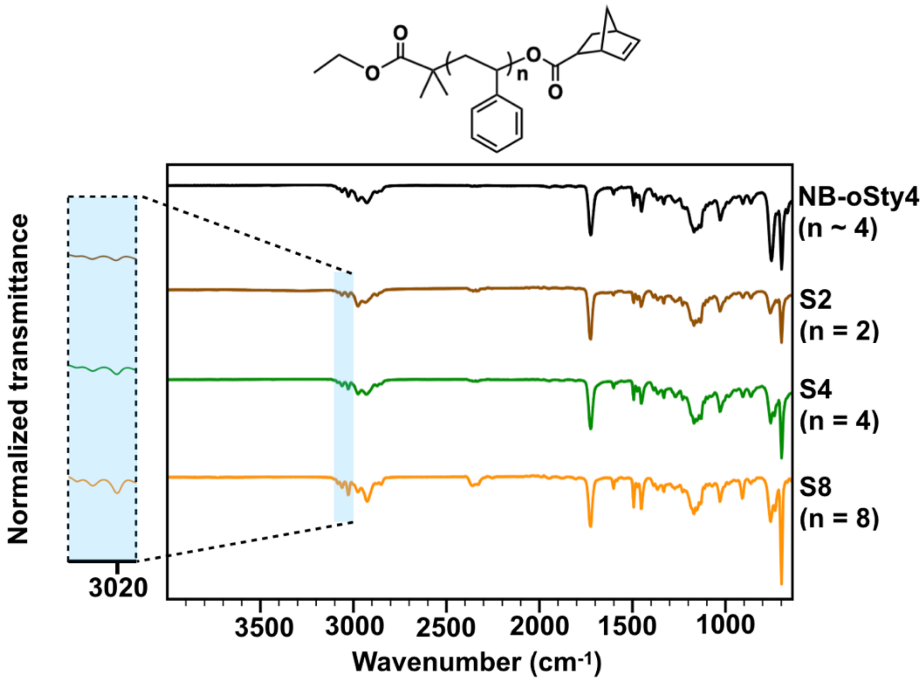

Figure S27. FTIR spectra of **NB-oSty4** (black), **S2** (brown), **S4** (green), and **S8** (orange). The intensity of the =C–H stretch (3000 – 3100 cm^{-1}, inset) corresponding to the aromatic ring increases with the length of discrete macromonomers (S2, S4, and S8). The spectra were normalized using the vibrational frequency of the carbonyl group (C=O stretch, 1720 cm^{-1}).

Kinetic analysis of bottlebrush synthesis

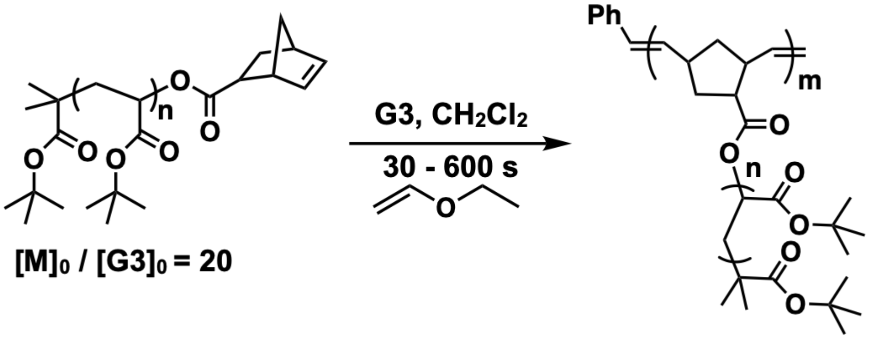

Discrete NB-otBA3 (T3)

Grubbs 3rd generation catalyst (G3) was prepared following reported procedures,[2] and DCM was degassed with Ar for 30 min prior to use.

To an oven-dried 4 mL vial equipped with a stir bar, **T3** (30 mg, 20 equiv.) was dissolved in DCM (0.4 mL) and degassed with Ar for 20 min. A degassed solution of **G3** (1 equiv., 80 mM) was injected into the mixture to initiate the polymerization. The mixture was stirred at room temperature and aliquots were collected at 30, 60, 120, 300, and 600 s followed by quenching with excess ethyl vinyl ether (EVE).

To determine macromonomer conversion, SEC analysis was performed by comparing the areas under the bottlebrush polymer and residual macromonomer peaks (**Figure S28**).[3] NMR analysis was performed by monitoring the integration of the olefinic proton signal at 6.1 ppm relative to the methine proton signal at 4.75 ppm (**Figure S29**).

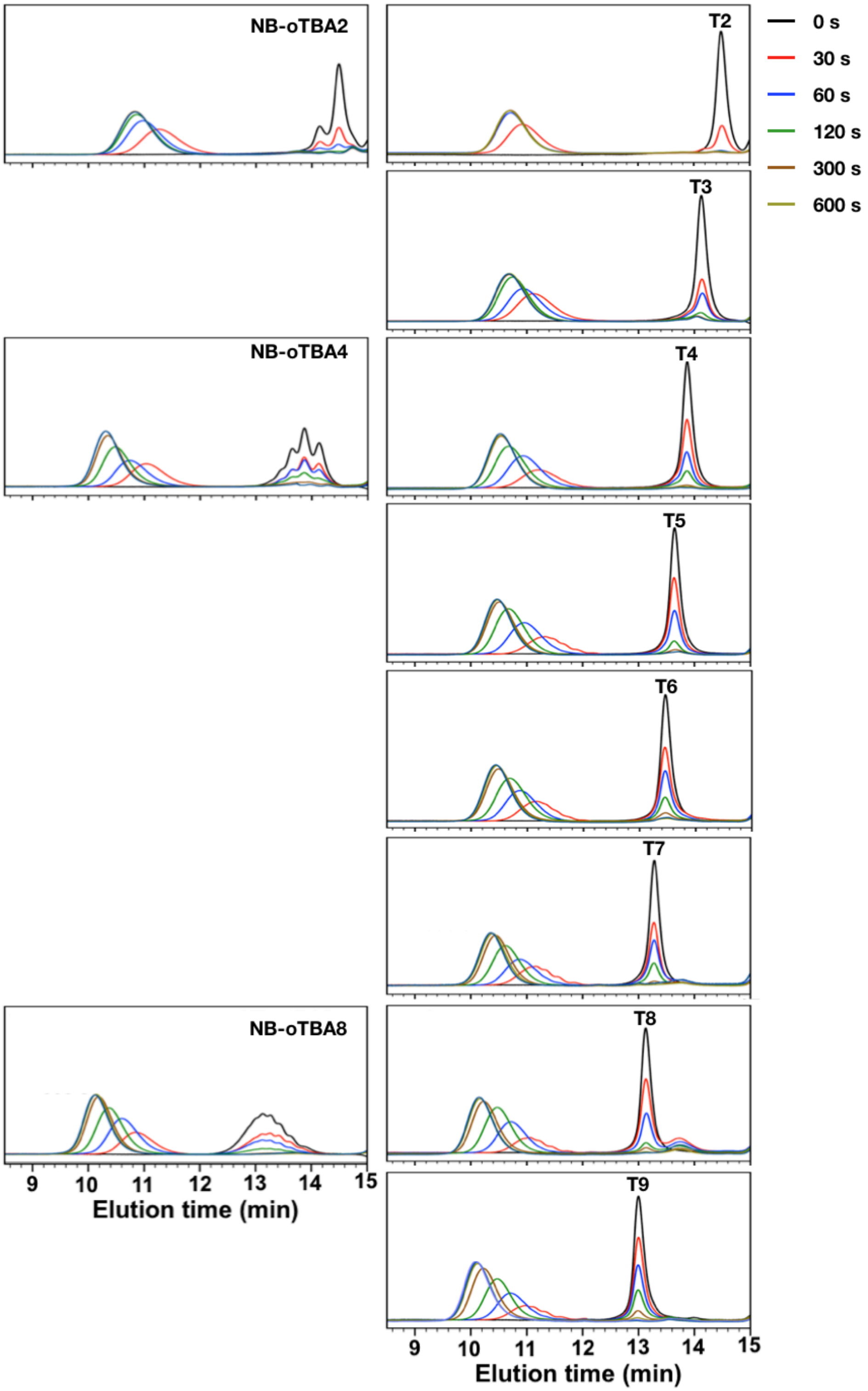

Figure S28. SEC traces depicting macromonomer conversion over time. The total peak areas of all SEC traces were normalized to unity. Macromonomer conversion values were determined by taking the ratio of the areas under bottlebrush polymer and residual macromonomer peaks.

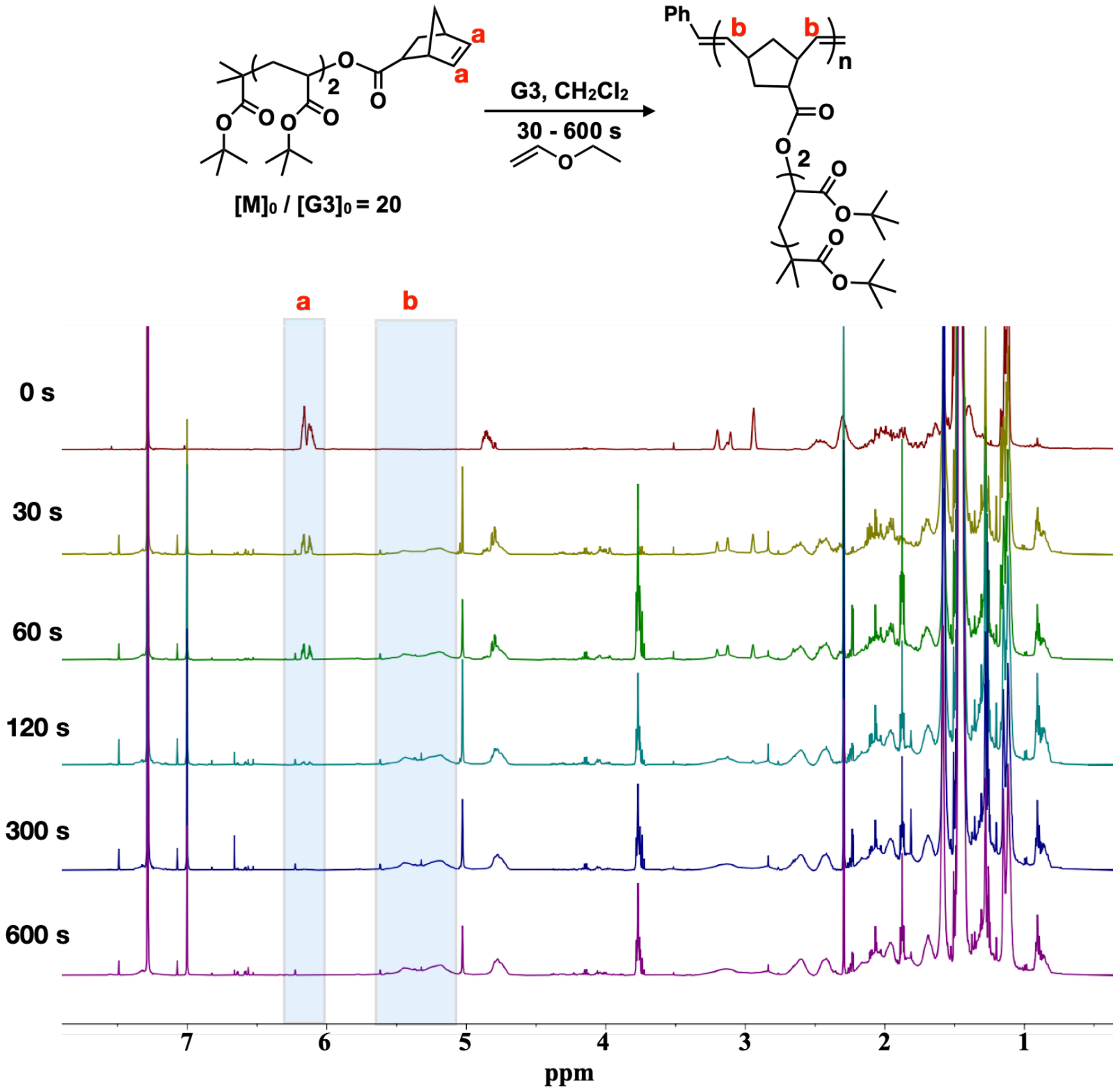

Figure S29. ^{1}H NMR spectra of each aliquot collected at specified time intervals during ROMP of **T3**.

Table S2. Propagation rate of discrete oTBA macromonomers[a]

entry	macromonomer[a]	k_p (10^{-3} s^{-1})[b]	conv.[c] (%)
1	T2	26	> 99
2	T3	19.2	> 99
3	T4	15.7	> 99
4	T6	13.8	> 99
5	T9	11.8	> 99

[a]ROMP was performed with pyridine-ligated G3 catalyst in CH_2Cl_2. $[M]_0/[G3]_0 = 20$ and $[M]_0 = 50$ mM. [b]Estimated using first-order kinetics from SEC conversion data. [c]Monomer conversion after 40 min as determined from the SEC trace of polymer and residual macromonomer.

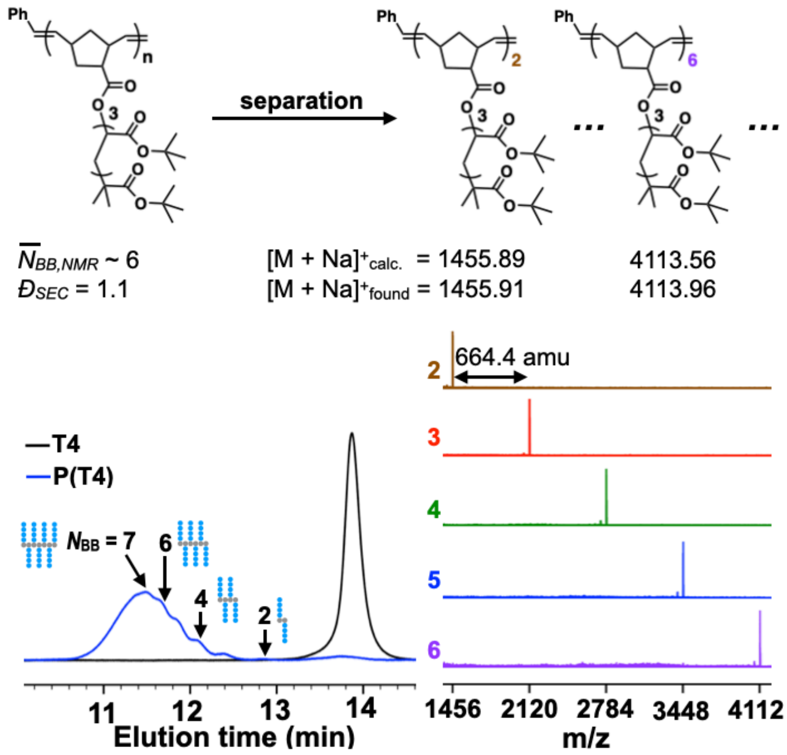

Figure S30a. SEC trace of **PBP-T4$_6$** and MALDI-ToF-MS spectra of **DBP-T4$_n$** libraries (n = 2 to 6) isolated after recycling preparative SEC separation of **PBP-T4$_6$**.

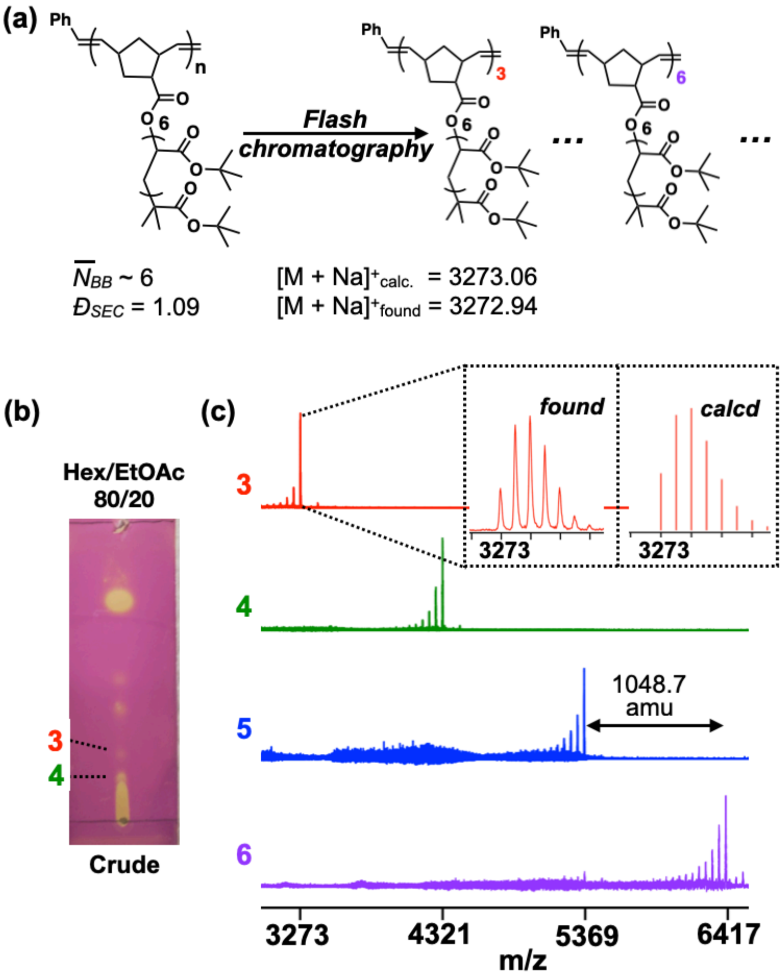

Figure S30b. (a) **DBP-T7** libraries isolated after automated flash chromatographic separation of **PBP-T7$_6$**, illustrating the scalability of the strategy. (b) TLC image of disperse parent material (**PBP-T7$_6$**) eluting with hexane/ethyl acetate = 4/1 (c) MALDI-ToF-MS spectra of aliquots sampled directly from test tubes containing **DBP-T7$_3$**, **DBP-T7$_4$, DBP-T7$_5$**, and **DBP-T7$_6$.**

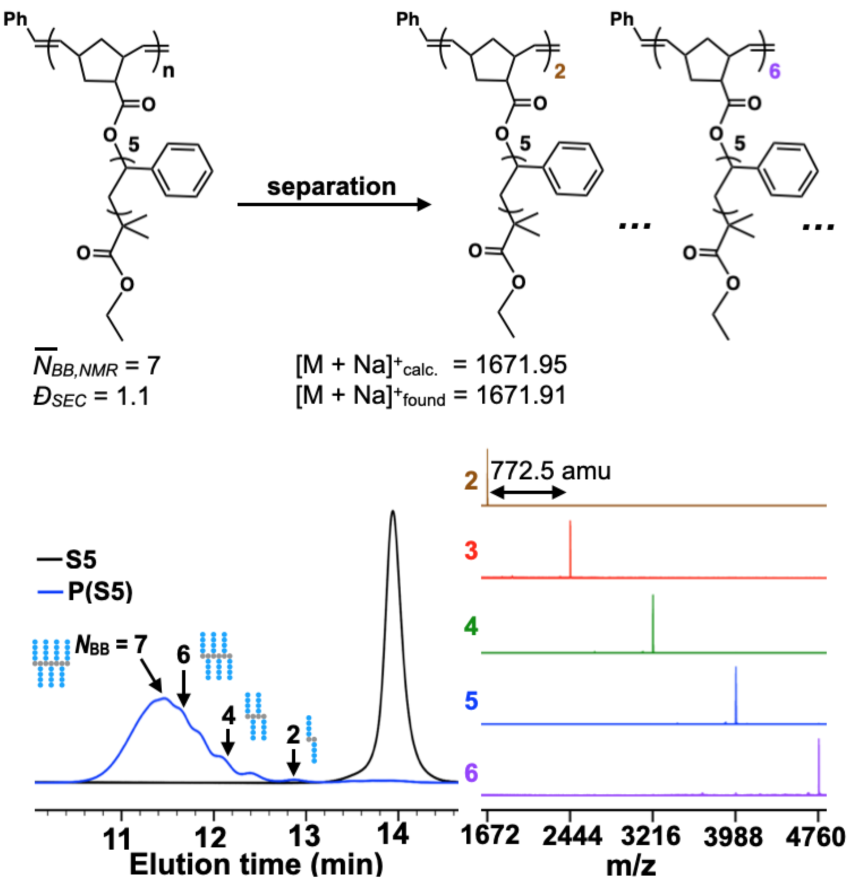

Figure S30c. SEC trace of **PBP-S5$_6$** and MALDI-ToF-MS spectra of **DBP-S5$_n$** libraries (n = 2 to 6) isolated after recycling preparative SEC separation of **PBP-S5$_6$**.

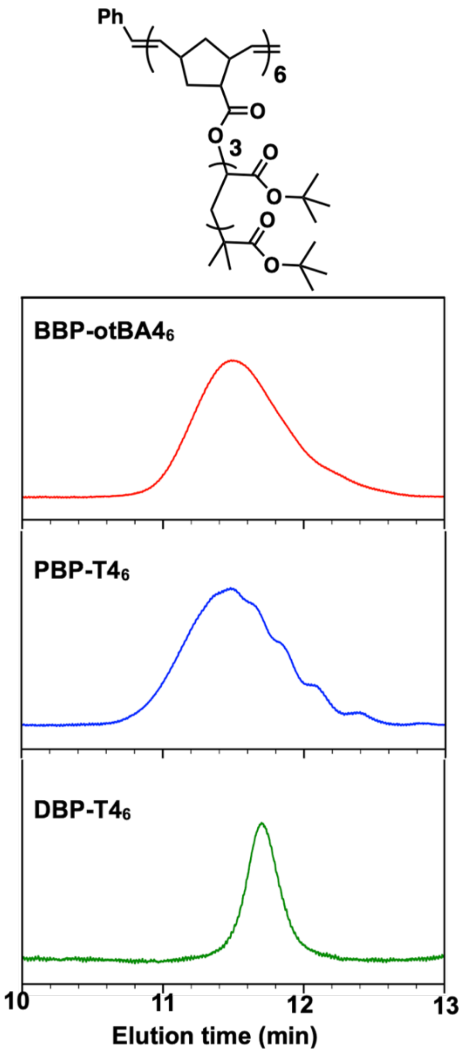

Figure S31. SEC traces of **BBP-oTBA4$_6$** (red, $Đ_{SEC\text{-}RI}$ = 1.07), **PBP-T4$_6$** (blue, $Đ_{SEC\text{-}RI}$ = 1.06), and **DBP-T4$_6$** (green, $Đ_{MALDI\text{-}ToF}$ = 1.0).

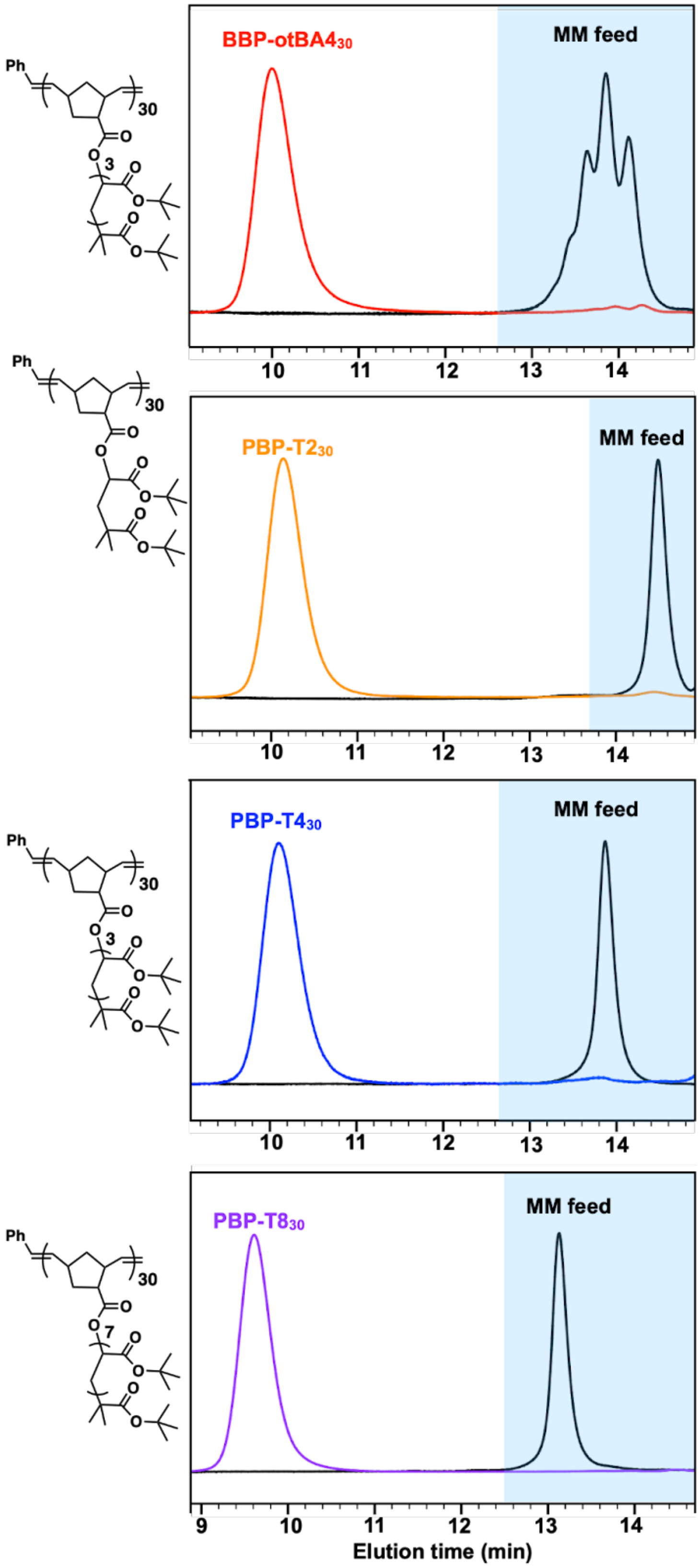

Figure S32. SEC traces of **BBP-oTBA4$_{30}$** (red), **PBP-T2$_{30}$** (orange), **PBP-T4$_{30}$** (blue), and **PBP-T8$_{30}$** (purple).

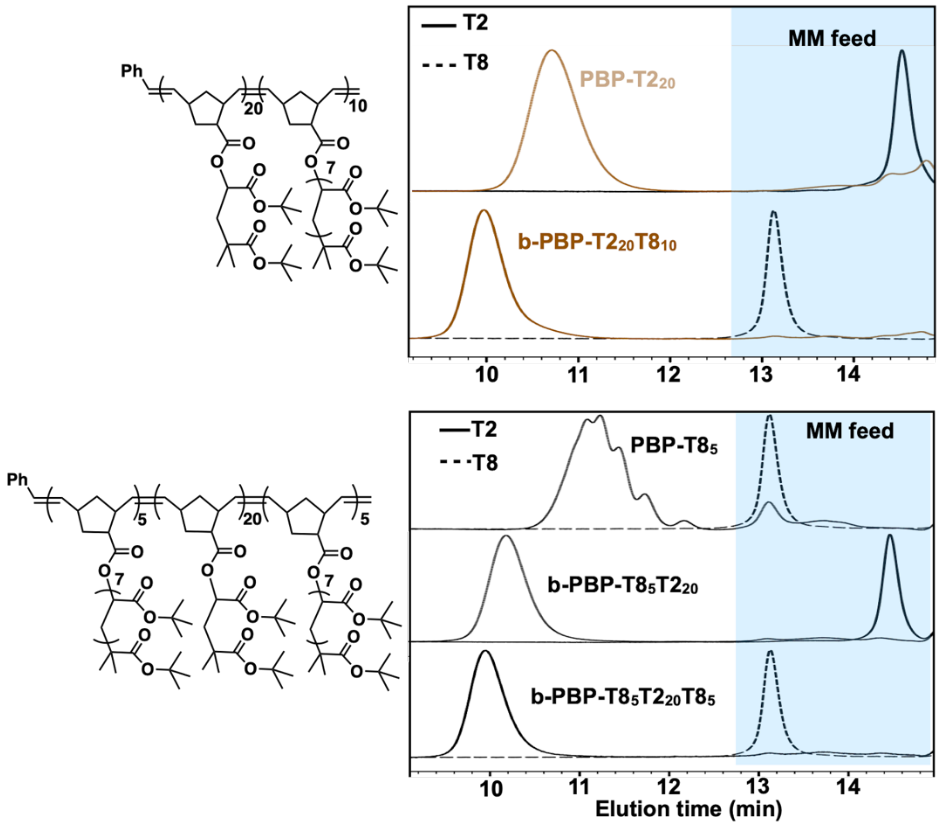

Figure S33. SEC traces of diblock **b-PBP-T2$_{20}$T8$_{10}$** (brown) and triblock **b-PBP-T8$_{5}$T2$_{20}$T8$_{5}$** (black).

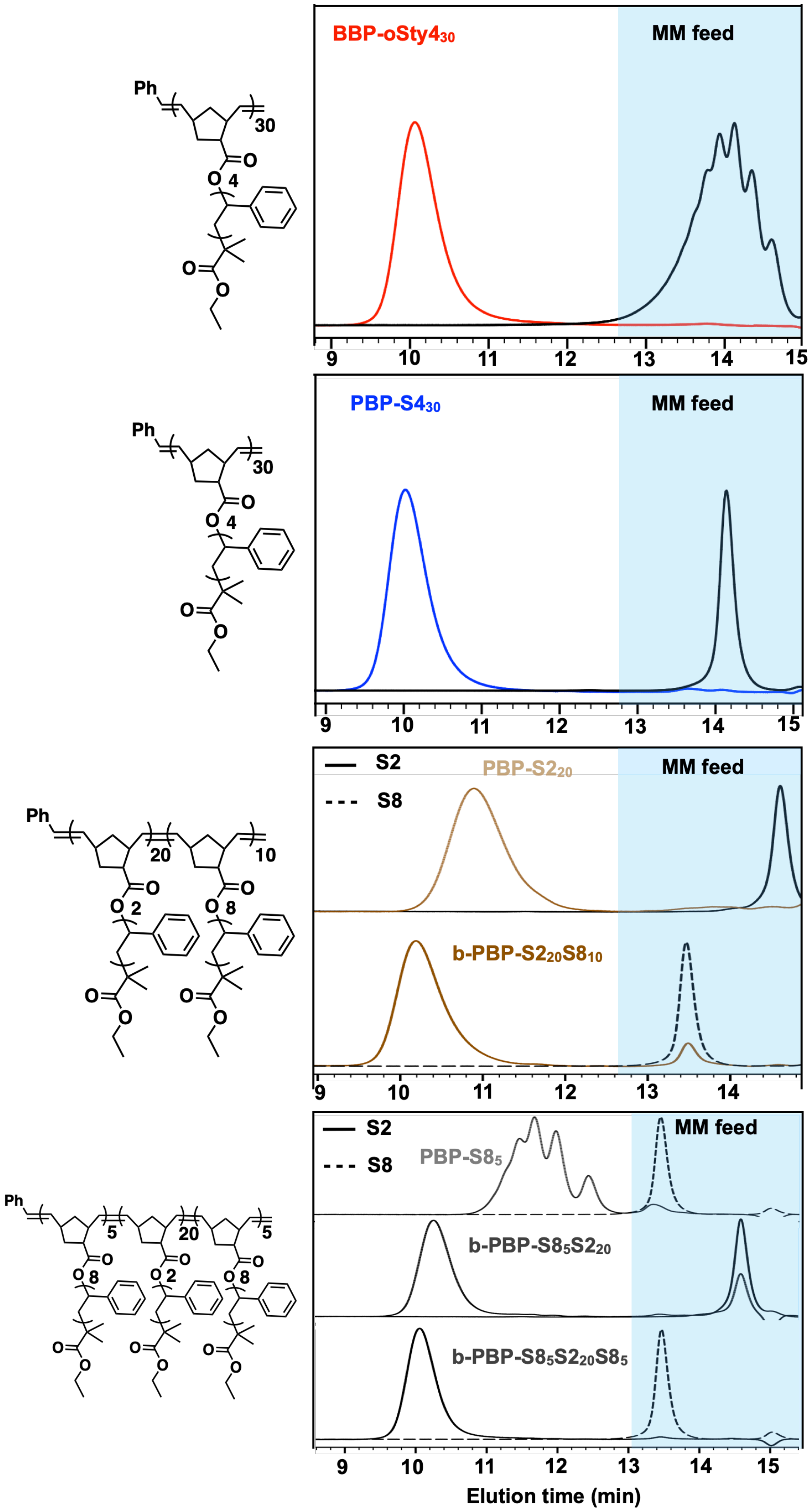

Figure S34. SEC traces of **BBP-oSty4$_{30}$** (red), **PBP-S4$_{30}$** (blue), **b-PBP-S2$_{20}$S8$_{10}$** (brown), and **b-PBP- S8$_{5}$S2$_{20}$S8$_{5}$** (black).

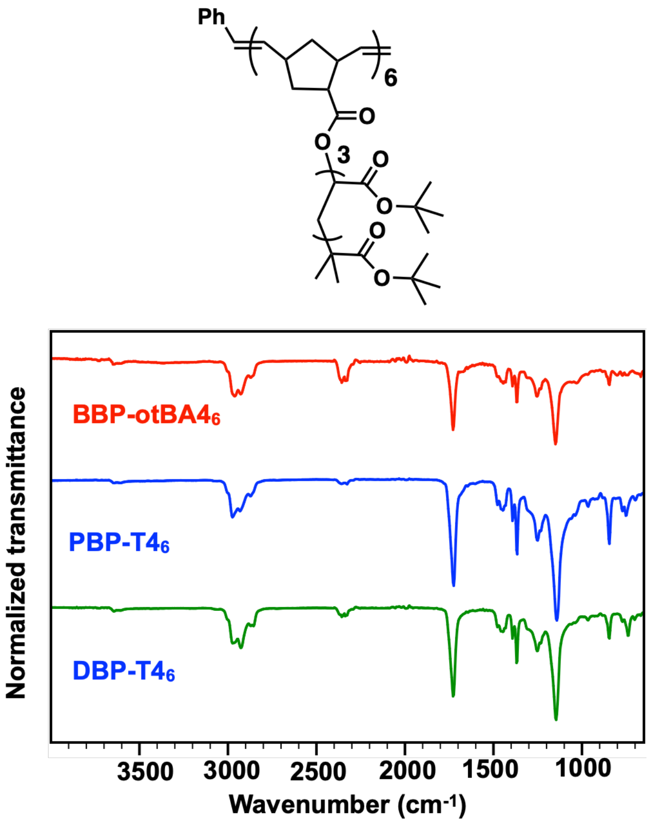

Figure S35. FTIR spectra of **BBP-oTBA4$_6$** (red), **PBP-T4$_6$** (blue), and **DBP-T4$_6$** (green).

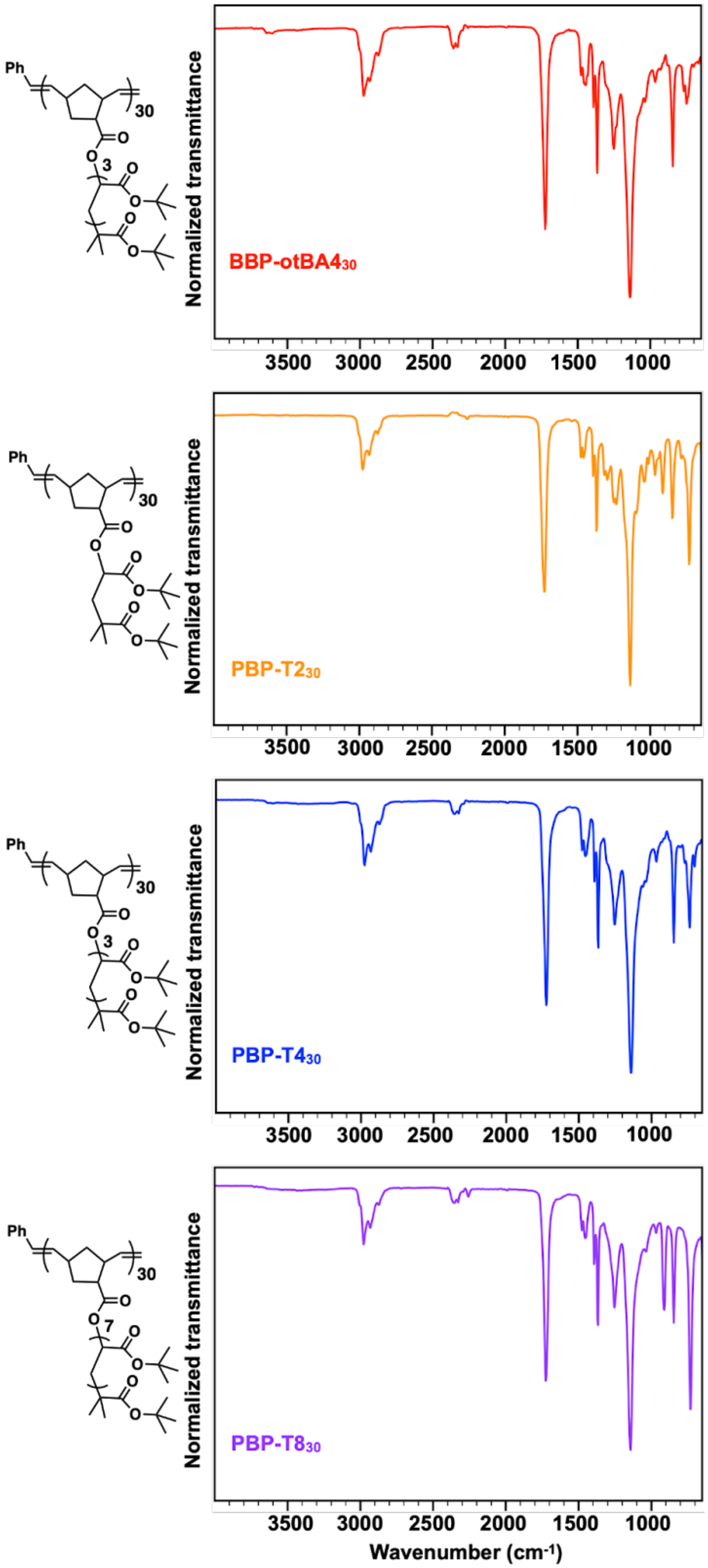

Figure S36. FTIR spectra of **BBP-oTBA4$_{30}$** (red), **PBP-T2$_{30}$** (orange), **PBP-T4$_{30}$** (blue), and **PBP-T8$_{30}$** (purple).

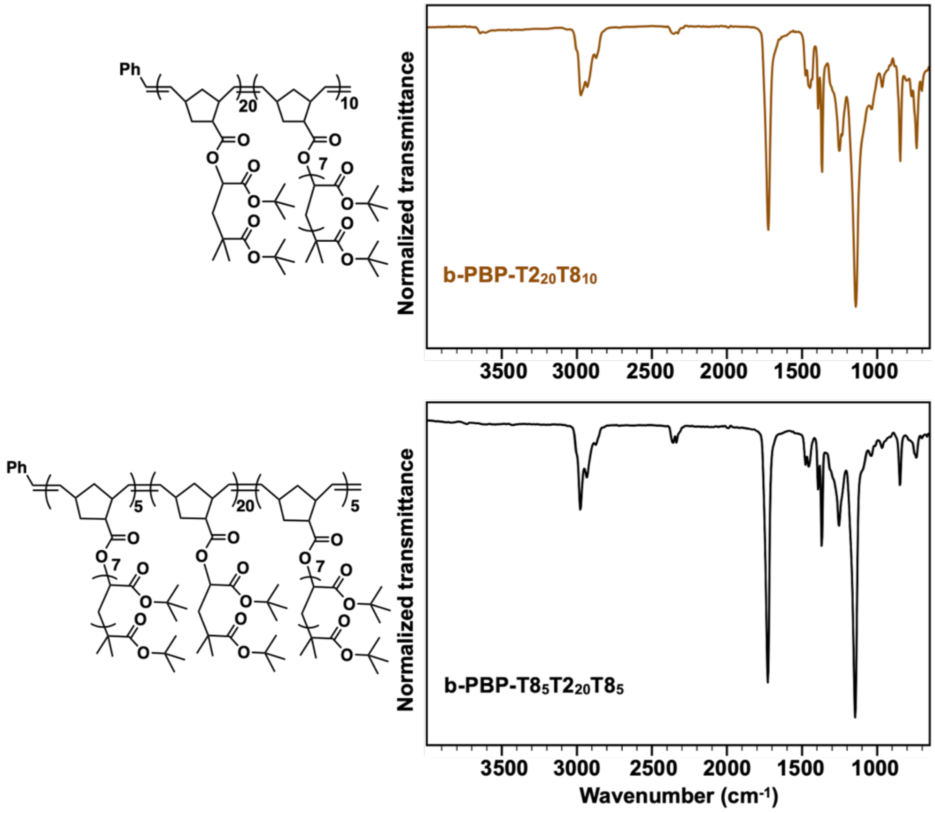

Figure S37. FTIR spectra of diblock **b-PBP-T2$_{20}$T8$_{10}$** (brown) and triblock **b-PBP-T8$_5$T2$_{20}$T8$_5$** (black).

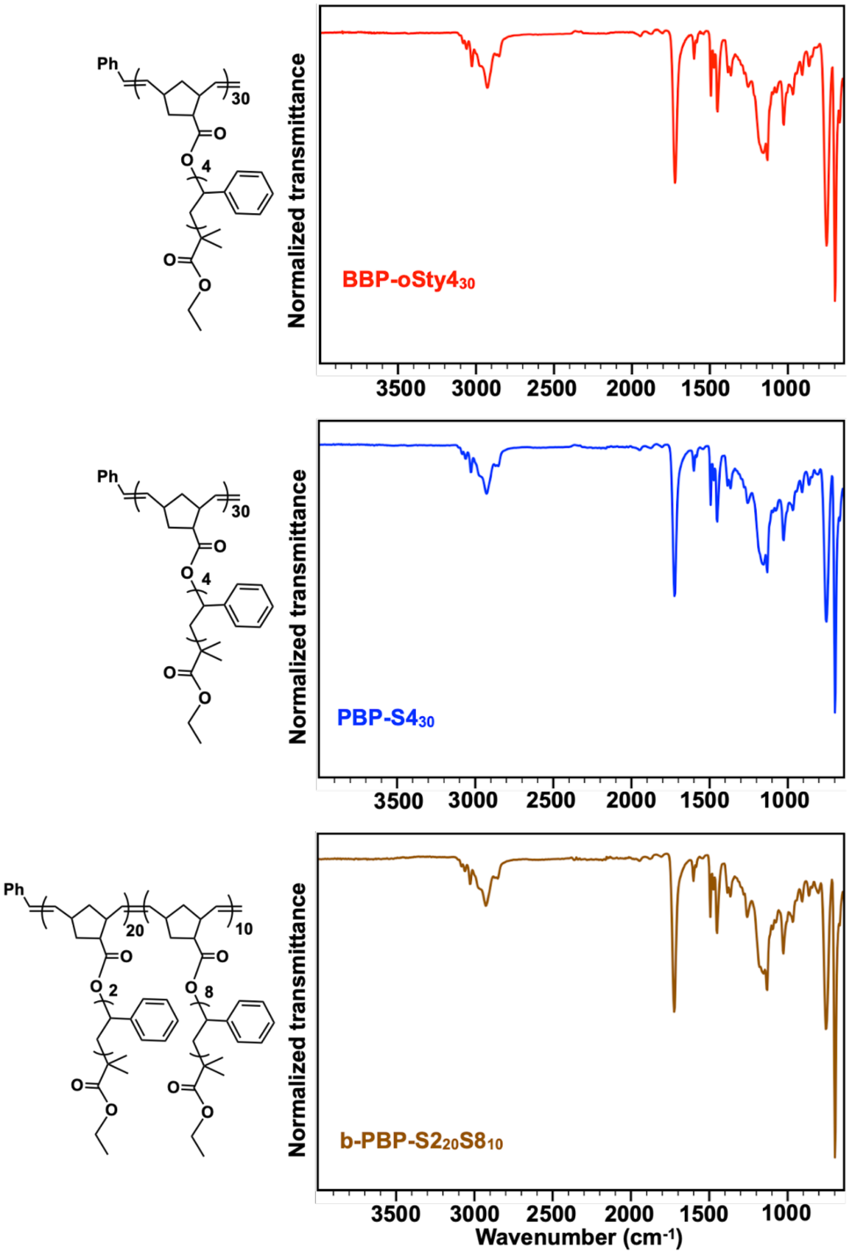

Figure S38. FTIR spectra of **BBP-oSty4$_{30}$** (red), **PBP-S4$_{30}$** (blue), and **b-PBP-S2$_{20}$S8$_{10}$** (brown).

Table S3. Bottlebrush polymers synthesized and used in this study

A. Disperse bottlebrush polymers (BBPs)

entry	sample[a]	macromonomer	bottlebrush $M_{n,\,theo}$[b] (kDa)	$M_{n,\,exp}$[c] (kDa)	$Đ$[c]	N_{BB}[c]
1	BBP-oTBA4$_{30}$	NB-otBA4	19.9	24.0	1.02	34
2	BBP-oTBA4$_{6}$	NB-otBA4	4.0	4.8	1.07	7
3	BBP-oSty4$_{30}$	NB-oSty4	20.0	18.7	1.05	30

B. Precision bottlebrush polymers (PBPs) – discrete oligo(*tert*-butyl acylate) side chains

entry	sample[a]	macromonomer	bottlebrush $M_{n,\,theo}$[b] (kDa)	$M_{n,\,exp}$[c] (kDa)	$Đ$[c]	N_{BB}[c]
1	PBP-T4$_{30}$	T4	19.9	20.3	1.01	30
2	b-PBP-T2$_{20}$T8$_{10}$	T2 and T8	19.9	21.7	1.03	32
3	b-PBP-T8$_{5}$T2$_{20}$T8$_{5}$	T2 and T8	19.9	21.0	1.01	31
4	PBP-T2$_{30}$	T2	12.2	14.5	1.01	35
5	PBP-T8$_{30}$	T8	35.3	41.0	1.01	34
6	PBP-T4$_{6}$	T4	4.0	4.4	1.06	6

C. Precision bottlebrush polymers (BBPs) – discrete oligo(styrene) side chains

entry	sample[a]	macromonomer	bottlebrush $M_{\mathrm{n,\,theo}}{}^{b}$ (kDa)	$M_{\mathrm{n,\,exp}}{}^{c}$ (kDa)	$Đ^{c}$	$N_{\mathrm{BB}}{}^{c}$
1	PBP-$S4_{30}$	S4	20.0	20.2	1.03	30
2	b-PBP-$S2_{20}8_{10}$	S2 and S8	20.0	16.1	1.05	24
3	b-PBP-$8_{5}S2_{20}8_{5}$	S2 and S8	20.0	20.3	1.05	30
4	PBP-$S5_{6}$	S5	4.6	5.5	1.04	7

D. Discrete bottlebrush polymers (DBPs) – discrete *tert*-butyl acylate tetramer side chains

entry	sample[a]	bottlebrush $m/z_{\mathrm{,\,calcd}}$	$m/z_{\mathrm{,\,found}}{}^{d}$	$Đ^{d}$	$N_{\mathrm{BB}}{}^{d}$
1	DBP-$T4_{2}$	1455.89	1455.85	1.00	2
2	DBP-$T4_{3}$	2120.31	2120.26	1.00	3
3	DBP-$T4_{4}$	2784.73	2784.66	1.00	4
4	DBP-$T4_{5}$	3449.15	3449.18	1.00	5
5	DBP-$T4_{6}$	4113.56	4113.96	1.00	6

[a]Letters before the hyphen (-): P – precision, D – discrete, BP – bottlebrush polymer. Letter after the hyphen (-) represents the side chain monomer: T – *tert*-butyl acrylate, S – styrene. The regular number represents N_{SC} and the subscripted number represents N_{BB}. [b]$M_{n,theo}$ = $[\mathrm{MM}]_0 / [\mathrm{G3}]_0 \times M_{\mathrm{n,macromonomer}} \times$ conversion (%). [c]Measured by SEC-MALS, $N_{\mathrm{BB}} = M_{\mathrm{n,bottlebrush}} / M_{\mathrm{n,macromonomer}}$. [d]Measured by MALDI-ToF.

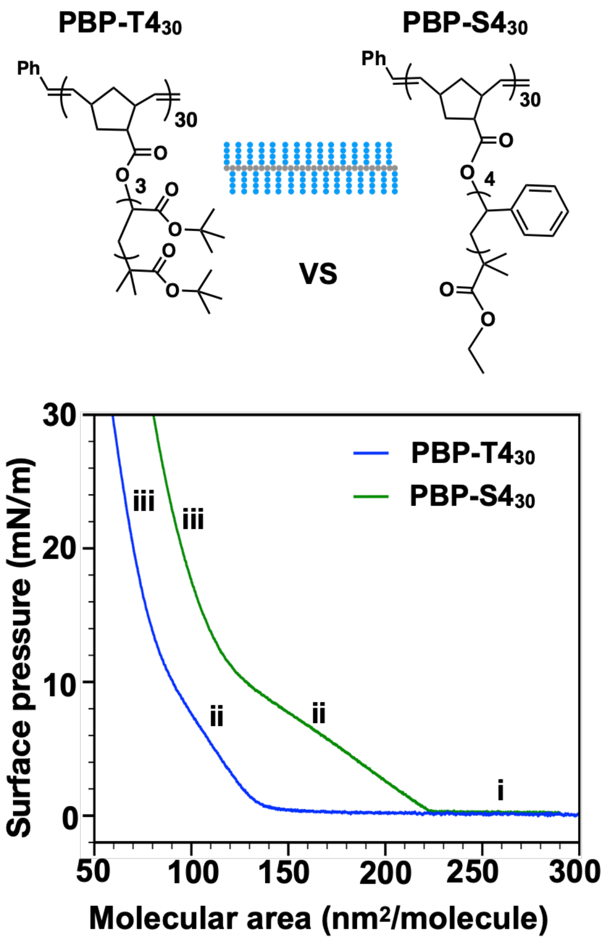

Figure S39. Langmuir-Blodgett surface pressure-area isotherm of **PBP-T4$_{30}$** (blue) and **PBP-S4$_{30}$** (green).

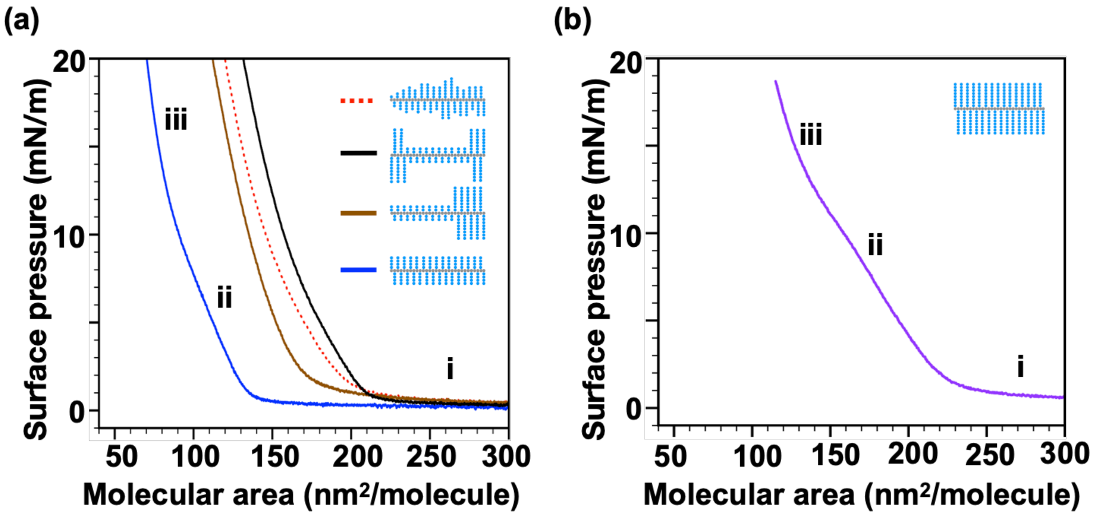

Figure S40. Langmuir-Blodgett surface pressure-area isotherm of (a) **BBP-oTBA4$_{30}$** (blue), **PBP-T4$_{30}$** (brown), **b-PBP-T2$_{20}$T8$_{10}$** (red dashed), and **b-PBP-T8$_5$T2$_{20}$T8$_5$** (black) (isomers with an average $N_{SC} \approx 4$ and $N_{BB} \approx 30$), (b) **PBP-T8$_{30}$** (purple) having a similar onset of surface pressure as **b-PBP-T8$_5$T2$_{20}$T8$_5$** in (a).

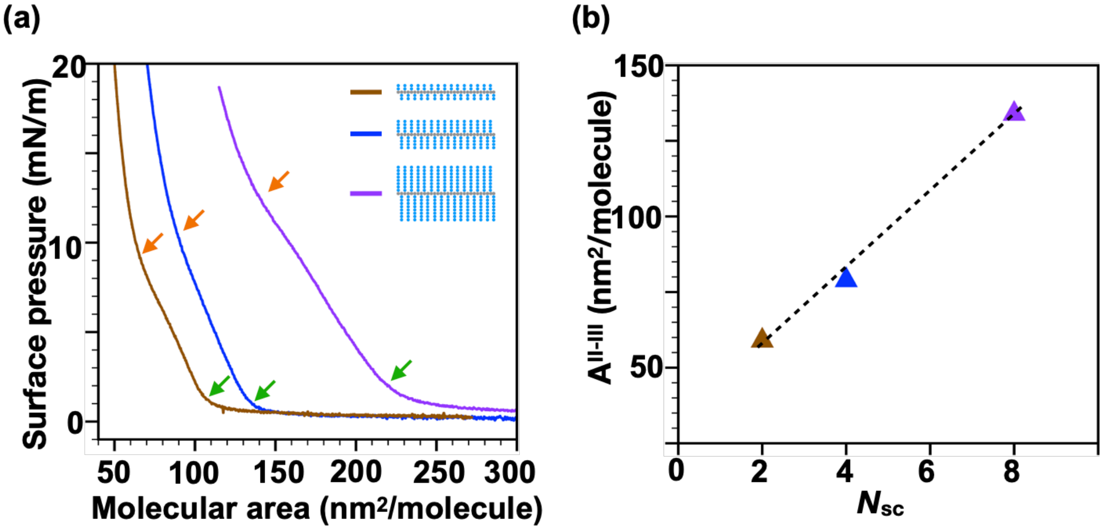

Figure S41. (a) Langmuir-Blodgett surface pressure-area isotherm of **PBP-T2$_{30}$** (brown), **PBP-T4$_{30}$** (blue), **PBP-T8$_{30}$** (purple). (b) Molecular area at the phase transition from liquid-to-solid phase scales according to N_{SC}.

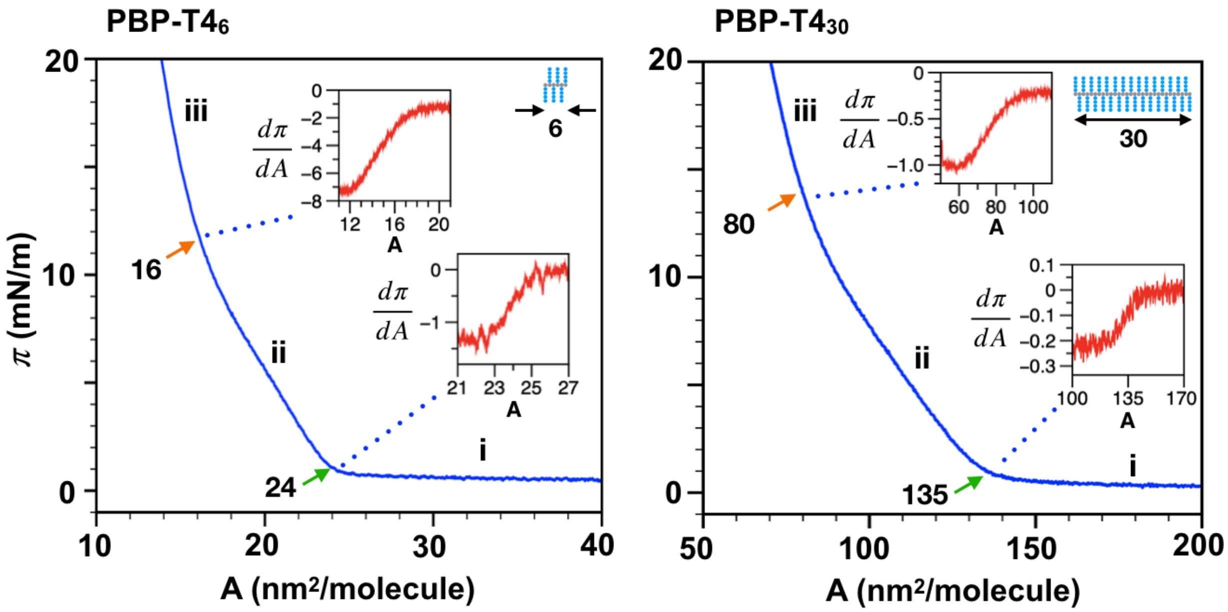

Figure S42. Langmuir-Blodgett (L-B) isotherms of **PBP-T4$_6$** and **PBP-T4$_{30}$**. Insets show the first derivative of isotherms to determine the values where the phase transition occurs.

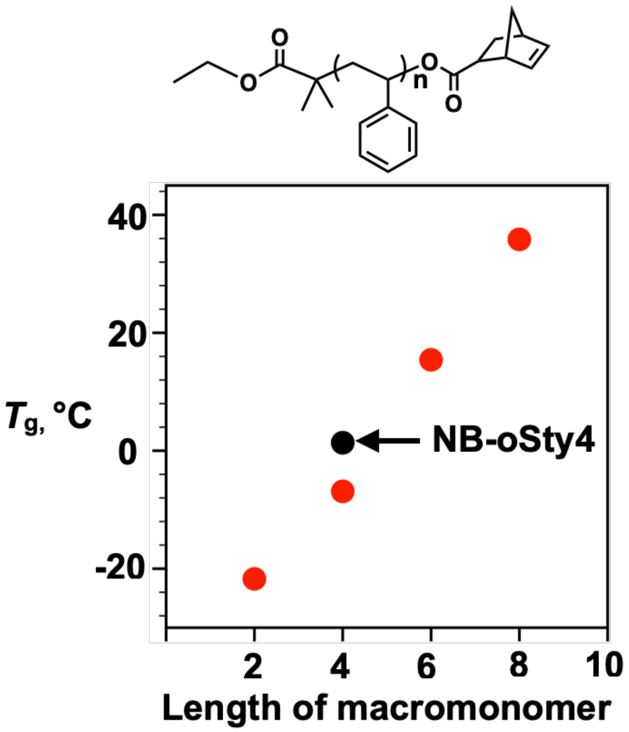

Figure S43. Glass transition temperature (T_g) of disperse **NB-oSty4** macromonomer (black) and discrete **S2 - S8** (red).

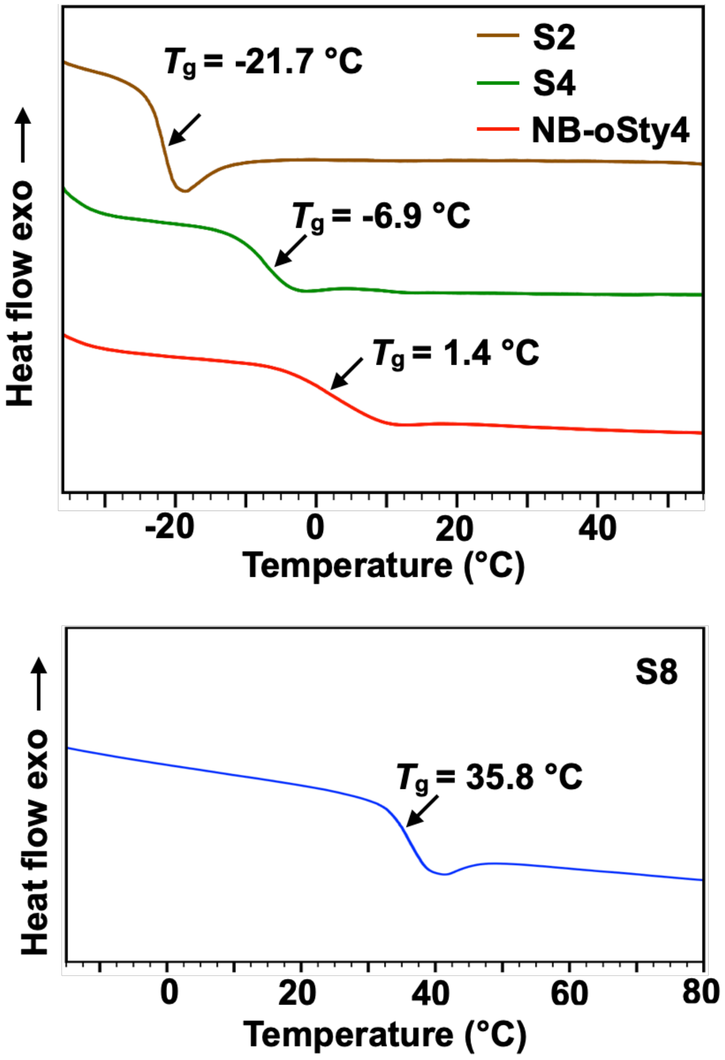

Figure S44. DSC traces of **S2** (brown), **S4** (green), **NB-oSty4** (red, control), and **S8** (blue) macromonomers.

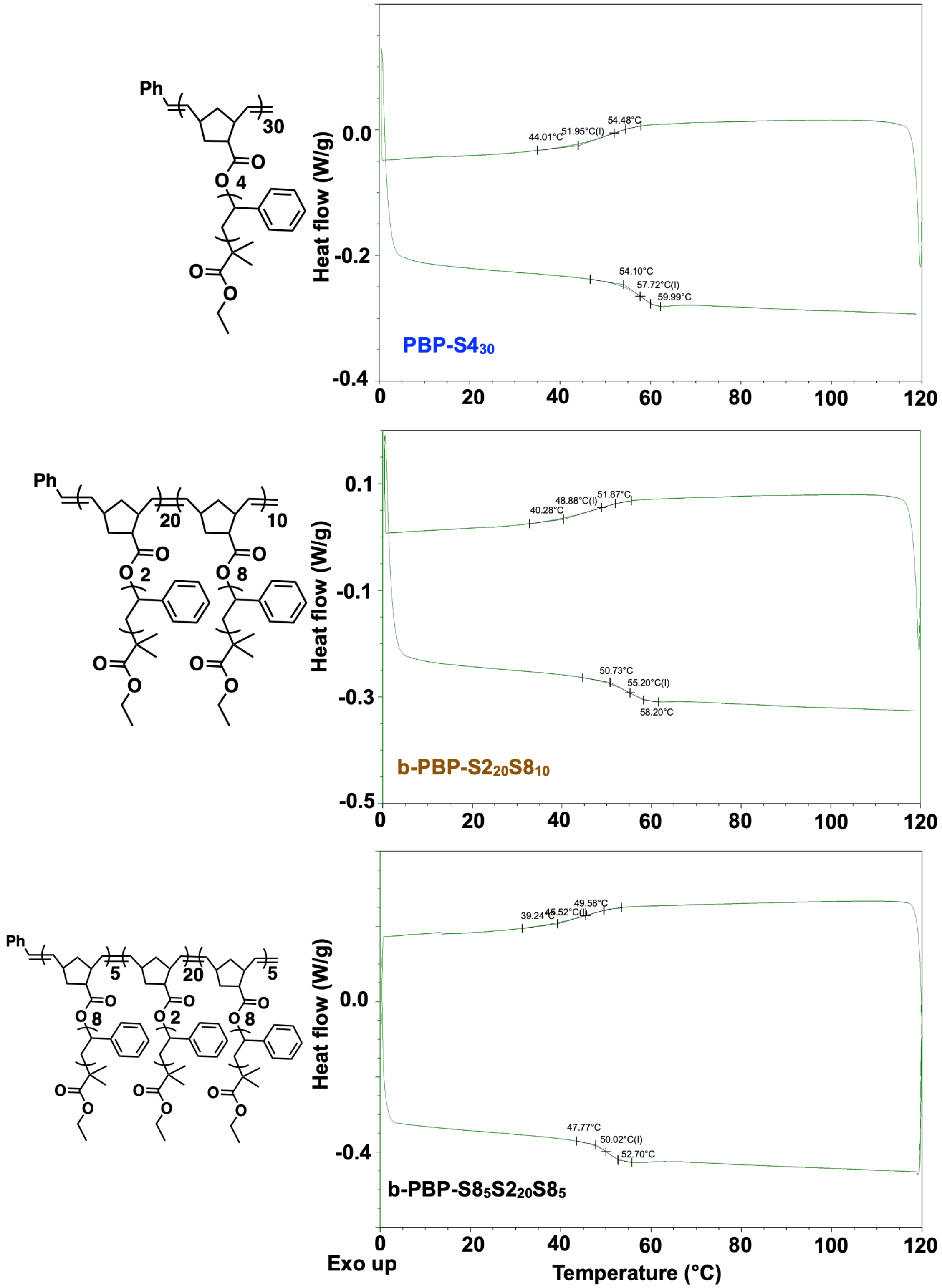

Figure S45. DSC traces of **PBP-S4$_{30}$** (blue), **b-PBP-S2$_{20}$S8$_{10}$** (brown), and **b-PBP- S8$_5$S2$_{20}$S8$_5$** (black).

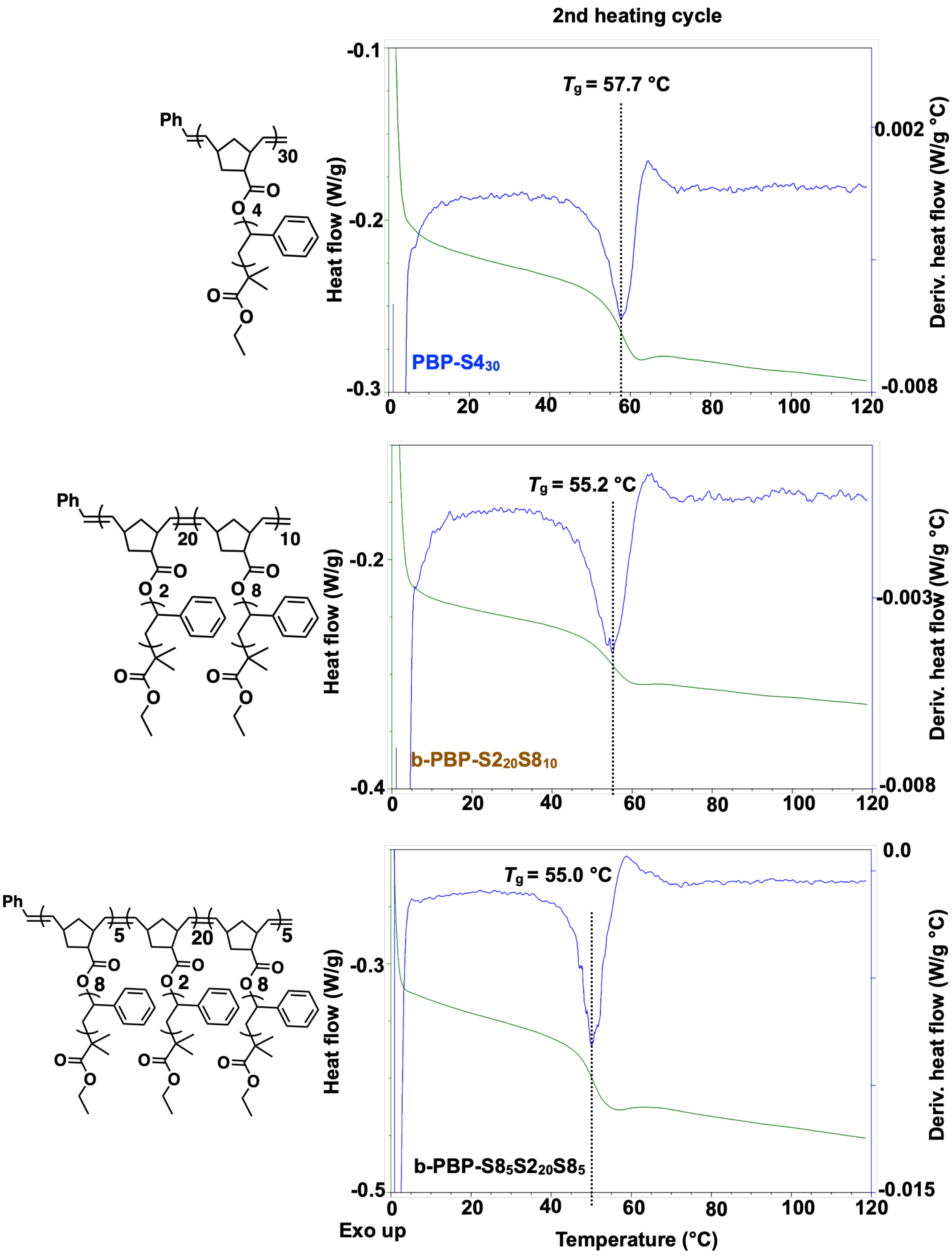

Figure S46. Differential DSC plots of **PBP-S4$_{30}$**, **b-PBP-S2$_{20}$S8$_{10}$,** and **b-PBP-S8$_5$S2$_{20}$S8$_5$**.

Modeling

Stochastic polymerization model of disperse macromonomers

The *grafting-through* polymerization for the disperse BBPs was modeled using the propagation rate constants found for each macromonomer (**Fig. 2e**, **Table S2**). A population of macromonomers was initiated with a specified number of initiators. At each time step, one macromonomer was added to each propagating brush. The probability (p_r) for a macromonomer with a specific length of *M* to react with the propagating brush end was proportional to the rate constants (k_p, function of macromonomer length), multiplied by its number (N_M), and a rate constant associated with the backbone length (B_i). The constant B_i was added to avoid random uncontrolled polymerization and reflect the exceptional controlled chain growth process in ROMP (**Equation S1**). The propagation continued until all macromonomers were consumed (*i.e.,* complete conversion).

$$p_r = \frac{k_p N_M B_i}{\sum_r k_p N_M B_i} \quad \text{(S1)}$$

Each brush species with backbone length of B_i is shown in a different color in Figure S47. Owing to the discrete nature of side chains, experimental data prove useful for fitting, with B_i as 1, 0.5, and 0.3 for N_{BB} = 2, 3, and 4, respectively, and ~ 0.1 for the remaining longer N_{BB}. The concentration for each macromonomer was obtained by deconvoluting the SEC trace shown in **Figure 1a**. The distinct multimodal profile is observed in both the experimental MALDI-ToF spectra of BBP-oTBA4 and the modeled histogram.

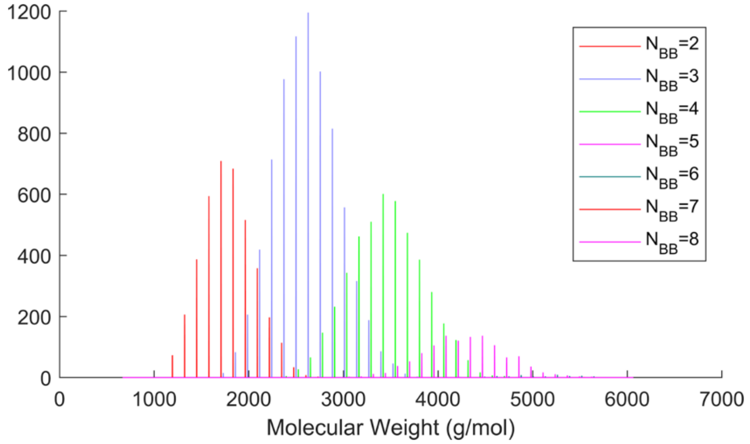

Figure S47. Simulated MALDI-ToF spectra exhibit the same multimodal structure seen in experimental data. Each population is separated by backbone length and color labeled.

Given the rates measured for each individual macromonomer, the side chain composition is not uniform along the backbone. As an example, the ratio of 6-mers to 3- and 4-mers is shown in **Figure S48**. Because the shorter macromonomers react faster, they are prevalent at the α-brush end of the BBP. Once depleted, the longer macromonomers prevalence increases at the ω-brush end of the BBP. This gradient of macromonomer lengths is significant with the 6-mer to 3-mer ratio being nearly three times larger when $N_{BB} \sim 5$ (near the end of polymerization) compared to the early stage of polymerization ($N_{BB} \sim 1$) (**Figure S48**).

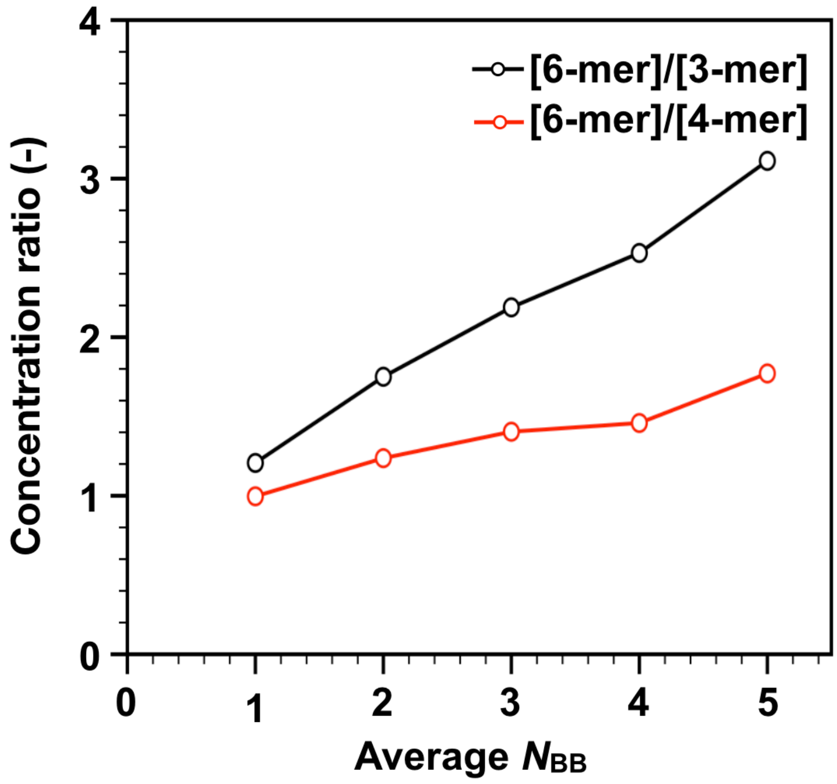

Figure S48. Ratio of 6-mers to 3- and 4-mers (side chain length) along the backbone during simulated polymerization runs. The data were averaged from 18917 brushes.

Coarse-grained model

A Kremser-Grest type model[4] was used to investigate the behavior of BBPs in Langmuir-Blodgett isotherms, as previously reported.[5] Backbone and side chain monomers were modeled using a single bead type, and the simulation was limited to two dimensions to mimic the trapping of the polymers at the air-water interface. Langevin dynamics with $\gamma = 0.5$ and a timestep of 0.01 using HOOMD-blue[6,7] at a reduced temperature of $T^* = 3$ (relative to polymer-

polymer interactions) was used. Polymers interacted via a Lennard-Jones potential with a cutoff distance of three times the particle diameter D, a harmonic bond potential, with $k_{bond} = 100$, $r_0 = 1$, and a harmonic angle potential with $k_{angle} = 30$, and $\theta_0 = 1.95477$ rad. Using a molar volume of 128.2 Å^3/mol (from a density of 1.00 g/cm^3) gives $D = 5.97$ Å. This can be used to compare simulations and experiments.

Interbrush pair potential energy. The potential energy between chains was calculated using the same coarse-grained model with Langevin dynamics at $T^* = 3$. Two PBP models with 30 backbone repeat units and 4 side chain repeat units were built in an extended conformation, minimized, and placed parallel to each other 15 units (~9 nm) apart. The side chains were allowed to move during the simulation, while the backbone chains remained fixed in place, keeping the chains parallel during the entire run. One of the PBPs was moved 0.01 units closer to the other every 100,000 timesteps. This was done for 10 different pairs. The same simulation was done for the bottlebrushes with disperse side chains, similarly with 10 different pairs. All disperse bottlebrushes were each built with randomly chosen side-chain lengths. The side-chain lengths were chosen from a uniform distribution in the inclusive range 0 to 8, averaging 4 side chains. The change in potential energy was measured every 10 timesteps and averaged for each step in the location of the backbone.

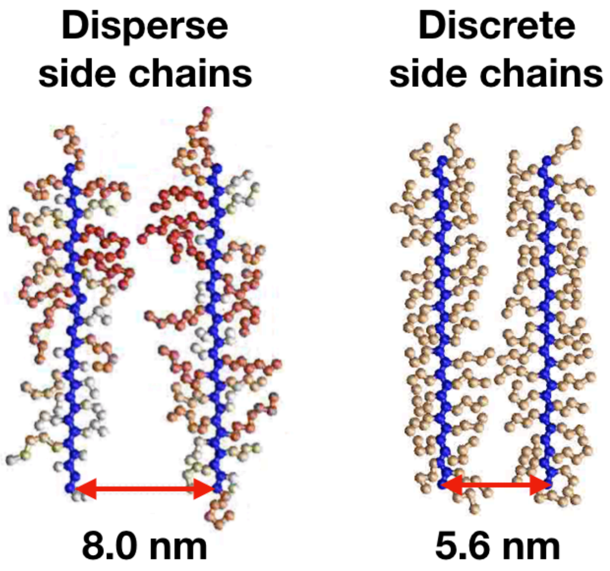

Figure S49. Coarse-grain model for the interbrush pair potential calculations. Parallel chains are moved closer together and potential energy calculated. The backbones are fixed in place until the next step in their movement occurs, keeping chains parallel.

Langmuir-Blodgett simulation. The polymers were built and initialized as done previously.[5] The simulation box was slowly shrunk over 10,000,000 timesteps from 119.4 nm × 119.4 nm to 73.1 nm × 73.1 nm to model the shrinking of the L-B trough. The disperse side chain BBPs (**Figure S50a**) show large backbone curvature due to the short side chain regions. In contrast, the PBPs have relatively straight backbones and stiffer dynamics (**Figure S50b**). In the case of a triblock sample, the backbone stiffness appears to be between that of disperse and discrete side chain bottlebrushes (**Figure S51**). As shown later, the difference in backbone stiffness between these bottlebrush samples could be explained by calculating their persistence lengths. We also note that there appears to be greater gaps between the dimer sections in the triblock sample than for discrete and disperse side chain samples. The simulated pressure vs. area per molecule plots (**Figure S52**) qualitatively agree with the experimental data. They are mapped to 1nm = 0.578 bead diameters, to match the molar volume of poly(*tert*-butyl acrylate). The discrete side chain bottlebrush sample (PBP) has the highest packing density, followed by the disperse-, then the triblock bottlebrush samples, in agreement with experimental results.

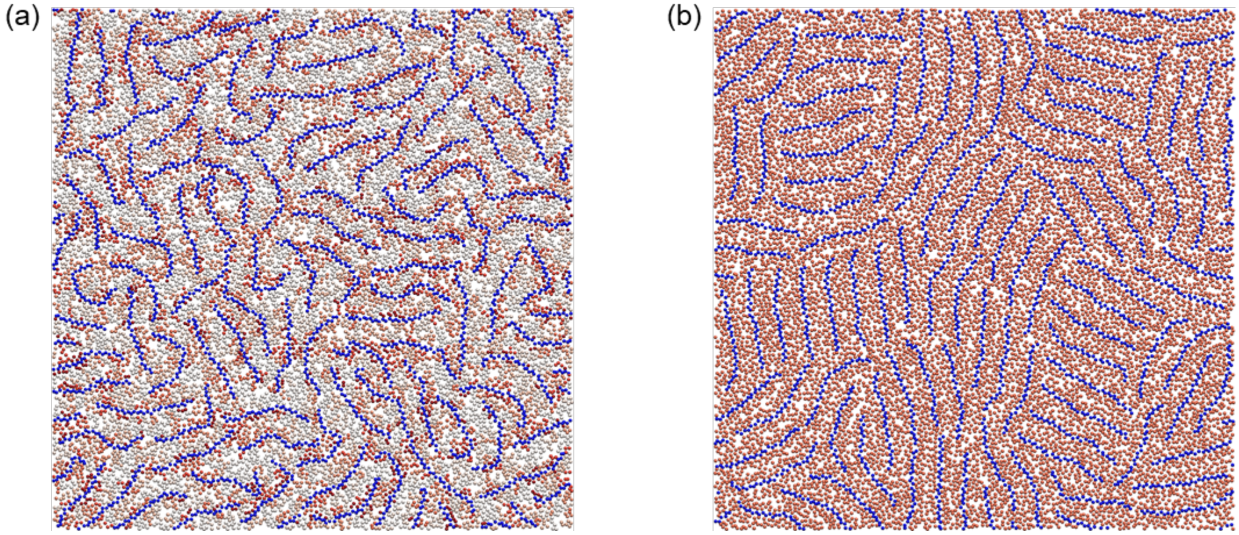

Figure S50. Snapshots of Langmuir-Blodgett simulation. Backbone beads are shown in blue, while side-chain beads are shown in white/red gradient, with red indicating short side chains and white indicating long side chains. (a) Bottlebrush sample with disperse tetrameric side chains (BBP). (b) Bottlebrushes with discrete tetrameric side chains (PBP).

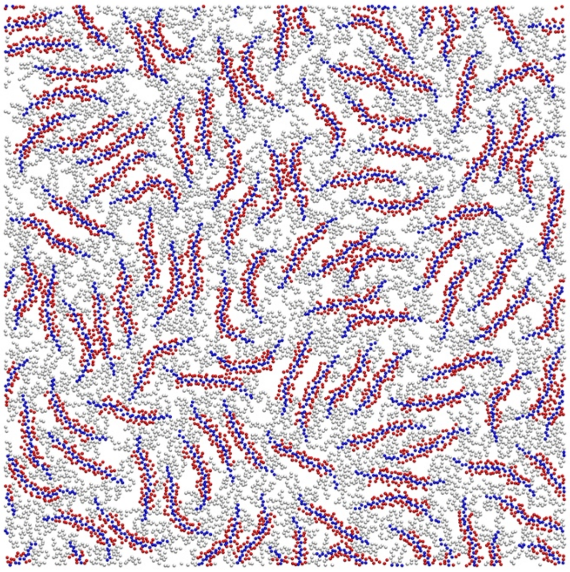

Figure S51. Snapshots of Langmuir-Blodgett simulation for a triblock model (**b-PBP-T8$_5$T2$_{20}$T8$_5$** analog). Dimer side chains, octamer side chains, and backbone beads are shown in red, white, and blue, respectively.

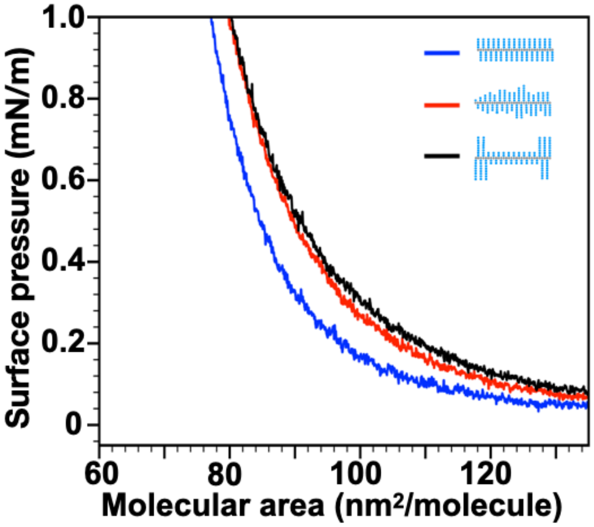

Figure S52. Langmuir-Blodgett pressure measurement for simulations in **Figure S50-S51**.

Persistence length The backbone curvature of bottlebrush samples was quantified through calculating their persistence length ℓ_P This is calculated using **Equation S2:**

$$ln(\langle \cos\theta \rangle) = \frac{-L}{\ell_P} + ln\,(a) \tag{S2}$$

Where θ is the angle between a vector tangent to the polymer at a certain position and a tangent vector at a distance L apart along the contour of the chain. The tangent is calculated as the vector between beads two bonds apart to consider the angle potential in the backbone. The angle brackets in Equation S2 indicate averaging over all possible vectors a distance L apart. **Figure S53** plots $\langle \cos\theta \rangle$ for the simulations shown in **Figures S50** and **S51** along with fits and the corresponding ℓ_P. As seen in **Figure S53**, the discrete bottlebrush (**PBP-T4$_{30}$** analog) has the highest persistence length, followed by the triblock (**b-PBP-T8$_5$T2$_{20}$T8$_5$** analog) and finally the disperse BBP-oTBA4$_{30}$ (control sample).

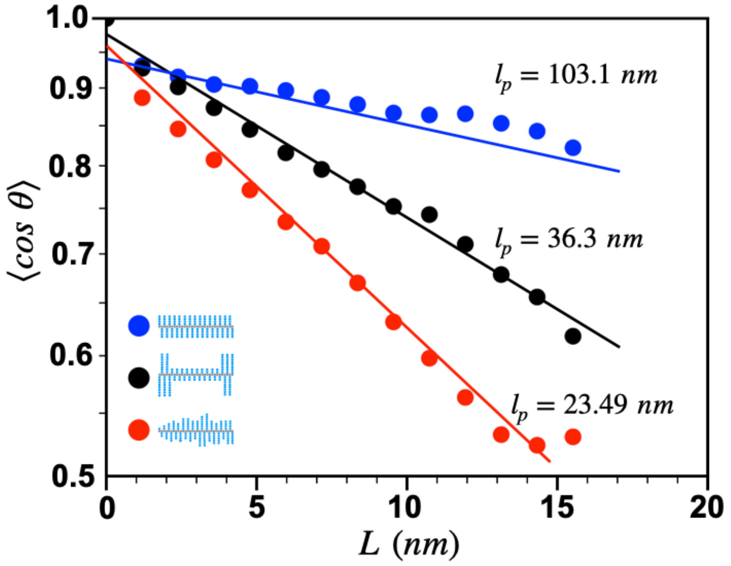

Figure S53. Plots of $\langle \cos\theta \rangle$ vs. L for the simulations shown in **Figure S50 and S51**.

Scheme S1: Comparison of the synthesis of conventional and discrete bottlebrush polymers

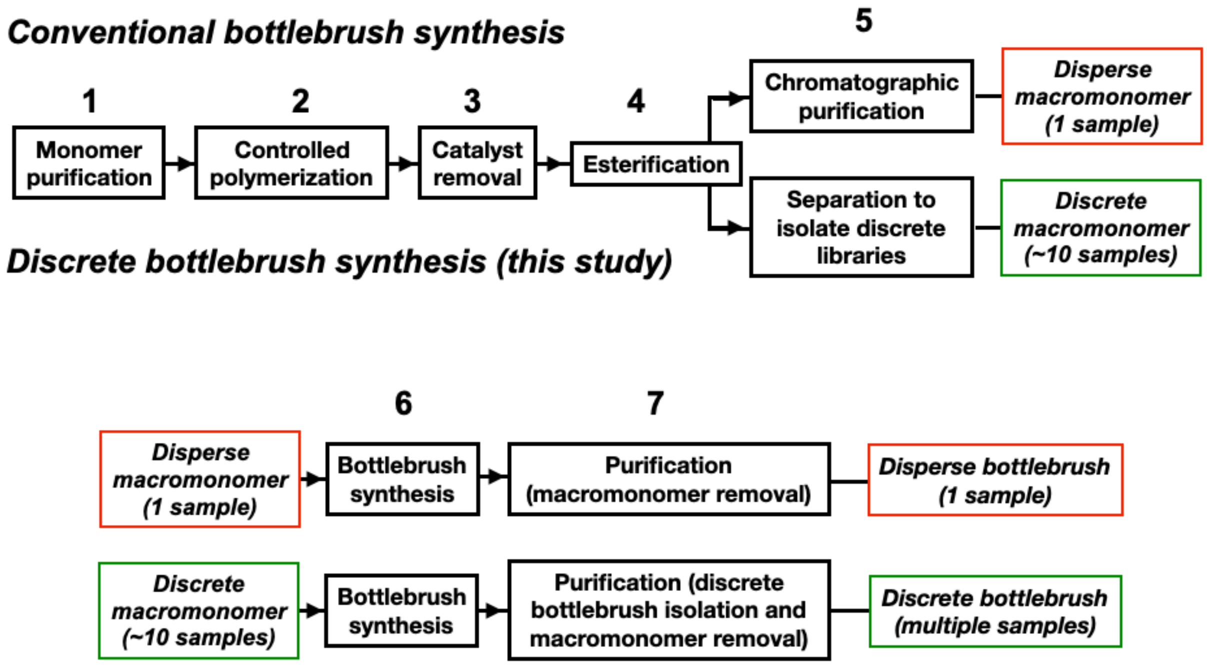

References

1. Kim, K.; Seo, M. G.; Jung, J.; Ahn, J.; Chang, T.; Jeon, H. B.; Paik, H.-J. Direct Introduction of Hydroxyl Groups in Polystyrene Chain Ends Prepared by Atom-Transfer Radical Polymerization. *Polym. J.* **2019**, *52* (1), 57–64.
2. Sanford, M. S.; Love, J. A.; Grubbs, R. H. A Versatile Precursor for the Synthesis of New Ruthenium Olefin Metathesis Catalysts. *Organometallics* **2001**, *20* (25), 5314–5318.
3. Radzinski, S. C.; Foster, J. C.; Scannelli, S. J.; Weaver, J. R.; Arrington, K. J.; Matson, J. B. Tapered Bottlebrush Polymers: Cone-Shaped Nanostructures by Sequential Addition of Macromonomers. *ACS Macro Lett.* **2017**, *6* (10), 1175–1179.
4. Kremer, K.; Grest, G. S. Dynamics of Entangled Linear Polymer Melts: A Molecular-dynamics Simulation. *J. Chem. Phys.* **1990**, *92* (8), 5057–5086.
5. Ogbonna, N. D.; Dearman, M.; Bharti, B.; Peters, A. J.; Lawrence, J. Elucidating the Impact of Side Chain Dispersity on the Assembly of Bottlebrush Polymers at the Air-water Interface. *J. Polym. Sci. A* **2021**, No. pol.20210565. https://doi.org/10.1002/pol.20210565.
6. Anderson, J. A.; Glaser, J.; Glotzer, S. C. HOOMD-Blue: A Python Package for High-Performance Molecular Dynamics and Hard Particle Monte Carlo Simulations. *Comput. Mater. Sci.* **2020**, *173*, 109363.
7. Glaser, J.; Nguyen, T. D.; Anderson, J. A.; Lui, P.; Spiga, F.; Millan, J. A.; Morse, D. C.; Glotzer, S. C. Strong Scaling of General-Purpose Molecular Dynamics Simulations on GPUs. *Comput. Phys. Commun.* **2015**, *192*, 97–107.
